# What do “barbarians” eat? Integrating ceramic use-wear and residue analysis in the study of food and society at the margins of Bronze Age China

**DOI:** 10.1371/journal.pone.0250819

**Published:** 2021-04-29

**Authors:** Karine Taché, Yitzchak Jaffe, Oliver E. Craig, Alexandre Lucquin, Jing Zhou, Hui Wang, Shengpeng Jiang, Edward Standall, Rowan K. Flad

**Affiliations:** 1 Department of Historical Sciences, Université Laval, Laval, QC, Canada; 2 Zinman Institute of Archaeology, University of Haifa, Haifa, Israel; 3 Department of archaeology, BioArch, University of York, York, United Kingdom; 4 Gansu Institute of Archaeology, Lanzhou, Gansu, People’s Republic of China; 5 Department of Cultural Heritage and Museology, Institute of Archaeological Science, Fudan University, Shanghai, China; 6 School of Archaeology, Oxford University, Oxford, United Kingdom; 7 Department of Anthropology, Harvard University, Cambridge, MA, United States of America; University at Buffalo - The State University of New York, UNITED STATES

## Abstract

The Siwa archaeological culture (ca. 3350 and 2650 cal yr BP) has often been associated with the tribes referenced in textual sources as Qiang and Rong: prized captives commonly sacrificed by the Shang and marauding hordes who toppled the Western Zhou dynasty. In early Chinese writings, food plays a key role in accentuating the ‘sino-barbarian’ dichotomy believed to have taken root over 3000 years ago, with the Qiang and Rong described as nomadic pastoralists who consumed more meat than grain and knew little of proper dining etiquette. To date, however, little direct archaeological evidence has allowed us to reconstruct the diet and foodways of the groups who occupied the Loess Plateau during this pivotal period. Here we present the results of the first ceramic use-wear study performed on the Siwa *ma’an* jars from the site of Zhanqi, combined with the molecular and isotopic characterization of lipid residues from foodcrusts, and evidence from experimental cooking. We report molecular data indicating the preparation of meals composed of millet and ruminant dairy among the Siwa community of Zhanqi. Use-wear analysis shows that Zhanqi community members were sophisticated creators of ceramic equipment, the *ma’an* cooking pot, which allowed them to prepare a wide number of dishes with limited fuel. These findings support recent isotope studies at Zhanqi as well as nuance the centrality of meat in the Siwa period diet.

## Introduction

Rain-fed agriculture in the Western Loess Plateau of northern China developed by around 7800 cal yr B.P. and involved the adoption of foxtail (*Setaria italica*) and broomcorn millet (*Panicum miliaceum*) along a vast east-west transect centered on the Yellow and Wei Rivers [1,2]. After around 4000 years B.P. and linked by some to environmental deterioration following the warm-humid Holocene climatic optimum, millet agriculture decreased in importance in some areas, supplemented by pastoralism as a key subsistence element [3–7]. Concomitant and perhaps related changes in the cultural landscape at ca. 3500 B.P. include the decline of the Qijia cultural sphere, a tradition centered in eastern Qinghai and western Gansu provinces of the People’s Republic of China (PRC) [8,9], and the migration of what are seen as nomadic communities into the Loess Plateau [10–13]. The Siwa 寺洼 archaeological culture is often taken to represent one example of these pastoral and nomadic communities. Siwa material culture is characterized by a unique double-handled saddle-shaped mouth container, known as the *ma’an* 马鞍 jar. Within the widespread tradition of linking archaeological cultures with ethnic groups, the Siwa culture has been associated with the *Qiang* 羌 people: prized captives commonly sacrificed by the Shang, or the *Rong* 戎 tribes: marauding hordes who toppled the Western Zhou dynasty [14].

In early Chinese writings, food often plays a key role in accentuating a ‘sino-barbarian’ dichotomy believed to have taken root over 3000 years ago. This dichotomy opposed the members of the Ancient Chinese people/world (i.e., *Zhonggu*o 中国 *Hua 崋*, *Xia* 夏) and the cultural others, or aliens, including the Qiang and Rong, but also the *Man* 蠻, *Yi* 夷 and *Di* 狄. The Qiang and Rong “aliens/barbarians” are sometimes described as peoples who consumed more meat than grain and knew little of proper dining etiquette [15–17]. A notable example is found in the Wangzhi chapter of the *Liji* or classics of rites: “the Eastern Yi eat their food without it being cooked with fire…and so do the southern Man…whereas the western Rong and northern Di abstain from cereals” (translation in [18]).

This divide, and indeed the very definition and use of these terms (Barbarian, Chinese, Huaxia Zhongguo, etc.) are quite loaded and have a long history of academic translation and integration in cultural traditions and frameworks [15,17,19]. Presently, little to no direct archaeological evidence exists to allow us to reconstruct the diet of the groups who occupied the Loess Plateau during this pivotal period attributed to the Siwa culture and ranging between ca. 3350 and 2650 cal yr B.P. Nevertheless, the Siwa archaeological culture, and the people they are identified with, are often assumed to have led a nomadic and pastoral subsistence lifeway, consuming a diet rich in animal products and low in grains [8,11,20,21]. This characterization, however, is not predicated on a systematic analysis of animal and plant remains from robust domestic contexts, but is a reflection of mostly later historical realities of Steppe and Sown anachronistically projected onto archaeological cultures of the deeper past. In fact, even with the limited archaeological evidence that does exist, it is clear that Siwa communities consumed grain as a main component of their diet [22]. The few faunal remains from securely dated contexts associated with Siwa materials are dominated by ruminants, such as bovines, caprines (sheep and goats) and some equids. At the cemeteries of Jiuzhan 九站 (ca. 3350–2650 B.P.) and Lanqiao 栏桥 (ca. 3300 B.P.), for example, sheep and cow bones (often heads) were found interred in a number of graves [23,24]. Rudimentary zooarchaeological work on the Xujianian 徐家碾 cemetery (ca. 3200–3000 B.P.) found taxa of sheep/goat, cow, horse, and pig [25]. The most common animals found were cattle (>85% of all remains) instead of sheep/goat, as had been expected. The above data is related to mortuary customs rather than everyday diet and subsistence practices, yet it shows the importance of cattle to the communities in question. Very few domestic contexts have been excavated and reported. At Ya’ar (艹+也儿), cattle dominate the bone materials unearthed from a number of domestic refuse pits [26]. In addition to the unmodified bones representing remnants of past meals, some of the cattle bones have been made into tools. Other animals found at Ya’ar include several specimens of deer, some sheep and small birds.

Recently, the incorporation of new research methods, particularly stable isotope analysis conducted mostly on human skeletal data, have begun to provide insights into prehistoric diets in northwestern parts of the PRC. To date such studies have focused on sites in the Hexi corridor north of the area inhabited by the Siwa archaeological culture. In the Hexi region broad trends have been detected, such as a shift from a primarily C_4_ diet to one incorporating greater amounts of C_3_ plants sometime in the first half of the 2^nd^ millennium BCE (e.g. [4,27–30]). Work at the Siwa culture Zhanqi 占旗 cemetery in Gansu, China, has found a mixed C_4_ and C_3_ diet (representing a possible decrease of millet consumption) coupled with higher δ^15^N thought to reflect increased meat intake [4,31].

Existing archaeological data are limited. Many of the Siwa sites are dated solely on the basis of ceramic chronologies, and where C^14^ dates exist, there are typically few of them [22]. Furthermore, stable isotope studies of skeletal remains seldom include the analysis of zooarchaeological and botanical samples necessary to provide secure comparative baselines with which one can interpret these results (an issue raised in many such studies, e.g. [4,29]). They nevertheless point to possible changes such as an increase in diets rich in animal proteins, to be further investigated. To inform results of isotope studies, we must turn to other direct lines of evidence on diet and foodways. Analysis using modern analytical chemical methods of lipid residues from pottery is a powerful tool to identify a range of animal and plant resources processed in pots, thereby providing new insights into past subsistence strategies, including changes in herding and farming practices. The present study provides direct molecular and isotopic evidence for the processing of millet, mixed with ruminant dairy fats and possibly other food sources, in ten *ma’an* jars from the Zhanqi cemetery in Gansu, China. These results are combined with large scale use-wear analysis and experimental cooking to provide a better understanding of the diet and cooking practices associated with the Siwa cultural groups, and secondarily verify some of the presumptions made about their foodways.

## Zhanqi cemetery

**Fig 1:**
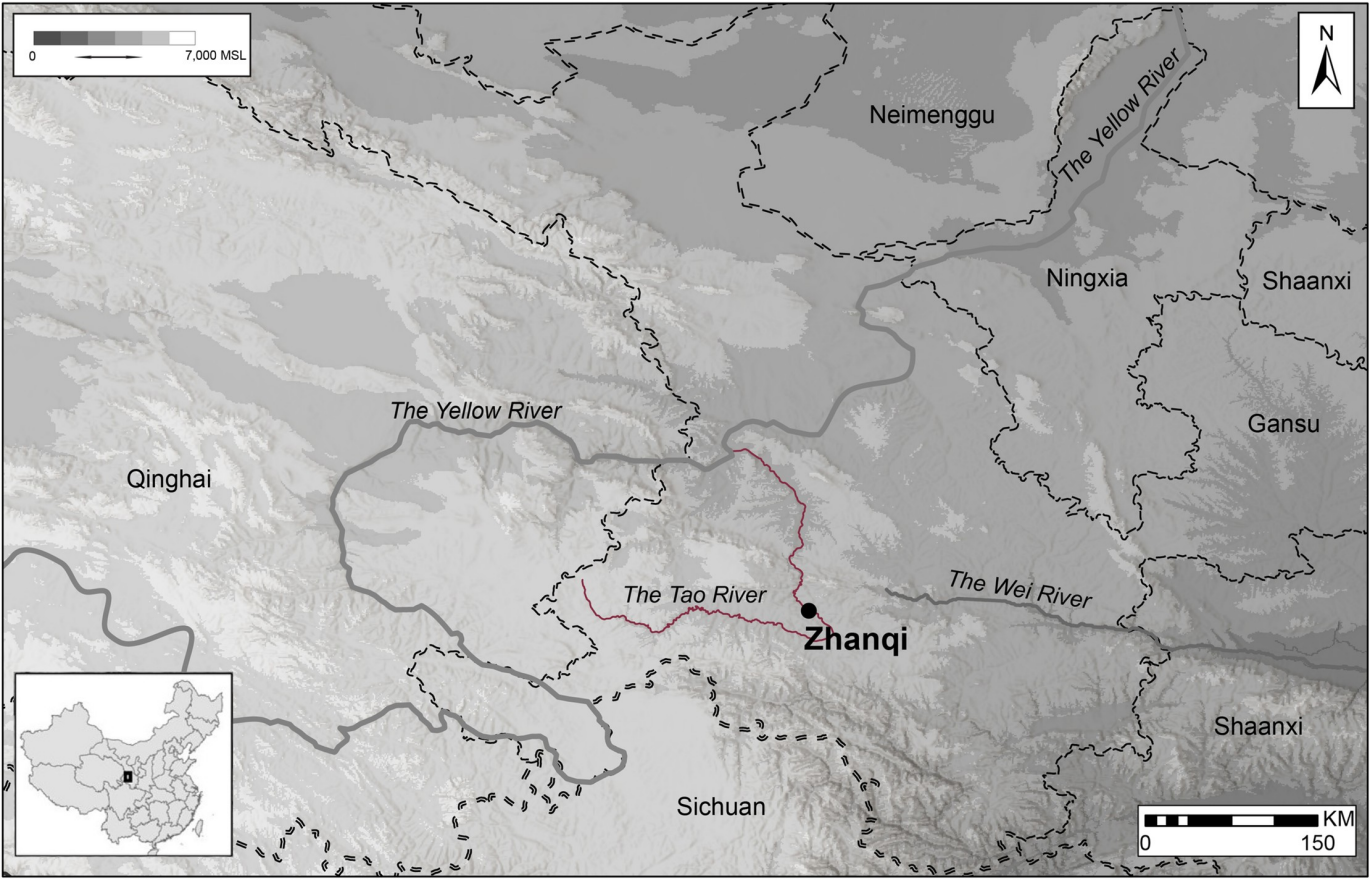
Location of the Zhanqi site. This map was made in ArcGIS software with original materials as well as open access files obtained from China historical GIS database at Harvard University—http://sites.fas.harvard.edu/~chgis/.

The Siwa data from the site of Zhanqi presented here were collected as part of a collaborative project between the Gansu Provincial Institute of Archaeology and Cultural Heritage, Peking University, and Harvard University (see [32,33]). Zhanqi is located 500 meters east of Zhanqi village along the Tao River valley in modern day Min County, Gansu Province (Fig 1). Data analyzed here are from a rescue excavation conducted by the Gansu province institute of archaeology at this site prior to a dam construction. The excavation revealed 66 human graves, 2 residential structures, 10 refuse pits and 2 sacrificial pits [34]. A uniform burial pattern characterizes this cemetery. Inhumations are placed in rectangular graves, most equipped with a stepped platform ledge (*ercengtai* 二层台) holding artifacts. Some graves were fitted with hallowed niches found over the head of the tomb’s occupant. The dominant grave orientation is east-west, but a small number of burials are oriented along a north-south axis. The vast majority of tombs contain single burials where the deceased are placed on their backs with their bodies extended. Many skeletons are dismembered, reflecting a situation where secondary burial practices might have been common. Bronze artifacts, including ornaments and many weapons (e.g., *ge* 戈axes, swords, knives of steppe style *mao* 矛 spears and halberds) were placed to the side of the deceased denoting the importance of military status for the elites of this Siwa community [34].

Of the two residential structures found at the site, the best-preserved (F1) is semi-subterranean and round-shaped. The door faced north and was fitted with a large stone. Inside, a hearth and a number of post holes were found as well. The earthen floor is tightly packed and might have been created by the rammed-earth (*hangtu* 夯土 [34] style. Structure F2 was not as well preserved. Yet the internal findings included a well formed tamped earth floor with textile and ceramic imprints. The site included 10 waste pits yielding a mix of ceramic sherds, stone tools, bones and other artifacts. Two supposed ritual features were found within the residential portion of the site. One is a circular platform, two meters in diameter, that comprised a large stone slab where a number of ceramic vessels containing unidentified animal bones and burned earth were found. The second ritual feature is a platform with burned earth and a number of ceramic jars with unidentified animal bones and ash found in them [34]. Among the representative Siwa culture artifacts found at the site, *ma’an* jars are the most distinctive.

### Ethics statement

**Fig 2:**
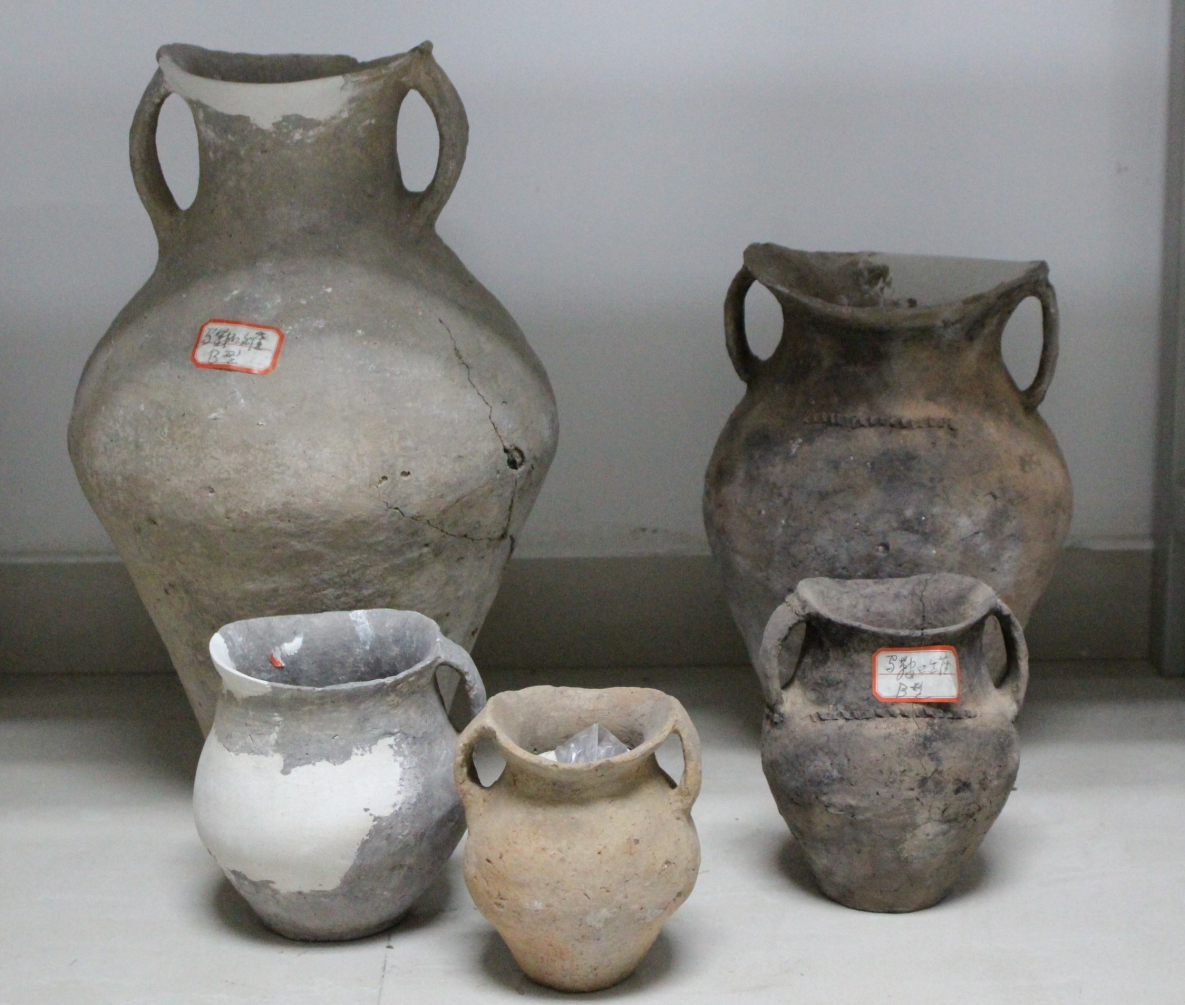
A selection of *ma’an* style vessels from the Zhanqi cemetery.

The data presented here were collected as part of the project “Cultural and Social Change during the Neolithic and Bronze Age along the Tao River in Gansu”, approved by the National Bureau of Cultural Relics in 2015 (Cultural Relics Protection Document 2015 - #3493). Permission to conduct use-wear and residue analysis was granted by “The Gansu Provincial Institute of Archaeology and Cultural Heritage". The individual in this manuscript (S2 File, Fig 2) has given written informed content, as outlined in PLOS consent form, to publish these case details.

## Siwa foodways and ceramic use-wear analysis

To date, several thousand *ma’an* vessels have been recovered, making it a hallmark of the Siwa archaeological culture [35]. Like many other Neolithic and Bronze Age cultures in China, distinctive Siwa culture material comes quite exclusively from burial contexts. Only a handful of non-mortuary *ma’an* jars have been excavated [8]. The *ma’an* jars and other pottery were made by tournette (or “slow wheel”), low fired and often characterized by what are often called ‘fire clouds’ (uneven color) on the exterior. This is taken, by some, to denote an inferior ceramic craft compared to that of the contemporaneous Shang and Zhou to the east (e.g. [36])–a situation that may not be surprising, if Siwa communities were engaged in mobile pastoralism and consequently may have had fewer ceramic specialists. As with most jars and jugs, the function of the *ma’an* jar is often reconstructed as a storage vessel, likely for containing grain or liquids. The *ma’an* jar references a general shape, but these vessels come in many varieties and sizes (Fig 2).

The most common cooking vessel in Chinese archaeology, the *li* 鬲, or cooking tripod vessel, is also found in Siwa culture sites (though again mostly in graves) and is assumed to be the main cooking vessel of this period. On further analysis (discussed below), medium *ma’an* jars with flat bases and thick walls were in fact used for cooking, whereas small *ma’an* juglets and large jugs were used for other purposes (perhaps indeed holding liquids or grains). A large number of the medium vessels were clearly exposed to intense heat sources for prolonged periods of time, most likely as a result of cooking practices. Furthermore, the texture and material composition of these vessels is expected of a cooking vessel: rough textured, tempered and brittle [37].

**Fig 3:**
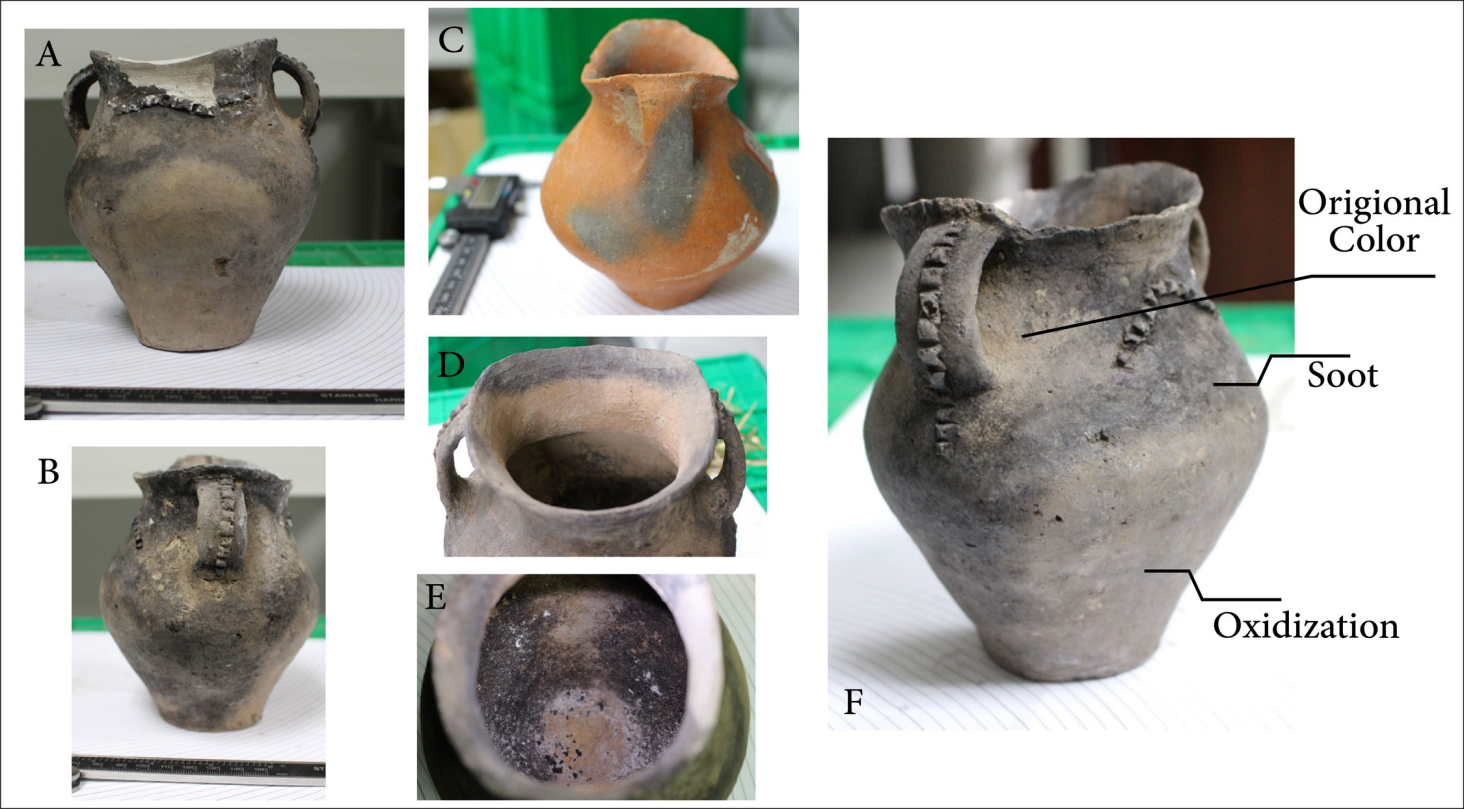
Observed use-wear patterns on Siwa ma’an cooking vessels. A. Frontal image of *ma’an* jar showing external soot pattern (base is about 4.5 cm across). B. Handle-sided view of *ma’an* jar showing external soot pattern. C. Changes in paste color resulting from fire clouding or other production processes. D. Internal carbonization on upper portion of lip. E. Internal carbonization on walls. F. *Ma’an* jar with use-wear pattern (soot and oxidation).

The location of use-wear alteration can inform cooking habits. Vessel placement with respect to heat sources and the intensity of the fire can also be reconstructed from these alterations on the surfaces of pots [38]. Hally [39] pioneered important methods for the interpretation of cooking techniques such as simmering and boiling [40–44]. Use-wear analysis conducted on the entire assemblage of *ma’an* jars recovered from the Zhanqi site revealed a recurring pattern on many vessels: a heavy patch of soot is seen on the narrow side of the vessel where the handles were attached, and a distinct patch of soot on both of the opposite sides–where the rim is shaped like a saddle–exists as well. This band of soot extends from roughly the shoulder to the rim. Under this band, around the carination, is a bright red or greyish patch of oxidation down to the base. The internal pattern is reversed: the lips are covered with carbonization on the saddle-shaped side but not on the side with handles, and inversely a thick layer of carbonized food covers the entire portion of the saddle shaped side. The base, surprisingly, is clean (Fig 3).

Reconstructing how this pattern was created requires understanding how use-wear marks accumulate on vessels with respect to both exposures to heat sources and the type of cooking that took place. Skibo [38,45] has argued that by observing external soot and oxidization patterns, as well as internal carbonization and charred food remains, two main modes of cooking can be identified: the *wet mode* (stewing, boiling etc..) and the *dry mode* (such as roasting). Oxidation marks are commonly formed were the vessels is subject to intense direct heats; thus soot is often found on, around and along the bottom part of the vessel exterior. When cooking with little liquid or fat, i,e. the dry mode, interior walls and food cooked can exceed 300 degrees Celsius, leading to carbonized foods permeating the vessel’s walls [38].

**Fig 4:**
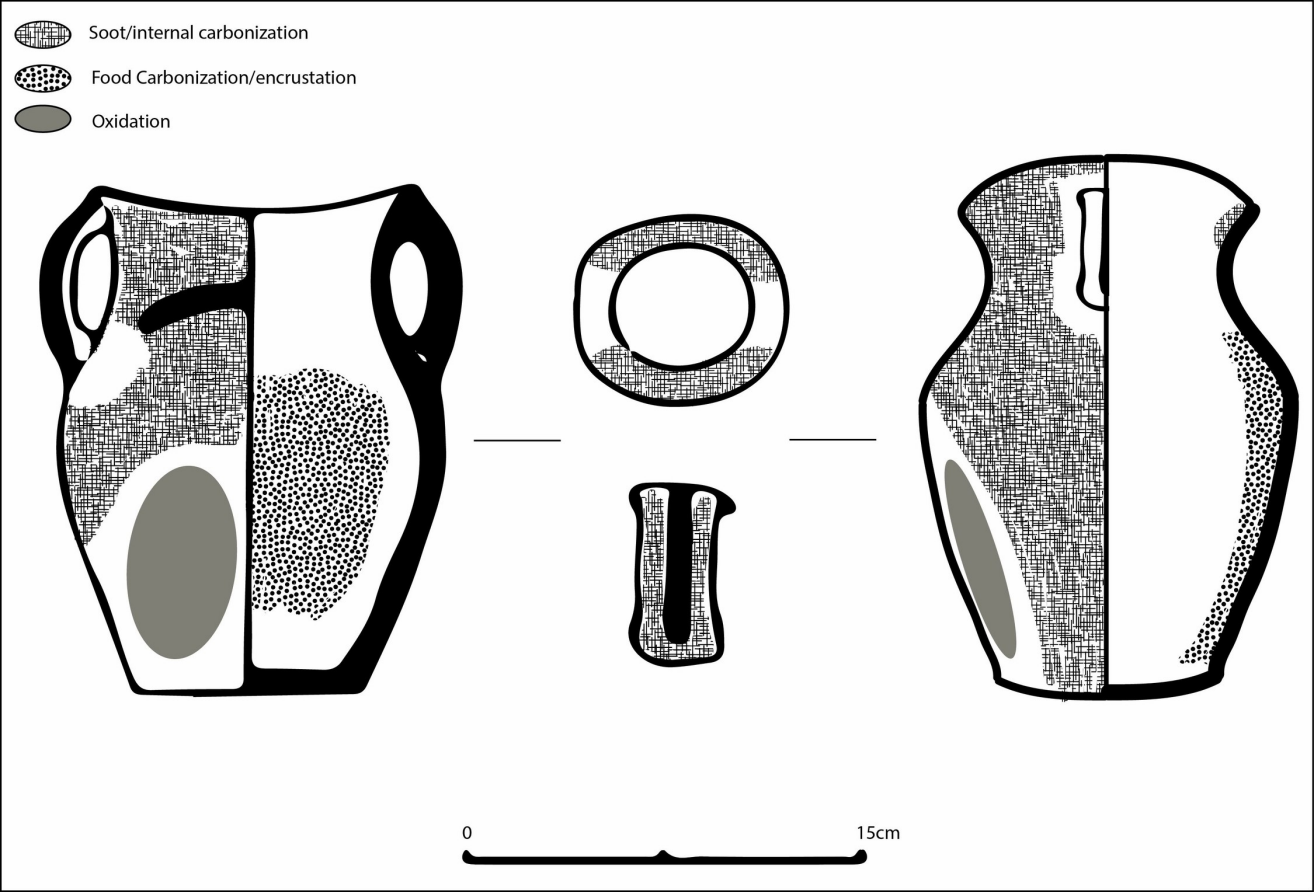
Schematic image of dominant use-wear pattern observed on Siwa *ma’an* cooking vessels.

**Fig 5:**
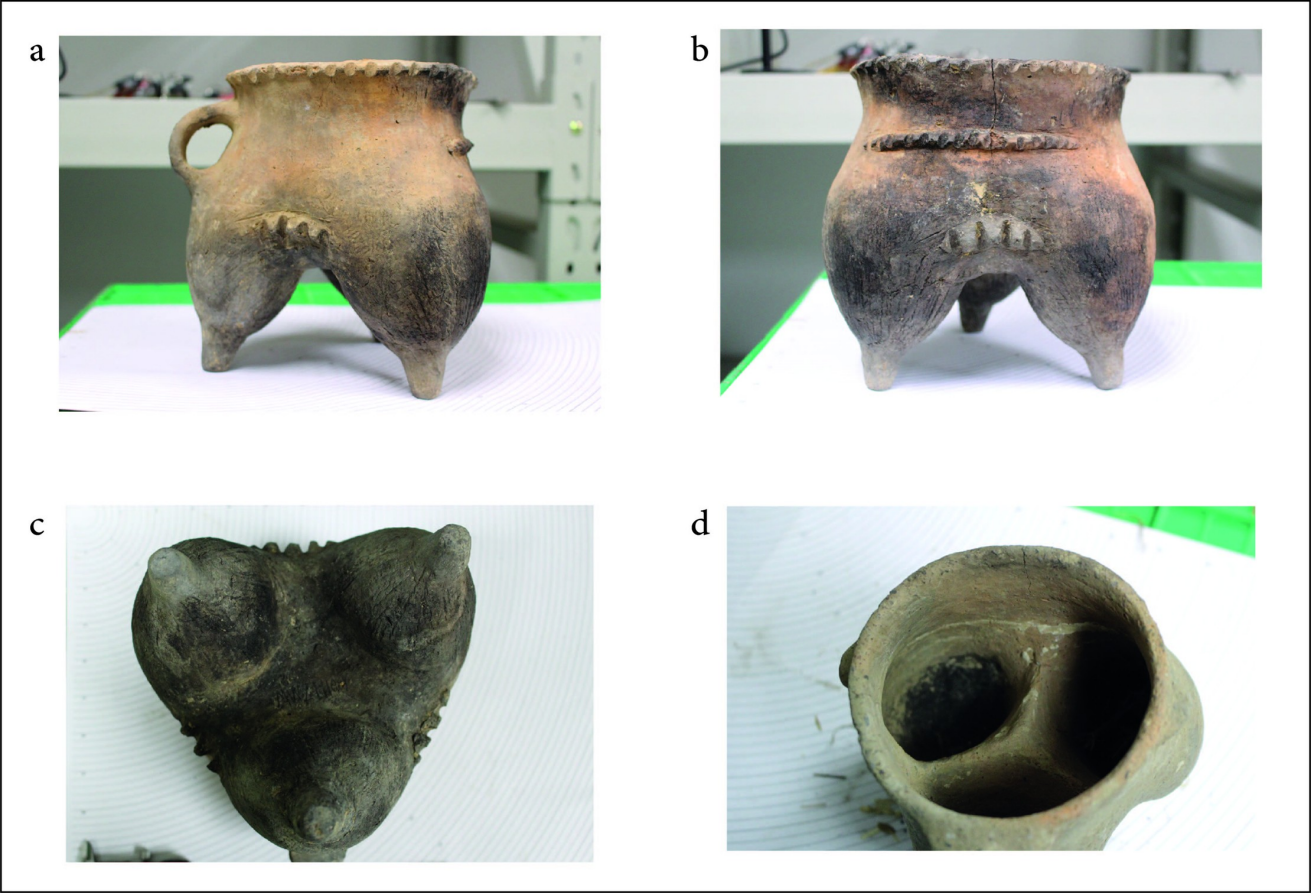
Use-wear alteration marks of Zhanqi *li* vessels. A. Soot remains extending from the belly down to the foot. B. Soot remains on the handle perpendicular to the side exposed to the fire. C. Heavy sooting on the base and oxidation on the feet (nipple), indicating placement base side down in the fire. D. Heavy carbonization can be seen in each of the tripod’s three feet, an expected outcome of a cooking vessel placed in the fire or exposed to a fire source base side down from which enough liquid evaporated to allow food particles to burn and char.

**Fig 6:**
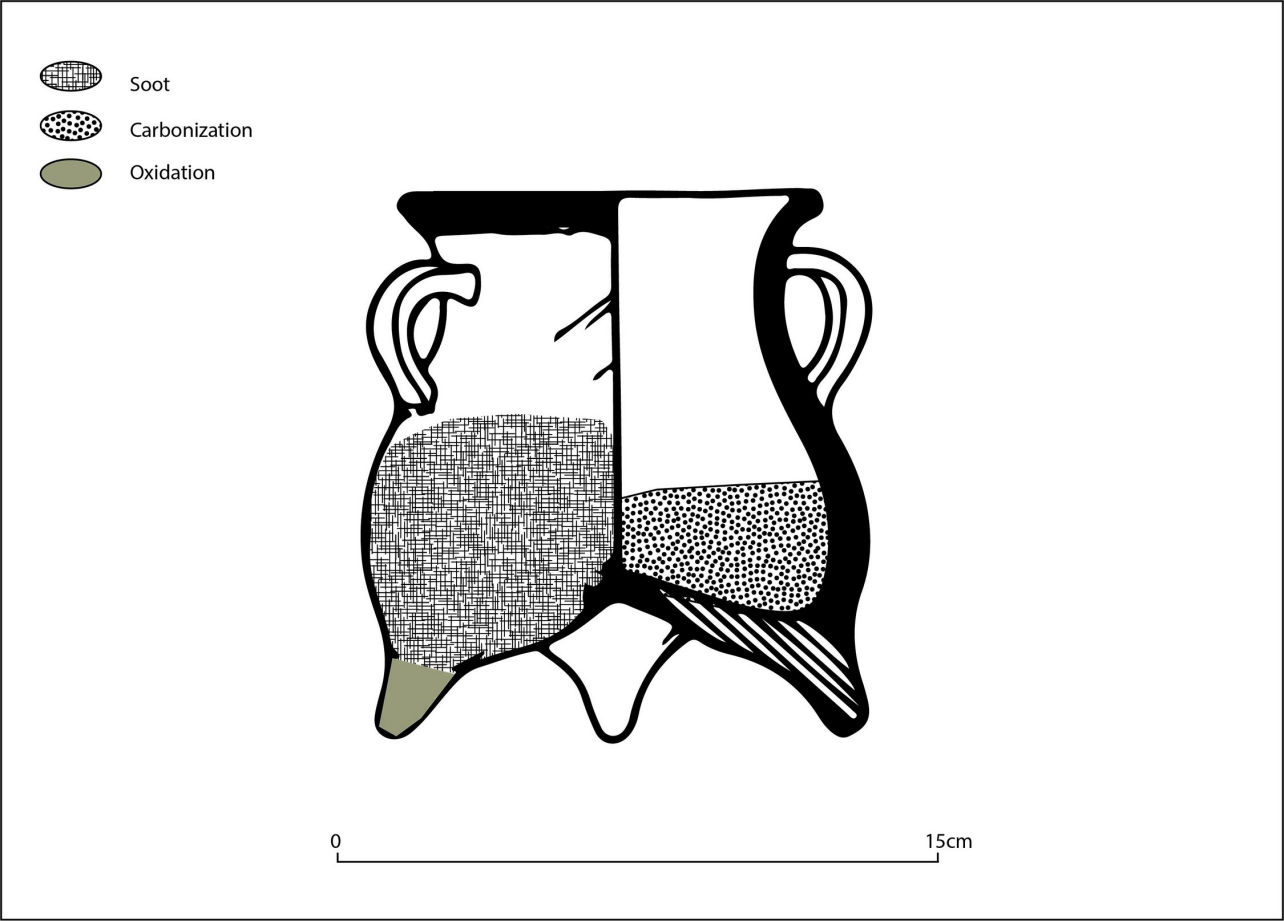
Schematic image of dominant use-wear pattern observed on Siwa *li* cooking vessels.

The dominant use-wear pattern observed at Zhanqi (Fig 4) would form if the vessel was exposed to intense heat where little oil or liquid was applied (either initially or over time as the liquid evaporated and the vessels continued to be exposed to direct heat), resulting in charred remains throughout the vessel. If the vessel was placed right-side up in the fire we would expect to find, internally, charred remains on the bottom of the pot and an oxidation patch on the vessel’s external base leading up to the belly. This sort of pattern can in fact be seen on the few *li* vessels (n = 9) found at the cemetery (Figs 5 and 6; and see [46] for an example of this expected pattern on Zhou period *li* vessels from Shandong–rough contemporaries of the Siwa material).

**Fig 7:**
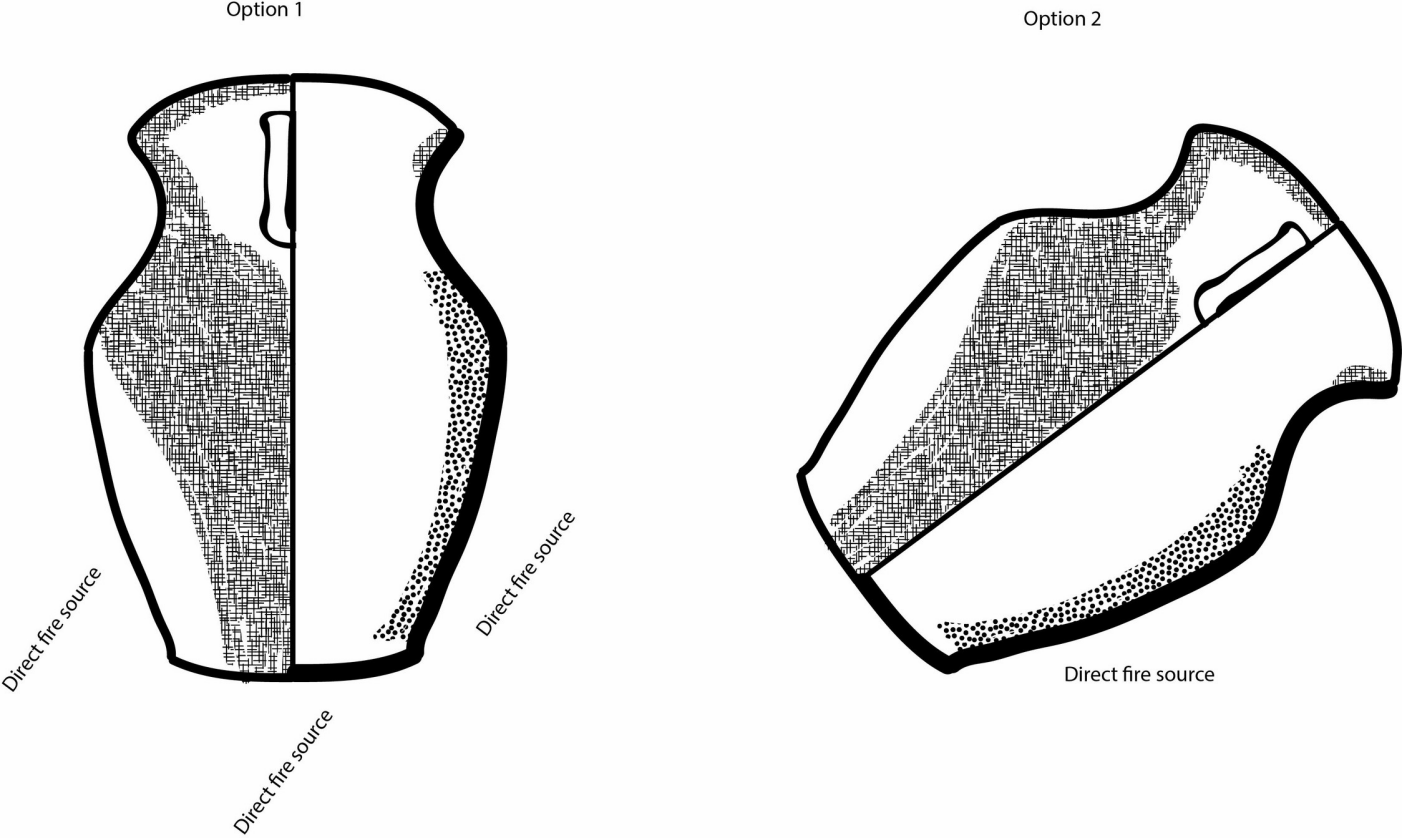
Possible positions that could have generated the dominant use-wear pattern observed on Siwa ma’an cooking vessels. Option 1: commonly understood use position for cooking vessels (it is possible that the vessel was exposed to a heat source on its sides). Option 2: more likely position given the lack of use-wear observed internally on the base of vessels.

The *ma’an* jars, however, exhibit these marks–internally and externally–along the walls (the broad sides of the vessel), while the bases are clean. To obtain this pattern, direct heat would be required where little to no liquid remained, such as roasting or baking, or if pots are placed next to the fire in a “simmer position” exposing only one side to direct heat; as the water evaporated, a charred interior and an external oxidation patch are formed if the pot is left too long [45]. These marks are obtained by quickly evaporating excess liquid in this manner from around the protruding carination (i.e., the part of the vessel closest to the fire source) and not throughout the entire body–as seen on the *ma’an* pots. The intensity of the charring suggests that simmering of cooked grains appears less likely to have been part of the cooking repertoire of *ma’an* pots. Additionally, cooking grains this way begins by placing the vessel in or over the fire source to cook the food, so as to evenly distribute heat, resulting in distinct wet mode use-wear patterns similar to those discussed for the *li* vessels above. This is a possibility, yet due to the higher amount of internal carbonization found throughout the vessel sides, it is more likely that vessels were placed on their side for roasting or frying (Fig 7).

If positioned in this manner, foods cooked on the side placed in the fire/coals would char, resulting in a carbonized patch on the same internal side of the vessel. Externally, an oxidation patch would form in those areas subjected to intense direct heat and where internally little liquid is present, since liquid would otherwise be expected to lower the temperature and prevent the formation of these oxidation patches. Skibo [45] notes that among the Kalinga, some cooking pots (ones thought no longer suitable for boiling) will be used for roasting (coffee, peas, beans, etc.) by placing the pot at a 45-degree angle on supporters.

Locations where food was not placed, i.e., the vessel’s narrow side where the handles were attached, would still be covered with soot on its external side as char, ash and resin droplets would stick to it during the cooking process. For the same reason, the base, internally, would not have accumulated charred food remains because food would mostly be concentrated on its sides and not on the bottom. If placed this way, and used for *dry mod*e cooking, the vessel walls would provide a larger surface on which to cook foods than the rather restricted flat base. Alternatively, it is possible that the *ma’an* pot was placed on its side and food was fried or braised in a small amount of oil or fat, after which liquid could be added to create a stew or soup with the vessel returned to its right side up position and placed next to the fire (as suggested above). The constricted neck would have reduced evaporation in the wet cooking mode as well as retained heat in the dry cooking mode, much like an oven, while reducing moisture loss.

In sum, use-wear analysis reveals that the Siwa *ma’an* pot was quite a sophisticated vessel: it may have been used as a pot, griddle and oven all in one. It is a versatile and portable utensil that can be placed near the fire or on a bed of coals for various dishes, quite possibly where the development of charred but edible food (aka *socarrat* or *fond*) was of potential primary importance. Finally, as this pattern formed on both sides of the vessel it seems that no one side was preferred, and over its lifetime both were used resulting in this vessel-wide use-wear pattern.

This use-wear pattern was found on more than 75% of all Siwa cooking jars from Zhanqi (Table 1), pointing to the prevalence of this cooking style at the site. Even though the vast majority of these data come from the cemetery, thus possibly providing information primarily on mortuary related dishes (either as part of the funeral or as provisions for the afterlife), a majority of *ma’an* cooking vessels unearthed from the limited residential context exhibits this pattern as well.

**Table 1:** Use-wear remains on Zhanqi ceramics.

Context	Dominant cooking use-wear pattern	Indeterminate use-wear pattern	No cooking use-wear/not cooking vessels	N/D
***Graves***	43	13	95	17
***Residential***	6	1	3	1
***Ritual context***	0	2	4	1
***Unknown***	7	1	9	4

**Fig 8:**
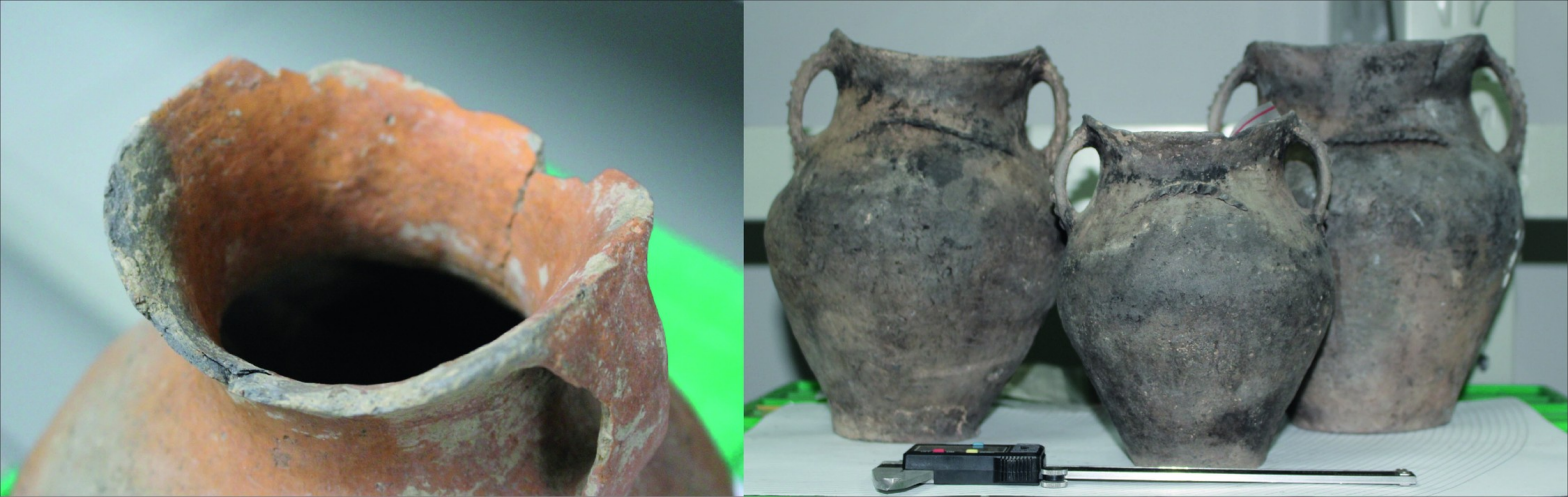
Use-alteration marks (chipping—left) and heavy soot (exposure to fire—right) on *ma’an* vessels.

Most of the burial vessels displayed heavy use alteration marks, such as heavily chipped rims and ‘well-used’ bases (i.e., bases with considerable evidence of scraping, grinding, and other marks of use. See Fig 8 left). This damage accumulated from continued routine use of the ceramic vessel, such as pouring, dragging the pot or even placing it on the floor during its use-life [46]. Vessels used for cooking exhibited use-alteration marks caused by physical force as well and were, additionally, heavily deposited with soot and char, indicating that they must have cooked more than a single meal before they were interred with the dead (Fig 8, right).

## Molecular and isotopic characterization of Ten *Ma’an* Jars

To investigate their contents, carbonized deposits from the interior of ten separate ma’an vessels were analyzed in this study (Table 2) using a variety of complementary analytical approaches (elemental analysis-isotope ratio mass spectrometry–EA-IRMS, gas chromatography-mass spectrometry–GCMS, and compound-specific isotope analysis–GC-c-IRMS; see S1 File). All ten vessels were characterized by the dominant cooking use-wear pattern described in the preceding section, and all of them come from the cemetery area. Nine were associated with well-defined graves while a tenth vessel is from a more ambiguous context within the cemetery, possibly a grave which was not preserved. Unfortunately, no inventory of faunal or botanical remains associated with individual graves is available.

**Table 2:** Results of organic residue analysis of ten pottery sherds from the Zhanqi cemetery in China’s Gansu Province.

Laboratory Code	Context (M = grave)	Lipid conc. (μg mg^-1^)	Lipid composition	APAA(C_18_) E/H	C_16:0_ δ^13^C	C_18:0_ δ^13^C	Δ^13^C	δ^15^N	δ^13^C	C:N
M4-5-F	M4	0.1	FA(C_10-26:0_ C_18:1_ C_15-17br_), DC(C_7-11_), APAA(C_18_), phy(76), pri	3.1	-23.1	-27.9	-4.8	5.4	-16.7	10.6
M16-3-F	M16	0.3	FA(C_10-26:0_ C_18:1_ C_15-17br_), DC(C_7-11_), APAA(C_18_), phy(85), pri, mil	3.4	-23.2	-25.1	-1.9	5.9	-19.8	9.2
M23-3-F	M23	0.2	FA(C_10-26:0_ C_18:1_ C_15-17br_), DC(C_7-11_), APAA(C_18_), phy(85), pri, mil	2.1	-24.5	-29.6	-5.1	5.2	-21.6	10.0
M26-1-F	M26	0.4	FA(C_10-26:0_ C_18:1_ C_15-17br_), DC(C_7-11_), APAA(C_18_), phy(77), pri, mil	4.3	-23.2	-26.6	-3.3	7.0	-14.9	10.5
M30-1-F	M30	0.1	FA(C_10-26:0_ C_18:1_ C_15-17br_), DC(C_7-11_), APAA(C_18_), phy(81), pri, mil	5.0	-23.3	-25.1	-1.9	7.3	-13.9	12.0
M47-3-F	M47	0.1	FA(C_10-26:0_ C_18:1_ C_15-17br_), DC(C_7-11_), APAA(C_18_), phy(84), pri, mil	2.7	-23.7	-27.5	-3.8	6.0	-19.4	9.6
M48-6-F	M48	0.1	FA(C_10-26:0_ C_18:1_ C_15-17br_), DC(C_7-11_), APAA(C_18_), phy(78), pri, mil	3.0	-24.2	-28.4	-4.2	4.9	-18.8	10.1
M51-1-F	M51	0.4	FA(C_10-26:0_ C_18:1_ C_15-17br_), DC(C_7-11_), APAA(C_18_), phy(78), pri, mil	3.6	-24.8	-29.1	-4.3	6.7	-17.2	9.6
M54-7-F	M54	0.1	FA(C_10-26:0_ C_18:1_ C_15-17br_), DC(C_7-11_), APAA(C_18_), TMTD phy(76), pri	3.4	-24.0	-28.5	-4.5	6.6	-17.6	10.1
MZW-F	Unknown cemetery context	0.4	FA(C_10-26:0_ C_18:1_ C_15-17br_), DC(C_7-11_), APAA(C_18_), phy(65), pri, mil	2.1	-25.4	-30.5	-5.1	5.9	-19.4	8.2

FA (Cx:y) = fatty acids with carbon length x and number of unsaturations, br = branched chain acids, DCx = α,ω-dicarboxylic acids with carbon length x, TMTD = 4,8,12- trimethyltridecanoic acid, pri = pristanic acid, phy = phytanic acid, APAA (C18) = ω-(o-alkylphenyl) alkanoic acids with 18 carbon atoms, mil = miliacin. Phy(xx) refers to the ratio of SRR%.

### Results from bulk carbon and nitrogen isotope analysis

**Fig 9:**
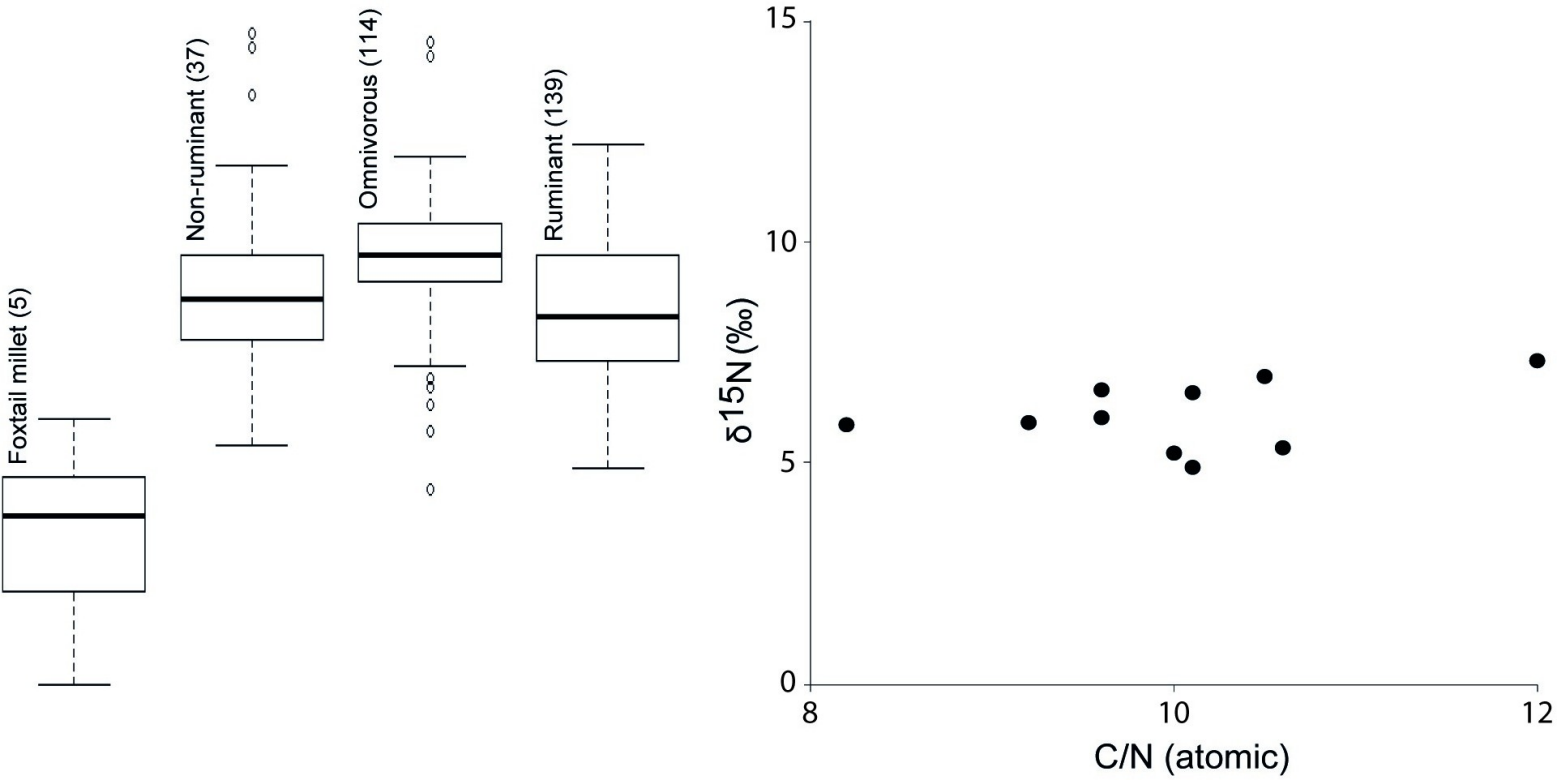
Plot of carbon to nitrogen ratios and δ^15^N obtained on charred deposits (foodcrusts) of Siwa pottery from the Zhanqi site, against archaeological bone collagen reference data from China (see S1 File, Table 1). The collagen δ^15^N values were adjusted by +2‰ to correct for the collagen to tissue offset in order to make these values more comparable with the foodcrusts [51].

Carbonized deposits were analyzed by elemental analysis-isotope ratio mass spectrometry (EA-IRMS) to determine their bulk carbon (δ^13^C) and nitrogen (δ^15^N) stable isotope values, along with carbon-to-nitrogen ratios (C:N). EA-IRMS of charred surface deposits only offers crude resolution of contents because of uncertainties in the isotope end-points of different foodstuffs and diagenetic alteration [47], but when combined with other analytical techniques and compared with reference values it has the potential to provide a number of useful lines of information. The δ^15^N values increase with trophic level and can be used to estimate the proportion of plant and animal protein in terrestrial diets, although variability in δ^15^N values can also be due to a number of external factors, including aridity or soil type. For example, in their study of human skeletal remains from the Chinese province of Gansu, Liu and colleagues [31] interpreted δ^15^N enrichment of bone collagen in post-2000 BC remains as a result of increased aridity rather than animal protein consumption. In this study, δ^15^N values ranging between 4.9‰ and 7.3‰ are consistent with both terrestrial plant and/or animal foods (Fig 9) although these measurements on charred deposits are not directly comparable with bone collagen values. In the absence of marine resources, as is likely the case in Bronze Age Gansu [see 33 for discussion], the degree of enrichment of ^13^C over ^12^C (expressed as δ^13^C) allows the distinction of C_4_ over C_3_ plants or of animals fed C_4_ compared to C_3_ plants. Since millet is by far the most common plant with a C_4_ photosynthetic pathway used for human consumption in Bronze Age China, more positive δ^13^C values have often been used to estimate the shifting importance of this cereal among groups of various cultural and chronological affiliations [4,31,48]. The ten charred deposits included in this study yielded δ^13^C values ranging from -13.9‰ to -21.6‰, indicating variable C_3_ and C_4_ inputs that must include millet or C_4_-fed terrestrial animal products for the more ^13^C enriched values. While millet was the predominant C_4_ component of human food, a range of other grasses and sedges may account for a C_4_ input in the foods of grazing animals. C:N ratios range between 8.17 and 12.04 (Fig 9), suggestive of at least some protein-rich animal products rather than pure millet [49,50]. The atomic C:N ratio is indicative of the amount of protein versus other macromolecules (carbohydrates and lipids). Generally, animal tissues, enriched in protein, have lower C:N ratios compared to plant tissues enriched in carbohydrates such as starch and cellulose, but animal fats would also be expected to have high C:N ratios.

### Results from GCMS analysis of lipids

**Fig 10:**
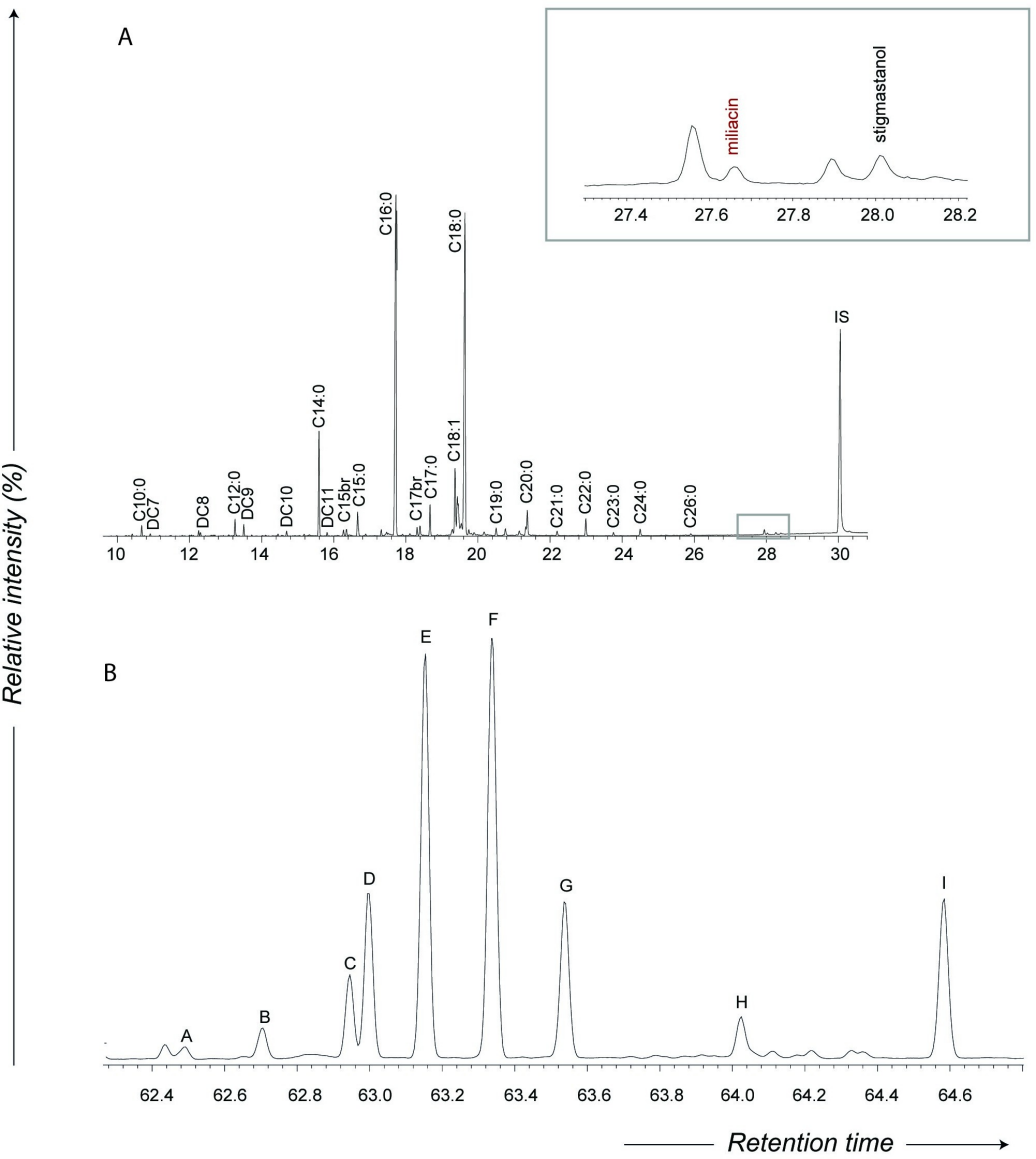
Partial gas chromatograms of lipid extract M51-1-F obtained by direct acid-catalyzed transesterification (for more details see S1 File). A. Partial chromatogram obtained with a DB5-ms (5%-phenyl)-methyl polysiloxane column: Cn:x are fatty acids with carbon length n and number of unsaturations x; br are branched-chain acids; IS are the internal standards (n-tetratriacontane and n-hexatriacontane). The insert shows peaks corresponding to miliacin (olean-18-en-3β-ol methyl ether), a plant biomarker enriched in grains of broomcorn millet (Panicum miliaceum) and stigmastanol, another plant biomarker identified. To monitor the retention time and confirm the presence of miliacin, an authentic standard of miliacin was injected in the same GCMS run. B. Partial SIM chromatogram (m/z 105 ion) obtained with a DB23 (50%-Cyanopropyl)-methylpolysiloxane column shows the distribution of ω-(o-alkylphenyl)alkanoic acids with 18 carbon atoms (letters from A to I corresponding to the isomers).

All of the charred deposits yielded interpretable lipids (> 0.1 ug mg^-1^, [52]), with a mean concentration of 0.2 ug mg^-1^. Analysis of the FAMEs extracted from the *ma’an* jar potsherds shows a high degree of homogeneity in the fatty acid profiles, suggesting that the residues were composed of similar resources (Table 2, Fig 10). Palmitic (C_16:0_) and stearic (C_18:0_) fatty acids are the most abundant components, suggesting a contribution of degraded animal fats to the residues, followed by myristic (C_14:0_) and odd-carbon number branched-chain fatty acids (C_15_ and C_17_). Various food resources contain a notable amount of branched-chain fatty acids, in particular ruminant animals and aquatic resources. The ratio between iso- and anteiso- isomers have been reported to be different, with iso-branched acids much more dominant in aquatic products whereas a roughly equal amount of iso- and antiso- isomers characterize ruminant products [53,54]. In Siwa pottery, the iso:antiso ratio range from 0.74 to 1.16 for C_15br_ (average of 0.96) and from 0.72 to 1.62 for C_17br_ (average of 1.20). While these are close to values expected for ruminant animals, we can’t exclude other sources or a contamination from soil bacteria [55,56].

**Fig 11:**
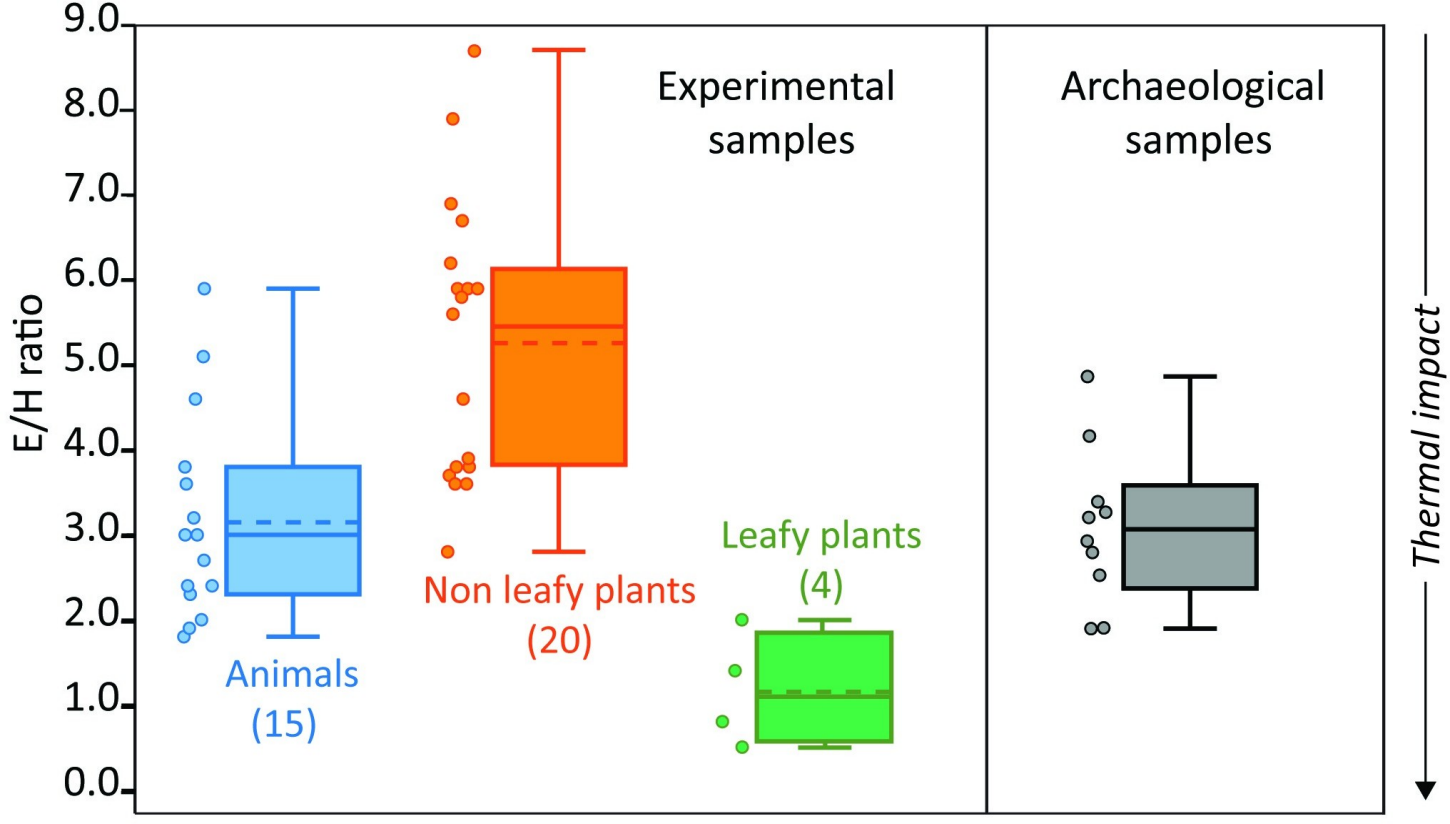
Boxplots of E/H ratio of modern references thermally degraded in the laboratory [57] and Siwa archaeological samples. Plots represent median (solid line), mean (dashed line), ranges and quartiles. The arrow (thermal impact) shows the effect of increasing temperature on the E/H ratio.

Eight samples yielded small amounts of miliacin (olean-18-en-3β-ol methyl ether, *m/z* 189, 204, 231, 425, 440), a plant biomarker enriched in grains of broomcorn millet (*Panicum miliaceum*), along with stigmastanol. A similar set of plant biomarkers has been identified in Bronze Age pottery vessels from the Korean Peninsula and northern Europe and reported as the first molecular and isotopic evidence of millet processing in prehistoric pottery vessels [57]. Another class of compounds identified in all ten acidified methanol extracts are isomers of the ω-(o-alkylphenyl) alkanoic acid (AAPA) with 18 carbon atoms, which are readily formed by heating a wide range of resources rich in unsaturated fatty acids precursors (C_18:x_), notably plant and aquatic resources, but also a number of terrestrial products [58–60]. While previous studies had shown that APAAs were formed when vessels are subjected to temperatures exceeding 270°C for at least 17 hours [58], recent experimental work has shown that these compounds are formed over just one hour of heating at 270°C, or at 200°C for 5 hours, conditions easily achieved through both boiling or roasting [60,61]. Similar experiments also show that the relative abundance of certain APAA-C_18_ isomers (namely the E and H isomers—see Fig 11) can effectively separate three groups of food products: 1) cereals/fruits/non-leafy vegetables, 2) leafy vegetables, and 3) animal/aquatic products [60]. As shown in Fig 11, the E/H ratio associated with the ten Siwa samples (Table 2) reveals a pattern generally more coherent with animal products than plants, although one cannot rule out cereals/non-leafy vegetables like millet as the source of this isomeric distribution.

In addition to AAPA C18 fatty acids, all ten samples yielded trace amounts of two isoprenoid fatty acids (phytanic acid and pristanic acid), and one sample also contained trace amounts of 4,8,12-trimethyltridecanoic acid (TMTD). While TMTD forms mainly in aquatic resources, phytanic and pristanic acids are also found at high concentrations in ruminant carcass tissues and dairy products. The co-occurrence of APAAs with 18 and 20 carbon atoms and isoprenoid fatty acids have been established as reliable indicators for aquatic processing in archaeological ceramics [58,62]. In this case, however, the absence of APAAs with 20 carbon atoms and the lack of evidence for the consumption of fish or mollusks on contemporaneous sites of the Gansu region where zooarchaeological analysis have been done [33,63,64], provide little ground to suggest the presence of aquatic resources. Based on current archaeological evidence and the combination of organic residue data reported here, it is more likely that both the AAPA with 18 carbon atoms and the isoprenoid acids identified in the residues originated from terrestrial products.

The analysis of diastereomers of phytanic acid shows a dominance of the SRR isomer (>75% in 9 out of 10 samples; Table 2), interpreted as typical of an aquatic rather than terrestrial source in archaeological ceramics [65]. However, researchers employing phytanic acid diastereomer ratio to authenticate organic milk and dairy products have demonstrated that variations in the %SRR also occur between ruminant animals based on the proportions of C_3_ vs C_4_ plants in their foddering regime systems [66]. Such variations could be linked to the ways ruminants metabolize C_3_ and C_4_ plants, although Eibler et al. [67] indicate that other factors, such as chlorophyll concentration, are likely involved (with lower chlorophyll content resulting in higher SRR/RRR ratios). Given the dry conditions thought to have characterized the Gansu region during the time period attributed to the Siwa culture (ca. 3350–2650 cal yr B.P), high temperatures and low precipitation may also lead to lower amounts of chlorophyll in pasture. Clearly, further investigation of phytanic acid diastereomers in ruminants with different regime systems and living in different environmental conditions is needed before we can satisfactorily interpret the %SRR observed in this study.

### Results from compound specific isotope analysis

**Fig 12:**
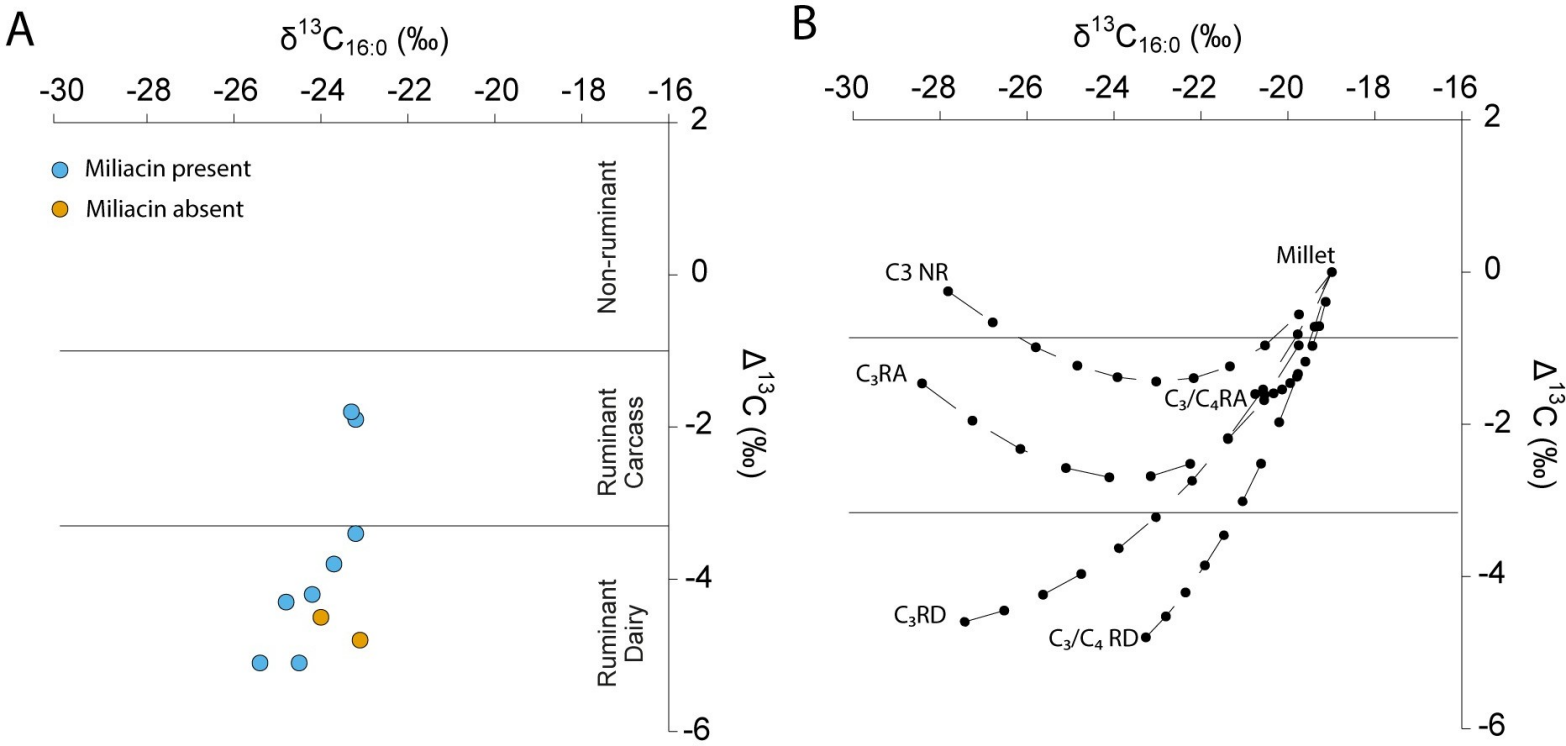
Compound specific stable carbon isotopic data from Siwa potsherds compared with reference fats and oils and illustration of potential mixtures. In each plot the δ^13^C_16:0_ values are plotted against Δ^13^C (δ^13^C_18:0_ - δ^13^C_16:0_) to distinguish the different sources of fats. A. Fatty acids carbon stable isotope values plotted against reference ranges. The ranges represent the mean ± 1 s.d. of the Δ^13^C values for a global database comprising modern reference animal fats raised on a variety of C-3 and mixed C3/C4 diets [69] B. Shows average isotopic endpoints for non-ruminant (NR), ruminant adipose (RA), and ruminant dairy (RD) fats with C_3_ and mixed C_3_/C_4_ diets and millet obtained from measurements of authentic modern products corrected for post-industrial carbon (S1 File, Table 2). Hypothetical mixing lines in 10% increments are shown between each endpoint and millet calculated from the mean relative amount of each fatty acids in each product using data obtained from the USDA database.

GC-c-IRMS analyses were carried out on the ten surface residues from the Zhanqi cemetery *ma’an* jars to determine the δ^13^C values of the major *n*-alkanoic acids, palmitic (C_16:0_) and stearic (C_18:0_), and thereby provide further information on the source of extracted fats. It has previously been established that C_18:0_ fatty acids in ruminant animals are depleted in ^13^C compared to C_16:0_ acids [68]. Differential routing of dietary carbon during the biosynthesis of milk versus meat fats in ruminant animals further allows dairy and adipose fatty acids to be distinguished [69]. Typically, C_18:0_ fatty acids in ruminant adipose fats are depleted in ^13^C by 1–3.1‰ compared to C_16:0_ fatty acids while ruminant dairy fats display Δ^13^C values of less than − 3.1‰ [70–72]. The ten residue samples analyzed in this study yielded δ^13^C values for palmitic (C_16:0_) fatty acids ranging from − 23.1 to– 25.4‰, while the δ^13^C values associated with stearic (C_18:0_) fatty acids range from– 25.1 to– 30.5‰ (Table 2, Fig 12A). Eight of the ten food-crust samples from the Zhanqi site plot within the ruminant dairy range, with Δ^13^C values ranging from– 3.3 and– 5.1‰. The Δ^13^C values of the two remaining *ma’an* jar residues are both– 1.9‰, firmly ranging within the ruminant adipose range (Fig 12A).

However, rather than a single source, the molecular and isotopic characterization of *ma’an* jar residues indicate a complex distribution of lipids from C_4_ plants (i.e., millet) and ruminant adipose and/or dairy fats. Given such a contribution from multiple food sources, carbon isotope measurements of individual fatty acids (C_16:0_ and C_18:0_) cannot simply be matched with reference data for straightforward source identification. Recently, the use of Bayesian mixing models have been advocated to better understand the proportional contribution of different food sources, but to be trusted these must rely on a range of reference data representative of the environmental and cultural contexts under investigation [73]. In the absence of such a comparative baseline for the Zhanqi site, this study uses simple linear mixing models based on compound specific measurements from published sources, each one involving the mixing of millet with a different source of animal fat: non-ruminant, ruminant adipose and ruminant dairy. Because foods eaten by animals exhibit characteristic isotopic signatures, the δ^13^C values of fatty acids extracted from archaeological potsherds are also a reflection of these animals’ consumed diet [68,74,75]. The animal reference values selected for this study are thereby classified to distinguish between ruminant and non-ruminant species, adipose and dairy fats, and C_3_- and mixed C_3_/C_4_-fed animals (S1 File, Table 2). Fig 12B indicates that the mixing of millet and dairy is the most likely explanation for the δ^13^C measurements of palmitic and stearic fatty acids (C_16:0_ and C_18:0_) obtained on eight of the ten Siwa samples. These values also best fit the mixed C3/C4 ruminant diet, supporting bulk carbon and nitrogen measurements obtained from archaeological animal bones at various contemporaneous sites in Gansu (S1 File, Table 1). In line with previous studies, these values point to a mixed C_3_ and C_4_ diet for ruminant animals, unlike pigs and dogs which appear to be fed mainly with C_4_ plants. The last two Siwa samples appear as outliers and are harder to interpret. They could represent the mixing of millet and C_3_ fed non-ruminant animals, although a complex mixture of other fat sources cannot be ruled out at this point.

## Residue and use-wear analysis in the context of food and society at Zhanqi

In recent years a growing number of stable isotope studies have argued for a shift in northwestern China from predominately C_4_ foods to a mixed C_3_ and C_4_ diet during the 2^nd^ millennium BCE [4,28–31,76,77]. While most of these studies have focused on the Hexi corridor, situated to the north and west of known Siwa site locations, several have in fact looked at human skeletal evidence from the region, mostly from earlier periods, but also from Zhanqi itself. At Zhanqi, Liu et al. [31] found an average marker depleted in ^13^C, which they interpreted as a mix of C_4_ and C_3_ plants (and using archeaobotanical comparative samples from elsewhere). Millet, taken to be the main C_4_ crop, is assumed to have been directly consumed, while C_3_ foods (barley and wheat) were obtained via animals grazed on wild grasses, though, when compared to earlier period sites, the level of C_4_ plants seems rather depleted. High δ^15^N values are taken to indicate higher levels of meat consumption (animals or fish) overall, especially in comparison with other contemporary and earlier period communities. Cheung et al. [50] too, have argued for a drop in C_4_ plant contribution to Zhanqi diets, but suggest that in-site variability among individuals was high in comparison to many other sites.

Data reported here indicate the continued importance of millet, both as animal fodder but also for direct human consumption. Preparation of millet (a C_4_ plant) in *ma’an* jars for human consumption is attested on the basis of the miliacin molecular compound present in a majority of chromatographic profiles. Because millet is a C_4_ plant, its contribution to the residues was further supported by bulk carbon isotope values, although in this case it is impossible to say whether the C_4_ signal results from millet used for human consumption or from animals fed on C_4_ plants. In addition to the continued importance of millet following the warm-humid Holocene climatic optimum in the Loess Plateau, this study suggests the use of ruminant dairy products at the Zhanqi site. The pivotal period comprised between 3350 and 2650 cal yr B.P. has been associated with the migration of nomadic communities and the adoption of pastoralism as an important mode of subsistence. Biomolecular approaches have shown that initial animal domestication and the subsequent spread of both animal and herding practices are often closely linked with the adoption of dairy into adult diets [78–81]. In a recent contribution, Wilkin and colleagues analyzed ancient proteins from human dental calculus and demonstrated that ruminant dairy consumption occurred concomitantly with the initial pastoral occupation of Mongolia, about 5000 years ago [82]. Within the PRC, the earliest direct evidence for milk consumption comes from the Tarim Basin of the Xinjiang region, where milk proteins have been identified in a Middle Bronze Age woven basket [83] and in pieces of a circa 3800-year old well-preserved kefir cheese [84]. The presence of dairy fats in a portion of the *ma’an* jars recovered from the Zhanqi site would provide the easternmost early evidence for dairying practices within the area of modern China, among pastoral communities who are attributed to the Siwa archaeological culture who occupied the Loess Plateau between ca. 3350 and 2650 cal yr B.P.

It is worth noting that without the miliacin biomarker, molecular data obtained from Siwa foodcrusts could have been interpreted as originating mainly, if not exclusively, from degraded animal fats, with the contribution of plants to human diet going completely unnoticed. We must thereby exercise caution when interpreting very negative Δ^13^C values as evidence for dairy given the difficulty of identifying C_4_ plant-animal mixes. Past experiments have shown that when mixtures of meat and plants are involved, the meat signal tends to overwhelmingly dominate, and often completely obliterate, the plant signal [57,85,86]. As a matter of fact, the present study cannot speak about the possible contribution of C_3_ plants (such as wheat, barley, or even rice) to the residues. Unless they represent the sole or dominant contributor to a residue, plants without associated biomarker easily go undetected, their molecular traces masked by lipid-rich resources such as terrestrial animals and aquatic resources. Unlike C_4_ plants, bulk and single-compound isotope signals from C_3_ plants are difficult to distinguish from non-ruminant animals and/or freshwater resources, further complicating their identification in residues. In order to better assess the contribution of C_3_ plants to residues, lipid analysis of charred deposits should, when possible, be combined with microfossil analysis of starch and phytoliths. Future research in the constantly evolving field of organic residue analysis will also undoubtedly lead to the identification of new plant biomarkers, as recent studies demonstrate [87].

Adding to this evidence, our use-wear alteration study shows the versatility of the *ma’an* ceramic pot. The observed patterns (Fig 4) could have been made by using the vessels sides to maximize contact area while maintaining high heat efficiency. Combined with the above residue analysis results, which found a mix of millet, ruminant dairy, and possibly other sources, we might envision what was cooked in these vessels. As direct archaeological evidence for how ingredients were used is scarce, gaining insights from both ethnographic sources, such as observations recorded from different pastoral societies (including later more nomadic ones), alongside historical scholarship is useful (see discussion in [88]).

Even as early Chinese texts report that their pastoralist neighbors consumed a diet centered around meat, the finding of ruminant dairy fats in these pots is not surprising. Many pastoral societies consumed greater quantities of milk and dairy products than meat (overview in [89]). As noted above, in areas of Eurasia further north and west of the Siwa cultural region, evidence for dairy products has been found dating back to the early 4^th^ millennium BP [83,84]. Indeed, for the Mongols, dairy (milk and cheese) accounted for a large amount of daily caloric intake, mostly in winter, and fermented mare’s milk provided a sustaining drink year-round [90]. There is also an increasing appreciation of the centrality of grains, even among those consuming a largely pastoral diet [91–93]. In medieval Mongolian diets, only rarely was meat roasted on spits over open fires [90]; in daily meals, meat was boiled in water and grains were added to the traditional thick stews [94]. Elsewhere, the Samburu of Kenya occasionally roast meat, but boiling meat, with grains, blood and milk is often preferred [95]. For Bedouin communities in Jordan, daily meals are based on mixtures of dairy and grain products. A wide range of foods mixing wheat flour and dairy products are known, including: porridges, dumplings, breads and simple mixing of groats cooked with milk or yogurt and then dried [96]. Nomadic pastoralists of Tibet often consume a single stew of animal fat (mostly dairy but also meat in winter) with boiled dumplings [97] and Turkic nomadic cuisine had a propensity for dairy in everyday meals combined with bread or porridges made from millet or wheat [98].

For the pastoralists of Sudan [99] millet kernels are boiled with or without milk or added to stews. Often millet is left to ferment in water after which it is ground into a flour or course meal. These can be used to make (mostly) a millet like porridge consumed daily by simply cooking millet flour in water. Millet flour can be combined with milk or other liquid to make simple bread usually in embers, or on a flat metal slab as a fine layer like a crepe or dosa. Toasting grains with dairy products is common among pastoralists as well. A favored dish among the Kazakhs is a mix of milk cooked with butter and toasted millet [98]. Tibetian *tsamba–*barley that is roasted–is eaten in the morning, mixed with tea and butter, and supplemented with dairy products such as yogurt and cheese [100,101].

Use-wear alteration analysis indicates that meat could have been roasted with or without millet grains and possibly made into thick stews by frying before placing the vessel back next to the fire right side up and adding liquid. Additionally, it is not unreasonable to posit that millet grains were toasted in the *ma’an* pots. Butter, cheese or milk could have been added to the mix during or after the toasting process. Yet, while dairy remains were found in *ma’an* pots, using them to process milk into cheese or butter seems less likely. Since fresh milk spoils quickly and milking is a seasonal resource, preservation is a primary concern for pastoral societies [102]. Milk can be processed into a range of dairy products, with or without the application of heat, where ceramic vessels would be quite useful [103]. For yogurt, fresh milk can be boiled or left out and kept warm to allow for bacteria (often artificially introduced) to ferment. The fermented (or soured) milk and yogurt can then be churned to separate butter (fats) and buttermilk (i.e. the liquid remains of the churning process)–the latter further processed to create cheeses and other dairy products [96]. Processing of buttermilk involves heating to separate the curds from the whey. In traditional pastoral societies this typically is done in a pot over a low flame for several minutes [102]. To make clarified butter–butter where milk solids and water have been removed–butter is heated at low temperatures.

The heating of milk in the above-mentioned processes would produce a wet type of use-wear alteration pattern, such as a ring above the liquid line–a pattern not seen in the *ma’an* pots. Churning would be difficult with the *ma’an* pots as with any other known ceramic vessels types, though skins could have been used of course. Clarified butter making is not supported either, since solids form on the base of the vessel if used upright, and if placed on the side the heated liquid will spill. Additionally, as evaporation of liquid is desired, the constricted opening would be ill-suited for the job and in fact raise the temperature of the butter leading to burning. An open, wide mouthed pot or pan would be better suited for the task [104].

In contrast, it is quite likely that grains were processed–ground into flour or broken up before use–as evidenced by grinding stones found at Ya’ar, where some of the only residential remains of the Siwa culture have been excavated [26]. Indeed, use-wear alteration remains do hint at the possibility of a flat bread or cake cooked on the vessel’s side. If frying or roasting was desired, why not use a pan or a simple flat ceramic sheet–as is documented for example in the Amazon [105]? One possibility is that unlike an open griddle or pan, a closed pot would retain heat (similar in shape to a tandoori oven but heated externally), allowing for less fuel to be used. It would additionally retain moisture during the cooking process, which would have been desirable for certain grain and meat-based dishes. Furthermore, vessels could be preheated and then taken out of the fire source to roast or cook food nearby without losing the gained heat. Unlike broiling, toasting, browning or even baking need not be done directly in the fire, as exposing food from a distance can achieve this as well [106].

As noted above, the vessels display a number of use markers indicating that they most probably cooked more than a single meal before they were interred with the dead. Indeed, it is important to point out that both residue and use-wear analyses provide the cumulative use and cooking instances of a pot (though when food crusts are analyzed this is less likely). As such, it is possible that the *ma’an* pot was used in a number of ways. Work by Rouse et al. [107] reminds us of the importance of re-examining preconceived notions of vessels function–especially among what has been perceived as pastoral nomadic groups. Experiments with food processing and cooking techniques are becoming more important in recent years as a way to gain insight into ancient cuisines and cooking techniques [88,108–115]. Controlled experimentation in the NYU culinary school kitchens showed that the use-wear patterns observed on the *ma’an* jars could be created via the two aforementioned cooking methods, i.e. by placing a vessel on its side in a fire to roast foods and/or by placing it upright in the fire and adding liquid after initial browning on its side was achieved. To better test our models regarding how these unique patterns would have formed in the *ma’an* vessels, we conducted a series of experiments with millet and meat cooked with varying degrees of heat sources and intensities. The results support our model where observed *ma’an* use-wear pattern could have been generated by baking, roasting and cooking in this above-mentioned manner (see S2 File).

## Conclusion

Foodways have played a central role in the way ‘The Other’ in Bronze Age China has been presented. Scholars view cultures inhabiting the Western fringes of the Shang and Zhou world as those eating a diet rich in meat and low in grain befitting a nomadic pastoral peoples. This study is the first of its kind to be conducted on pottery from Bronze Age China. The combination of use-wear and lipid analysis provides a glimpse into how food was actually prepared and consumed by ancient communities. Here we report molecular data suggesting the preparation of meals composed of millet grain and ruminant dairy fats among the Siwa community of Zhanqi. These finds nuance the centrality of meat in the Siwa period diet and bring further support to the aforementioned isotope studies at Zhanqi [31,48]. Furthermore, use-wear alteration analysis showed that Zhanqi community members were sophisticated creators of ceramic equipment, the *ma’an* cooking pot, which allowed them to prepare a wide number of dishes with limited fuel.

The study of foodways has been a welcome addition to an ever-growing body of analytical methods on past societies. Even as the Siwa archaeological culture is known mainly from graves and great variation existed in burial practices (from cremation to inhumation, of singular and shared graves as well as secondary graves), the study of foodways reflects another avenue of research that could highlight differences as well as commonalities among the people inhabiting Bronze Age Gansu. It further allows us to reflect on local-specific manifestations of social identity that go beyond simplistic overarching frameworks that homogenize all groups employing similar ceramic styles [46,88,116–118].

Use-wear alteration and residue analysis greatly expand the arsenal of analytical methods geared towards understanding ancient dietary habits and foodways, but they should not be seen as methods providing conclusive and all-inclusive information on diet. Such results complement the results of stable isotope studies and contextual archaeobotanical and zooarchaeological data from properly excavated and analyzed contexts, more of which are urgently needed to better evaluate and hone the validity and resolution of these methods (and see [22]). If a more definite interpretation of the extent and nature of resource mixing in *ma’an* vessels must await future research, the internal consistency of the residue results and the homogeneity of use-wear patterns at the Zhanqi cemetery allow us to build strong hypotheses about foodways at this Siwa culture site and beyond.

## Supporting information

S1 File (DOCX)

S2 File (DOCX)
