# Supplementary material for: What do “barbarians” eat? Integrating ceramic use-wear and residue analysis in the study of food and society at the margins of Bronze Age China
Source: PLoS One. 2021 Apr 29;16(4):e0250819. doi: 10.1371/journal.pone.0250819 (PMC8084173; doi:10.1371/journal.pone.0250819)
Supplement: S1 File — (DOCX) [file pone.0250819.s001.docx]

*Supplementary Methods*

For elemental analysis-isotope ratio mass spectrometry (EA-IRMS), no pre-treatment of the samples was undertaken prior to analysis (for discussion see Craig et al. 2007 and Morton and Schwartz 2004). Each sample was weighed in duplicate (*c.*700ug) into tin capsules, which were analyzed using a Flash 2000 Organic Elemental Analyzer linked to a Delta V Plus Isotope Ratio Mass Spectrometer (both from Thermo Scientific). δ^13^C and δ^15^N measurements were determined relative to the VPDB and AIR international standards, respectively. Carbon and nitrogen isotopes were analyzed in the same analytical run. Samples yielding less than 1% N were discarded and instrument precision on repeated measurements was always better than 1‰, (and most of the time better than 0.5‰) for both elements, as determined by duplicate measurements.

## For lipid analysis by gas chromatography-mass spectrometry, charred deposits (50-200 mg scraped from the potsherd interior surface) were weighed and lipids were extracted and methylated according to established protocol by direct acid-catalyzed transesterification to maximize recovery (Correa-Ascencio and Evershed 2014). Methanol (1 mL) was added and homogenized with the charred deposit, the mixture was ultrasonicated for 15 min and then acidified with concentrated sulphuric acid (200μL). The acidified suspension was heated in sealed tubes for 4 h at 70 °C and then cooled, and lipids were extracted with n-hexane (3 × 2 mL) and dried down under a gentle N2 flow. The extracts were transferred to autosampler vials and 10μg of internal standard (hexatriacontane) was added. Lipids extracted from charred deposits were analyzed by GCMS, a technique that allows the separation of complex mixtures and the identification of plant- and animal-derived lipids (e.g., sterols, n-alkanoic acids). GCMS analysis was performed using an Agilent 7890A Series gas chromatograph connected to an Agilent 5975 C Inert XL mass-selective detector with a quadrupole mass analyzer (Agilent Technologies, Cheadle, Cheshire, UK). The splitless injector and interface were maintained at 300 °C and 280 °C respectively. Helium was the carrier gas at constant inlet pressure, and the GC column was inserted directly into the ion source of the mass spectrometer. The ionization energy was70 eV and spectra were obtained by scanning between m/z50 and 800. All samples were analyzed using a DB5-ms (5%-phenyl)-methyl polysiloxane column (30 m × 0.32 mm × 0.25μm; J&W Scientific, Folsom, CA, USA). The temperature program was 2 min at 50 °C, 10 °C min−1to325 °C and 15 min at 325 °C. In addition to the above general scanning, a SIM method was performed to identify specific biomarkers. The column used was DB23 (50%-Cyanopropyl)-methylpolysiloxane column (60m, 250μm & 0.25μm; J & Scientific, Folsom, CA, USA). The temperature program was 2 minutes at 50°C, 10°C/min to 100°C, 4 °C/min to 140 °C, 0.5°C/min to 160°C and 20°C /min to 250°C for 10 minutes. Helium was also used as the carrier gas at a rate flow of 1.5mL/min (Shoda et al. 2017). Four groups of ions were selected: m/z 74, 87, 213, 270 to identify 4,8,12- trimethyltridecanoic acid ; m/z 74, 88, 101, 312 for pristanic; m/z 74, 101, 171, 326 for phytanic and m/z 74, 105, 262, 290, 318, 346 for ω-(o-alkylphenyl) alkanoic acids corresponding to the carbon length C_16_ to C_22_, respectively. This method was also conducted to confirm the source of lipids by calculating the relative contribution of AAPA-C_18_ isomers (Bondetti et al. in press) and phytanic’s SRR diastereomer (Lucquin et al. 2016a).

## To better characterize the lipids extracted from *ma’an* jars, GC-c-IRMS analysis of the Zhanqi samples was conducted in order to ascertain the 13C/12C ratio in the two most abundant fatty acids, i.e., octadecanoic (C_18:0_) and hexadecanoic (C_16:0_). In addition, the 13C/12C ratios of octadecanoic (C_18:0_) and hexadecanoic (C_16:0_), extracted from modern *P. miliaceum* seeds, were analysed for comparison with archaeological residues. The samples were analyzed using an Agilent 78,908 GC (Agilent Technologies. Santa Clara. CA. USA) instrument coupled to an Agilent 5975C MSD and an Isoprime 100 IRMS (Isoprime, Cheadle, UK) with an Isoprime GC5 interface (lsoprime, Cheadle, UK). All samples were diluted with hexane and subsequently 1μL of each sample was injected into a DB-5MS (30 m × 0.25 mm × 0.25μm) fused-silica column. The temperature was set for 0.5 min at 50 °C, and raised by 10 °C min−1until 300 °C was reached, and held for 10 min. The carrier gas was ultra-high purity grade helium with a flow rate of3 mL min−1. The gases eluting from the chromatographic column were split into two streams. One of these was directed into an Agilent 5975Cinert mass spectrometer detector (MSD), for sample identification and quantification, while the other was directed through the GC5 furnace held at 850 °C to oxidize all carbon species to CO2. A clear resolution and baseline separation of the analyzed peaks was achieved in both systems. Eluted products were ionized in the mass spectrometer by electron impact. Ion intensities of m/z44, 45, and 46 were monitored in order automatically to compute the13C/12C ratio of each peak in the extracts. Computations were made with Ion Vantage and IonOS Softwares (Isoprime, Cheadle, UK) and were based on comparisons with a standard reference gas (CO2) of known isotopic composition that was repeatedly measured. The results from the analysis are reported in parts per mille (‰) relative to an international standard (V-PDB). The accuracy and precision of the instrument was determined on n-alkanoic acid ester standards of known isotopic composition (Indiana standard F8–3). Archaeological and reference samples were measured in replicate, with archaeological samples having a mean S.D. of 0.06‰ for C16:0 and 0.04‰ for C18:0 and reference samples a mean S.D. of 0.17‰ for C16:0 and 0.15‰ for C18:0. Values were also corrected subsequent to analysis to account for the methylation of the carboxyl group that occurs during extraction. Corrections were based on comparisons with a standard mixture of C16:0 and C18:0 fatty acids of known isotopic composition processed in each batch under identical conditions. In addition, reference samples were corrected (-2.2‰) to account for the Suess effect.

*Supplementary Table 1.* *Bulk carbon and nitrogen isotope values of reference material from China used in this study*

| **Category** | **Common name** | **Taxa** | **Sample type** | **Period** | **Provenience** | **C13(‰)** | **N15(‰)** | **C/N** | **Reference** |
| --- | --- | --- | --- | --- | --- | --- | --- | --- | --- |
| C4 plants | Foxtail millet | *Setaria italica* | charred grains | Late Yangshao | Chinese Loess Plateau | −10.4 | 3.8 |  | An et al. 2015 |
| C4 plants | Foxtail millet | *Setaria italica* | charred grains | Majiayao | Chinese Loess Plateau | −10.1 | 6.0 |  | An et al. 2015 |
| C4 plants | Foxtail millet | *Setaria italica* | charred grains | Qijia | Chinese Loess Plateau | −9.4 | 4.7 |  | An et al. 2015 |
| C4 plants | Foxtail millet | *Setaria italica* | charred grains | Modern | Chinese Loess Plateau | −12.3 | 2.1 |  | An et al. 2015 |
| C4 plants | Foxtail millet | *Setaria italica* | leaves | Modern | Chinese Loess Plateau | −12.6 | 0.0 |  | An et al. 2015 |
| Ruminant adipose |  | Bos | Bone | Bronze Age | Qiajiaping (Gansu) | -16.2 | 8.3 |  | this study |
| Ruminant adipose | Cattle |  | Bone | Bronze Age | Huizuiwa (Gansu) | -16.8 | 6.9 |  | this study |
| Ruminant adipose | Cattle |  | Bone | Bronze Age | Huizuiwa (Gansu) | -17.1 | 7.9 |  | this study |
| Ruminant adipose | Large Bovine |  | Bone | Bronze Age | Dayatou (Gansu) | -14.8 | 7.9 | 3 | this study |
| Ruminant adipose | Large Bovine |  | Bone | Bronze Age | Dayatou (Gansu) | -17.2 | 7.9 | 2.9 | this study |
| Ruminant adipose | Large Bovine |  | Bone | Bronze Age | Dayatou (Gansu) | -17.0 | 6.2 | 2.9 | this study |
| Ruminant adipose | Large Bovine |  | Bone | Bronze Age | Huizuiwa (Gansu) | -21.2 | 8.9 | 2.9 | this study |
| Ruminant adipose | Large Bovine |  | Bone | Bronze Age | Qijiaping (Gansu) | -17.6 | 10.0 | 3.2 | this study |
| Ruminant adipose | Large Bovine |  | Bone | Bronze Age | Qijiaping (Gansu) | -17.0 | 7.7 | 3.1 | this study |
| Ruminant adipose |  | Capra | Bone | Bronze Age | Huizuiwa (Gansu) | -15.3 | 6.4 |  | this study |
| Ruminant adipose |  | Capra | Bone | Bronze Age | Huizuiwa (Gansu) | -16.4 | 9.6 |  | this study |
| Ruminant adipose |  | Caprinae | Bone | Bronze Age | Huizuiwa (Gansu) | -16.6 | 7.5 |  | this study |
| Ruminant adipose |  | Caprinae | Bone | Bronze Age | Qijiaping (Gansu) | -18.7 | 5.8 |  | this study |
| Ruminant adipose |  | Caprinae | Bone | Bronze Age | Qijiaping (Gansu) | -19.2 | 4.6 |  | this study |
| Ruminant adipose |  | Caprinae | Bone | Bronze Age | Qijiaping (Gansu) | -18.1 | 5.2 |  | this study |
| Ruminant adipose |  | Caprinae | Bone | Bronze Age | Qijiaping (Gansu) | -17.7 | 7.8 |  | this study |
| Ruminant adipose |  | Caprinae | Bone | Bronze Age | Dayatou (Gansu) | -16.0 | 7.1 |  | this study |
| Ruminant adipose |  | Caprinae (Capra?) | Bone | Bronze Age | Dayatou (Gansu) | -17.6 | 8.0 |  | this study |
| Ruminant adipose |  | Caprinae | Bone | Bronze Age | Dayatou (Gansu) | -17.0 | 5.3 |  | this study |
| Ruminant adipose |  | Capra | Bone | Bronze Age | Qijiaping (Gansu) | -18.2 | 4.6 |  | this study |
| Ruminant adipose |  | Capra | Bone | Bronze Age | Qijiaping (Gansu) | -18.4 | 4.8 |  | this study |
| Ruminant adipose |  | Capra | Bone | Bronze Age | Qijiaping (Gansu) | -17.9 | 3.8 |  | this study |
| Ruminant adipose |  | Caprinae | Bone | Bronze Age | Qijiaping (Gansu) | -17.7 | 4.6 |  | this study |
| Ruminant adipose |  | Capra hircus | Bone | Bronze Age | Huizuiwa (Gansu) | -15.5 | 7.1 | 2.9 | this study |
| Ruminant adipose |  | Capra hircus | Bone | Bronze Age | Huizuiwa (Gansu) | -8.2 | 6.3 | 2.8 | this study |
| Ruminant adipose |  | Ovis/Capra | Bone | Bronze Age | Huizuiwa (Gansu) | -9.3 | 5.9 | 2.8 | this study |
| Ruminant adipose |  | Ovis/Capra | Bone | Bronze Age | Qijiaping (Gansu) | -16.7 | 6.9 | 3.3 | this study |
| Ruminant adipose |  | Ovis | Bone | Bronze Age | Huizuiwa (Gansu) | -16.6 | 7.7 |  | this study |
| Ruminant adipose |  | Ovis | Bone | Bronze Age | Dayatou (Gansu) | -15.8 | 6.0 |  | this study |
| Ruminant adipose |  | Ovis aries | Bone | Bronze Age | Huizuiwa (Gansu) | -15.5 | 6.5 | 2.9 | this study |
| Ruminant adipose |  | Ovis aries | Bone | Bronze Age | Huizuiwa (Gansu) | -16.6 | 6.6 | 3.1 | this study |
| Ruminant adipose | deer |  | Bone | Bronze Age | Qijiaping (Gansu) | -19.6 | 3.7 |  | this study |
| Ruminant adipose | roe deer |  | Bone | Bronze Age | Qijiaping (Gansu,) | -18.4 | 7.6 |  | this study |
| Ruminant adipose |  | Medium Cervid? | Bone | Bronze Age | Huizuiwa (Gansu) | -17.2 | 6.1 | 2.0 | this study |
| Ruminant adipose |  | Medium Cervid? | Bone | Bronze Age | Qijiaping (Gansu) | -17.4 | 5.4 | 3.2 | this study |
| Ruminant adipose |  | Medium Cervid? | Bone | Bronze Age | Qijiaping (Gansu) | -17.2 | 6.0 | 3.2 | this study |
| Ruminant adipose | deer | Moschus | Bone | LBP | Dadiwan (NW China) | -20.8 | 5.8 | 2.8 | Barton et al. 2009 |
| Ruminant adipose | deer | Moschus | Bone | LBP | Dadiwan (NW China) | -20.9 | 4.6 | 2.8 | Barton et al. 2009 |
| Ruminant adipose | deer | Moschus | Bone | LBP | Dadiwan (NW China) | -21.1 | 6.9 | 2.8 | Barton et al. 2009 |
| Ruminant adipose | deer | Moschus | Bone | LBP | Dadiwan (NW China) | -20.2 | 5.6 | 2.7 | Barton et al. 2009 |
| Ruminant adipose |  | Bos | Bone | LBP | Dadiwan (NW China) | -19.9 | 8.0 | 3.1 | Barton et al. 2009 |
| Ruminant adipose | deer | Cervus | Bone | LBP | Dadiwan (NW China) | -21.0 | 6.4 | 2.7 | Barton et al. 2009 |
| Ruminant adipose | deer | Cervus | Bone | LBP | Dadiwan (NW China) | -21.0 | 7.8 | 2.7 | Barton et al. 2009 |
| Ruminant adipose |  | Bos | Bone | LBP | Dadiwan (NW China) | -22.1 | 8.1 | 2.7 | Barton et al. 2009 |
| Ruminant adipose | deer | Cervus | Bone | DDW | Dadiwan (NW China) | -20.2 | 7.1 | 2.8 | Barton et al. 2009 |
| Ruminant adipose | deer | Cervus | Bone | DDW | Dadiwan (NW China) | -19.4 | 6.2 | 2.8 | Barton et al. 2009 |
| Ruminant adipose | deer | Cervus | Bone | LBP | Dadiwan (NW China) | -18.9 | 5.6 | 2.8 | Barton et al. 2009 |
| Ruminant adipose | deer | Cervus | Bone | DDW | Dadiwan (NW China) | -20.0 | 8.1 | 2.7 | Barton et al. 2009 |
| Ruminant adipose | deer | Cervus | Bone | DDW | Dadiwan (NW China) | -20.7 | 7.3 | 2.7 | Barton et al. 2009 |
| Ruminant adipose |  | Ovicaprid | Tibia | Machang ( | Xiahaishi (Loess Plateau) | -15.6 | 7.7 | 3.2 | Ma et al. 2014 |
| Ruminant adipose |  | Ovicaprid | Humerus | Machang ( | Xiahaishi (Loess Plateau) | -12.1 | 7.3 | 3.2 | Ma et al. 2014 |
| Ruminant adipose |  | Bos sp. | Humerus | Machang ( | Xiahaishi (Loess Plateau) | -19.5 | 7.6 | 3.2 | Ma et al. 2014 |
| Ruminant adipose |  | Large bovid | Tibia | Machang ( | Xiahaishi (Loess Plateau) | -19.4 | 7.2 | 3.2 | Ma et al. 2014 |
| Ruminant adipose |  | Cervidae | Pelvis | Machang ( | Xiahaishi (Loess Plateau) | -21.1 | 4.4 | 3.2 | Ma et al. 2014 |
| Ruminant adipose |  | Bos sp. | Phalanx III | Machang ( | Xiahaishi (Loess Plateau) | -22.5 | 2.9 | 3.2 | Ma et al. 2014 |
| Ruminant adipose |  | Large bovid | Calcaneum | Machang ( | Xiahaishi (Loess Plateau) | -22.3 | 3.2 | 3.2 | Ma et al. 2014 |
| Ruminant adipose |  | Bos sp. | Mandible | Machang ( | Xiahaishi (Loess Plateau) | -15.1 | 5.7 | 3.2 | Ma et al. 2014 |
| Ruminant adipose | Cattle |  | Skull | Qijia | Qijiaping (Gansu, China) | -16.1 | 7.1 | 3.2 | Ma et al. 2015 |
| Ruminant adipose | Cattle |  | Radius | Qijia | Qijiaping (Gansu, China) | -17.2 | 7.7 | 3.2 | Ma et al. 2015 |
| Ruminant adipose | Cattle |  | Pelvis | Qijia | Qijiaping (Gansu, China) | -17.8 | 8.2 | 3.2 | Ma et al. 2015 |
| Ruminant adipose | Cattle |  | Tibia | Qijia | Qijiaping (Gansu, China) | -15.8 | 6.9 | 3.2 | Ma et al. 2015 |
| Ruminant adipose | Cattle |  | Phalanx III | Qijia | Qijiaping (Gansu, China) | -17.4 | 6.7 | 3.2 | Ma et al. 2015 |
| Ruminant adipose | Cattle |  | Mandible | Qijia | Qijiaping (Gansu, China) | -13.0 | 6.9 | 3.2 | Ma et al. 2015 |
| Ruminant adipose |  | Ovis aries/Capra aegagrus hircus | Bone | Machang | Mozuizi | -18.0 | 7.1 | 3.2 | Yang et al. 2019 |
| Ruminant adipose |  | Ovis aries/Capra aegagrus hircus | Bone | Machang | Mozuizi | -16.8 | 6.3 | 3.2 | Yang et al. 2019 |
| Ruminant adipose |  | Ovis aries/Capra aegagrus hircus | Bone | Machang | Mozuizi | -16.8 | 5.5 | 3.1 | Yang et al. 2019 |
| Ruminant adipose |  | Bos taurus | Bone | Machang | Mozuizi | -16.9 | 6.3 | 3.2 | Yang et al. 2019 |
| Ruminant adipose |  | Ovis aries/Capra aegagrus hircus | Bone | Machang | Guojiashan | -19.3 | 4.3 | 3.1 | Yang et al. 2019 |
| Ruminant adipose |  | Ovis aries/Capra aegagrus hircus | Bone | Machang | Guojiashan | -17.8 | 6.4 | 3.1 | Yang et al. 2019 |
| Ruminant adipose |  | Ovis aries/Capra aegagrus hircus | Bone | Machang | Guojiashan | -16.8 | 3.0 | 3.1 | Yang et al. 2019 |
| Ruminant adipose |  | Ovis aries/Capra aegagrus hircus | Bone | Machang | Xinzhai | -15.7 | 5.6 | 3.1 | Yang et al. 2019 |
| Ruminant adipose |  | Ovis aries/Capra aegagrus hircus | Bone | Machang | Xinzhai | -17.4 | 7.0 | 3.1 | Yang et al. 2019 |
| Ruminant adipose |  | Ovis aries/Capra aegagrus hircus | Bone | Machang | Shuikou | -17.3 | 7.2 | 3.2 | Yang et al. 2019 |
| Ruminant adipose |  | Ovis aries/Capra aegagrus hircus | Bone | Machang | Shuikou | -15.0 | 7.9 | 3.1 | Yang et al. 2019 |
| Ruminant adipose |  | Capreolus pygargus | Bone | Machang | Shuikou | -17.8 | 4.5 | 3.1 | Yang et al. 2019 |
| Ruminant adipose |  | Ovis aries/Capra aegagrus hircus | Bone | Machang | Shuikou | -18.1 | 8.3 | 3.1 | Yang et al. 2019 |
| Ruminant adipose |  | Ovis aries/Capra aegagrus hircus | Bone | Machang | Shuikou | -17.5 | 9.0 | 3.2 | Yang et al. 2019 |
| Ruminant adipose |  | Ovis aries/Capra aegagrus hircus | Bone | Machang | Qipanshan | -16.8 | 10.2 | 3.1 | Yang et al. 2019 |
| Ruminant adipose |  | Ovis aries/Capra aegagrus hircus | Bone | Qijia | Lijiageleng | -15.4 | 5.0 | 3.1 | Yang et al. 2019 |
| Ruminant adipose |  | Ovis aries/Capra aegagrus hircus | Bone | Siba | Dadunwan | -17.9 | 5.3 | 3.1 | Yang et al. 2019 |
| Ruminant adipose |  | Ovis aries/Capra aegagrus hircus | Bone | Siba | Dadunwan | -18.8 | 7.4 | 3.2 | Yang et al. 2019 |
| Ruminant adipose |  | Ovis aries/Capra aegagrus hircus | Bone | Siba | Dadunwan | -17.3 | 5.4 | 3.1 | Yang et al. 2019 |
| Ruminant adipose |  | Ovis aries/Capra aegagrus hircus | Bone | Siba | Xihuishan | -17.3 | 6.9 | 3.1 | Yang et al. 2019 |
| Ruminant adipose |  | Ovis aries/Capra aegagrus hircus | Bone | Siba | Xihuishan | -10.1 | 4.3 | 3.2 | Yang et al. 2019 |
| Ruminant adipose |  | Ovis aries/Capra aegagrus hircus | Bone | Siba | Xihuishan | -19.0 | 3.0 | 3.1 | Yang et al. 2019 |
| Ruminant adipose |  | Ovis aries/Capra aegagrus hircus | Bone | Siba | Xihuishan | -18.7 | 5.0 | 3.1 | Yang et al. 2019 |
| Ruminant adipose |  | Ovis aries/Capra aegagrus hircus | Bone | Siba | Ganguya | -17.8 | 6.2 | 3.1 | Yang et al. 2019 |
| Ruminant adipose |  | Ovis aries/Capra aegagrus hircus | Bone | Siba | Ganguya | -17.7 | 4.4 | 3.1 | Yang et al. 2019 |
| Ruminant adipose |  | Ovis aries/Capra aegagrus hircus | Bone | Siba | Ganguya | -16.8 | 6.2 | 3.2 | Yang et al. 2019 |
| Ruminant adipose |  | Ovis aries/Capra aegagrus hircus | Bone | Siba | Ganguya | -16.3 | 7.0 | 3.2 | Yang et al. 2019 |
| Ruminant adipose |  | Ovis aries/Capra aegagrus hircus | Bone | Dongjiatai | Tuba | -18.5 | 7.0 | 3.1 | Yang et al. 2019 |
| Ruminant adipose |  | Ovis aries/Capra aegagrus hircus | Bone | Dongjiatai | Tuba | -19.6 | 2.9 | 3.2 | Yang et al. 2019 |
| Ruminant adipose |  | Ovis aries/Capra aegagrus hircus | Bone | Shanman | Gudongtan | -18.4 | 9.8 | 3.1 | Yang et al. 2019 |
| Ruminant adipose |  | Ovis aries/Capra aegagrus hircus | Bone | Shanman | Gudongtan | -18.3 | 9.6 | 3.1 | Yang et al. 2019 |
| Ruminant adipose |  | Ovis aries/Capra aegagrus hircus | Bone | Shanman | Gudongtan | -18.5 | 8.0 | 3.1 | Yang et al. 2019 |
| Ruminant adipose |  | Ovis aries/Capra aegagrus hircus | Bone | Shanman | Zhaojiashuimo | -19.0 | 5.3 | 3.2 | Yang et al. 2019 |
| Ruminant adipose |  | Ovis aries/Capra aegagrus hircus | Bone | Shanman | Zhaojiashuimo | -16.9 | 6.1 | 3.1 | Yang et al. 2019 |
| Ruminant adipose |  | Ovis aries/Capra aegagrus hircus | Bone | Shajing | Minqinsanjiaozhou | -18.6 | 9.2 | 3.1 | Yang et al. 2019 |
| Ruminant adipose |  | Ovis aries/Capra aegagrus hircus | Bone | Shajing | Minqinsanjiaozhou | -17.4 | 6.3 | 3.2 | Yang et al. 2019 |
| Ruminant adipose |  | Ovis aries/Capra aegagrus hircus | Bone | Eastern Zhou | Shichengshan | -16.8 | 6.3 | 3.1 | Yang et al. 2019 |
| Ruminant adipose |  | Ovis aries/Capra aegagrus hircus | Bone | Eastern Zhou | Shichengshan | -16.7 | 5.1 | 3.2 | Yang et al. 2019 |
| Ruminant adipose |  | Capreolus pygargus | Bone | Eastern Zhou | Shichengshan | -17.0 | 5.9 | 3.2 | Yang et al. 2019 |
| Ruminant adipose |  | Capreolus pygargus | Bone | Eastern Zhou | Shichengshan | -18.2 | 5.6 | 3.1 | Yang et al. 2019 |
| Ruminant adipose |  | Caprinae | Bone | 2135-1692 BC | Huoshiliang (NW Gansu) | -18.1 | 6.3 | 3.3 | Atahan et al. 2011 |
| Ruminant adipose |  | Caprinae | Bone | 2135-1692 BC | Huoshiliang (NW Gansu) | -18.4 | 7.9 | 3.2 | Atahan et al. 2011 |
| Ruminant adipose |  | Caprinae | Bone | 2298-1758 BC | Gangangwa (NW Gansu) | -15.6 | 10.0 | 3.3 | Atahan et al. 2011 |
| Ruminant adipose |  | Ovicaprid | Metacarpal | 3600-3000 BP | Gongshijia (Gansu) | -15.3 | 5.5 | 3.1 | Ma et al. 2016 |
| Ruminant adipose |  | Bos taurus | Bone | Machang | Xinzhai | -16.4 | 5.3 | 3.1 | Yang et al. 2019 |
| Ruminant adipose |  | Bos taurus | Bone | Siba | Ganguya | -19.2 | 4.8 | 3.1 | Yang et al. 2019 |
| Ruminant adipose |  | Bos taurus | Bone | Shanman | Gudongtan | -20.0 | 4.1 | 3.1 | Yang et al. 2019 |
| Ruminant adipose |  | Bos taurus | Bone | Shanman | Gudongtan | -18.6 | 10.2 | 3.1 | Yang et al. 2019 |
| Ruminant adipose |  | Bos taurus | Bone | Shanman | Gudongtan | -17.9 | 6.1 | 3.1 | Yang et al. 2019 |
| Ruminant adipose |  | Bos taurus | Bone | Shanman | Gudongtan | -15.1 | 5.9 | 3.1 | Yang et al. 2019 |
| Ruminant adipose |  | Bos taurus | Bone | Shajing | Minqinsanjiaozhou | -18.6 | 7.7 | 3.1 | Yang et al. 2019 |
| Ruminant adipose |  | Bos taurus | Bone | Shajing | Minqinsanjiaozhou | -19.6 | 7.7 | 3.1 | Yang et al. 2019 |
| Ruminant adipose |  | Bos taurus | Bone | Shajing | Minqinsanjiaozhou | -18.6 | 8.2 | 3.1 | Yang et al. 2019 |
| Ruminant adipose |  | Bos taurus | Bone | Eastern Zhou | Shichengshan | -19.3 | 8.1 | 3.1 | Yang et al. 2019 |
| Ruminant adipose |  | Bos | Bone | 2135-1692 BC | Huoshiliang (NW Gansu) | -19.0 | 4.1 | 3.2 | Atahan et al. 2011 |
| Ruminant adipose |  | Bos | Bone | 2298-1758 BC | Gangangwa (NW Gansu) | -18.6 | 5.3 | 3.2 | Atahan et al. 2011 |
| Ruminant adipose |  | Bos | Bone | 2298-1758 BC | Gangangwa (NW Gansu) | -18.2 | 8.5 | 3.3 | Atahan et al. 2011 |
| Ruminant adipose |  | Bos | Bone | 2298-1758 BC | Gangangwa (NW Gansu) | -18.3 | 9.3 | 3.2 | Atahan et al. 2011 |
| Ruminant adipose |  | Bos | Bone | 2298-1758 BC | Gangangwa (NW Gansu) | -18.4 | 7.3 | 3.2 | Atahan et al. 2011 |
| Ruminant adipose |  | Bos | Phalanx I | 3600-3000 BP | Gongshijia (Gansu) | -9.8 | 7.0 | 3.3 | Ma et al. 2016 |
| Ruminant adipose |  | Bos | Pelvis | 3600-3000 BP | Wenjia (Gansu) | -14.8 | 4.4 | 3.2 | Ma et al. 2016 |
| Ruminant adipose |  | Bos | Skull | 3600-3000 BP | Wenjia (Gansu) | -13.5 | 5.9 | 3.2 | Ma et al. 2016 |
| Ruminant adipose |  | Cervus sp. | Bone | Machang | Shuikou | -18.3 | 7.2 | 3.1 | Yang et al. 2019 |
| Ruminant adipose |  | Cervus sp. | Bone | Siba | Dadunwan | -17.0 | 6.0 | 3.2 | Yang et al. 2019 |
| Ruminant adipose |  | Cervinae | Bone | 2135-1692 BC | Huoshiliang (NW Gansu) | -18.9 | 5.8 | 3.2 | Atahan et al. 2011 |
| Ruminant adipose |  | Cervus | Bone | 2135-1692 BC | Huoshiliang (NW Gansu) | -18.9 | 5.0 | 3.2 | Atahan et al. 2011 |
| Ruminant adipose |  | Cervus | Bone | 2135-1692 BC | Huoshiliang (NW Gansu) | -18.5 | 5.9 | 3.3 | Atahan et al. 2011 |
| Ruminant adipose |  | Cervus | Bone | 2135-1692 BC | Huoshiliang (NW Gansu) | -19.4 | 4.9 | 3.2 | Atahan et al. 2011 |
| Ruminant adipose |  | Cervinae | Bone | 2135-1692 BC | Huoshiliang (NW Gansu) | -19.0 | 5.4 | 3.2 | Atahan et al. 2011 |
| Ruminant adipose |  | Cervinae | Bone | 2135-1692 BC | Huoshiliang (NW Gansu) | -15.2 | 6.5 | 3.2 | Atahan et al. 2011 |
| Ruminant adipose |  | Cervinae | Bone | 2298-1758 BC | Gangangwa (NW Gansu) | -17.9 | 9.4 | 3.2 | Atahan et al. 2011 |
| Ruminant adipose |  | Cervinae | Bone | 2298-1758 BC | Gangangwa (NW Gansu) | -14.8 | 7.2 | 3.2 | Atahan et al. 2011 |
| Ruminant adipose |  | Cervus | Phalanx I | 3600-3000 BP | Gongshijia (Gansu) | -19.5 | 4.2 | 3.5 | Ma et al. 2016 |
| Ruminant adipose |  | Cervus | Phalanx I | 3600-3000 BP | Gongshijia (Gansu) | -19.6 | 4.1 | 3.3 | Ma et al. 2016 |
| Ruminant adipose |  | Cervus | Horn | 3600-3000 BP | Mogou (Gansu) | -20.0 | 3.5 | 2.3 | Ma et al. 2016 |
| Ruminant adipose |  | Cervus | Bone | 3600-3000 BP | Mogou (Gansu) | -20.0 | 5.1 | 2.7 | Ma et al. 2016 |
| Non-ruminant adipose | Horse |  | Radius | Qijia | Qijiaping (Gansu) | -16.3 | 6.7 | 3.2 | Ma et al. 2015 |
| Non-ruminant adipose | Horse |  | Pelvis | Qijia | Qijiaping (Gansu) | -17.4 | 8.5 | 3.2 | Ma et al. 2015 |
| Non-ruminant adipose |  | Equus ferus caballus | Bone | Dongjiatai | Tuba | -19.7 | 4.3 | 3.1 | Yang et al. 2019 |
| Non-ruminant adipose |  | Equus ferus caballus | Bone | Dongjiatai | Tuba | -19.7 | 4.1 | 3.1 | Yang et al. 2019 |
| Non-ruminant adipose |  | Equus ferus caballus | Bone | Shanman | Gudongtan | -17.9 | 5.9 | 3.1 | Yang et al. 2019 |
| Non-ruminant adipose |  | Equus ferus caballus | Bone | Shanman | Gudongtan | -19.8 | 5.8 | 3.2 | Yang et al. 2019 |
| Non-ruminant adipose |  | Equus ferus caballus | Bone | Shanman | Gudongtan | -17.8 | 7.0 | 3.2 | Yang et al. 2019 |
| Non-ruminant adipose |  | Equus ferus caballus | Bone | Shanman | Zhaojiashuimo | -18.1 | 5.5 | 3.1 | Yang et al. 2019 |
| Non-ruminant adipose |  | Equus ferus caballus | Bone | Eastern Zhou | Shichengshan | -19.6 | 5.9 | 3.1 | Yang et al. 2019 |
| Non-ruminant adipose |  | Equus ferus caballus | Bone | Eastern Zhou | Shichengshan | -20.5 | 5.9 | 3.1 | Yang et al. 2019 |
| Non-ruminant adipose | Medium Rodent |  | Bone | Bronze Age | Dayatou (Gansu) | -7.1 | 6.7 | 2.8 | this study |
| Non-ruminant adipose | Medium Rodent |  | Bone | Bronze Age | Dayatou (Gansu) | -7.0 | 7.4 | 3.0 | this study |
| Non-ruminant adipose | Medium Rodent |  | Bone | Bronze Age | Dayatou (Gansu) | -7.0 | 7.5 | 3.0 | this study |
| Non-ruminant adipose | Rodent |  | Bone | Bronze Age | Dayatou (Gansu) | -15.9 | 7.9 | 2.9 | this study |
| Non-ruminant adipose | Medium Rodent |  | Bone | Bronze Age | Dayatou (Gansu) | -21.2 | 6.7 | 2.9 | this study |
| Non-ruminant adipose | Rodent |  | Bone | Bronze Age | Huizuiwa (Gansu) | -8.2 | 7.9 | 2.9 | this study |
| Non-ruminant adipose | Rodent |  | Bone | Bronze Age | Huizuiwa (Gansu) | -7.6 | 9.7 | 2.9 | this study |
| Non-ruminant adipose | Medium Rodent |  | Bone | Bronze Age | Huizuiwa (Gansu) | -17.2 | 7.4 | 2.9 | this study |
| Non-ruminant adipose | Medium Rodent |  | Bone | Bronze Age | Huizuiwa (Gansu) | -24.3 | 7.6 | 3.0 | this study |
| Non-ruminant adipose | Medium Rodent |  | Bone | Bronze Age | Huizuiwa (Gansu) | -13.4 | 7.7 | 3.2 | this study |
| Non-ruminant adipose | Medium Rodent |  | Bone | Bronze Age | Qijiaping (Gansu) | -21.8 | 12.4 | 3.2 | this study |
| Non-ruminant adipose | Rodent |  | Bone | Bronze Age | Qijiaping (Gansu) | -17.4 | 4.3 | 3.3 | this study |
| Non-ruminant adipose | Rodent |  | Bone | Bronze Age | Qijiaping (Gansu) | -21.3 | 12.7 | 3.4 | this study |
| Non-ruminant adipose |  | Hystrix | Bone | Bronze Age | Qijiaping (Gansu) | -23.1 | 3.4 |  | this study |
| Non-ruminant adipose |  | Rattus | Bone | 2135-1692 BC | Huoshiliang (NW Gansu) | -8.1 | 8.7 | 3.3 | Atahan et al. 2011 |
| Non-ruminant adipose |  | Rattus | Bone | 2135-1692 BC | Huoshiliang (NW Gansu) | -8.8 | 8.2 | 3.2 | Atahan et al. 2011 |
| Non-ruminant adipose |  | Rattus | Bone | 2298-1758 BC | Gangangwa (NW Gansu) | -10.4 | 11.3 | 3.3 | Atahan et al. 2011 |
| Non-ruminant adipose |  | Lepus? | Bone | Bronze Age | Huizuiwa (Gansu, China) | -18.8 | 3.5 | 3.0 | this study |
| Non-ruminant adipose |  | Lepus sp. | Bone | Bronze Age | Dayatou (Gansu, China) | -16.8 | 4.7 | 2.9 | this study |
| Non-ruminant adipose |  | Lepus sp. | Bone | Bronze Age | Qijiaping (Gansu, China) | -15.2 | 5.5 | 3.1 | this study |
| Non-ruminant adipose | Bird | Possibly Gallus | Bone | DDW | Dadiwan (NW China) | -15.6 | 7.4 | 2.7 | Barton et al. 2009 |
| Non-ruminant adipose | Bird | Possibly Gallus | Bone | DDW | Dadiwan (NW China) | -16.8 | 7.4 | 2.7 | Barton et al. 2009 |
| Non-ruminant adipose | Bird | Possibly Gallus | Bone | LBP | Dadiwan (NW China) | -16.2 | 7.1 | 2.8 | Barton et al. 2009 |
| Non-ruminant adipose | Bird | Possibly Gallus | Bone | LBP | Dadiwan (NW China) | -17.6 | 6.3 | 2.7 | Barton et al. 2009 |
| Non-ruminant adipose | Bird | Possibly Gallus | Bone | LBP | Dadiwan (NW China) | -14.2 | 5.9 | 2.8 | Barton et al. 2009 |
| Non-ruminant adipose | Bird | Possibly Gallus | Bone | LBP | Dadiwan (NW China) | -17.2 | 5.7 | 2.8 | Barton et al. 2009 |
| Non-ruminant adipose | Bird | Possibly Gallus | Bone | LBP | Dadiwan (NW China) | -16.2 | 6.5 | 2.8 | Barton et al. 2009 |
| Omnivorous adipose |  | Sus sp. | Bone | Bronze Age | Dayatou (Gansu) | -9.4 | 7.1 | 2.9 | this study |
| Omnivorous adipose |  | Sus sp. | Bone | Bronze Age | Dayatou (Gansu) | -7.6 | 7.3 | 3.0 | this study |
| Omnivorous adipose |  | Sus sp. | Bone | Bronze Age | Dayatou (Gansu) | -8.5 | 7.5 | 2.9 | this study |
| Omnivorous adipose |  | Sus sp. | Bone | Bronze Age | Dayatou (Gansu) | -9.0 | 7.8 | 3.0 | this study |
| Omnivorous adipose |  | Sus sp. | Bone | Bronze Age | Dayatou (Gansu) | -8.2 | 7.9 | 3.0 | this study |
| Omnivorous adipose |  | Sus sp. | Bone | Bronze Age | Dayatou (Gansu) | -6.9 | 7.9 | 2.8 | this study |
| Omnivorous adipose |  | Sus sp. | Bone | Bronze Age | Dayatou (Gansu) | -7.6 | 7.9 | 2.9 | this study |
| Omnivorous adipose |  | Sus sp. | Bone | Bronze Age | Dayatou (Gansu) | -7.7 | 8.3 | 2.9 | this study |
| Omnivorous adipose |  | Sus sp.? | Bone | Bronze Age | Huizuiwa (Gansu) | -15.9 | 6.2 | 2.9 | this study |
| Omnivorous adipose |  | Sus sp. | Bone | Bronze Age | Huizuiwa (Gansu) | -6.9 | 7.5 | 3.0 | this study |
| Omnivorous adipose |  | Sus sp. | Bone | Bronze Age | Qijiaping (Gansu) | -9.4 | 7.1 | 3.2 | this study |
| Omnivorous adipose |  | Sus sp. | Bone | Bronze Age | Qijiaping (Gansu) | -9.7 | 7.4 | 3.2 | this study |
| Omnivorous adipose |  | Sus sp. | Bone | Bronze Age | Qijiaping (Gansu) | -10.1 | 7.7 | 3.3 | this study |
| Omnivorous adipose |  | Sus sp. | Bone | Bronze Age | Qijiaping (Gansu) | -9.0 | 7.2 | 3.2 | this study |
| Omnivorous adipose |  | Sus sp. | Bone | Bronze Age | Qijiaping (Gansu) | -10.8 | 7.9 | 3.3 | this study |
| Omnivorous adipose |  | Sus sp. | Bone | Bronze Age | Qijiaping (Gansu) | -7.8 | 7.9 | 3.2 | this study |
| Omnivorous adipose |  | Sus sp. | Bone | Bronze Age | Qijiaping (Gansu) | -10.1 | 6.7 | 3.3 | this study |
| Omnivorous adipose |  | Sus sp. | Bone | LBP | Dadiwan (NW China) | -8.5 | 8.0 | 2.8 | Barton et al. 2009 |
| Omnivorous adipose |  | Sus sp. | Bone | LBP | Dadiwan (NW China) | -15.7 | 7.2 | 2.8 | Barton et al. 2009 |
| Omnivorous adipose |  | Sus sp. | Bone | LBP | Dadiwan (NW China) | -8.3 | 8.4 | 2.7 | Barton et al. 2009 |
| Omnivorous adipose |  | Sus sp. | Bone | LBP | Dadiwan (NW China) | -14.7 | 8.5 | 2.7 | Barton et al. 2009 |
| Omnivorous adipose |  | Sus sp. | Bone | LBP | Dadiwan (NW China) | -17.5 | 6.4 | 2.7 | Barton et al. 2009 |
| Omnivorous adipose |  | Sus sp. | Bone | LBP | Dadiwan (NW China) | -7.0 | 7.7 | 2.8 | Barton et al. 2009 |
| Omnivorous adipose |  | Sus sp. | Bone | LBP | Dadiwan (NW China) | -9.7 | 9.2 | 2.7 | Barton et al. 2009 |
| Omnivorous adipose |  | Sus sp. | Bone | LBP | Dadiwan (NW China) | -9.0 | 9.2 | 2.7 | Barton et al. 2009 |
| Omnivorous adipose |  | Sus sp. | Bone | LBP | Dadiwan (NW China) | -10.0 | 8.8 | 2.7 | Barton et al. 2009 |
| Omnivorous adipose |  | Sus sp. | Bone | LBP | Dadiwan (NW China) | -6.3 | 8.1 | 2.8 | Barton et al. 2009 |
| Omnivorous adipose |  | Sus sp. | Bone | LBP | Dadiwan (NW China) | -6.5 | 8.6 | 2.7 | Barton et al. 2009 |
| Omnivorous adipose |  | Sus sp. | Bone | LBP | Dadiwan (NW China) | -8.8 | 8.4 | 2.7 | Barton et al. 2009 |
| Omnivorous adipose |  | Sus sp. | Bone | LBP | Dadiwan (NW China) | -11.5 | 9.9 | 2.8 | Barton et al. 2009 |
| Omnivorous adipose |  | Sus sp. | Bone | LBP | Dadiwan (NW China) | -12.2 | 8.9 | 2.8 | Barton et al. 2009 |
| Omnivorous adipose |  | Sus sp. | Bone | LBP | Dadiwan (NW China) | -9.1 | 9.1 | 3.0 | Barton et al. 2009 |
| Omnivorous adipose |  | Sus sp. | Bone | LBP | Dadiwan (NW China) | -11.4 | 9.6 | 2.7 | Barton et al. 2009 |
| Omnivorous adipose |  | Sus sp. | Bone | LBP | Dadiwan (NW China) | -9.2 | 8.8 | 2.8 | Barton et al. 2009 |
| Omnivorous adipose |  | Sus sp. | Bone | LBP | Dadiwan (NW China) | -8.2 | 9.1 | 2.8 | Barton et al. 2009 |
| Omnivorous adipose |  | Sus sp. | Bone | LBP | Dadiwan (NW China) | -8.3 | 8.7 | 2.9 | Barton et al. 2009 |
| Omnivorous adipose |  | Sus sp. | Bone | LBP | Dadiwan (NW China) | -14.9 | 6.8 | 2.8 | Barton et al. 2009 |
| Omnivorous adipose |  | Sus sp. | Bone | LBP | Dadiwan (NW China) | -9.0 | 8.4 | 2.7 | Barton et al. 2009 |
| Omnivorous adipose |  | Sus sp. | Bone | LBP | Dadiwan (NW China) | -9.0 | 8.6 | 2.8 | Barton et al. 2009 |
| Omnivorous adipose |  | Sus sp. | Bone | LBP | Dadiwan (NW China) | -7.7 | 7.9 | 2.8 | Barton et al. 2009 |
| Omnivorous adipose |  | Sus sp. | Bone | LBP | Dadiwan (NW China) | -8.3 | 8.9 | 2.8 | Barton et al. 2009 |
| Omnivorous adipose |  | Sus sp. | Bone | LBP | Dadiwan (NW China) | -9.0 | 8.2 | 2.8 | Barton et al. 2009 |
| Omnivorous adipose |  | Sus sp. | Bone | LBP | Dadiwan (NW China) | -11.0 | 7.8 | 2.7 | Barton et al. 2009 |
| Omnivorous adipose |  | Sus sp. | Bone | LBP | Dadiwan (NW China) | -12.3 | 9.3 | 2.8 | Barton et al. 2009 |
| Omnivorous adipose |  | Sus sp. | Bone | DDW | Dadiwan (NW China) | -12.0 | 8.3 | 2.7 | Barton et al. 2009 |
| Omnivorous adipose |  | Sus sp. | Bone | DDW | Dadiwan (NW China) | -16.3 | 6.2 | 2.8 | Barton et al. 2009 |
| Omnivorous adipose |  | Sus sp. | Bone | DDW | Dadiwan (NW China) | -20.4 | 5.6 | 2.9 | Barton et al. 2009 |
| Omnivorous adipose |  | Sus sp. | Bone | DDW | Dadiwan (NW China) | -20.9 | 7.2 | 2.8 | Barton et al. 2009 |
| Omnivorous adipose |  | Sus sp. | Bone | NA | Dadiwan (NW China) | -19.6 | 5.2 | 2.9 | Barton et al. 2009 |
| Omnivorous adipose |  | Sus sp. | Bone | DDW | Dadiwan (NW China) | -19.3 | 5.3 | 2.8 | Barton et al. 2009 |
| Omnivorous adipose |  | Sus sp. | Bone | DDW | Dadiwan (NW China) | -19.1 | 7.0 | 2.8 | Barton et al. 2009 |
| Omnivorous adipose |  | Sus sp. | Bone | DDW | Dadiwan (NW China) | -19.0 | 5.6 | 2.8 | Barton et al. 2009 |
| Omnivorous adipose |  | Sus sp. | Bone | LBP | Dadiwan (NW China) | -8.3 | 7.6 | 2.8 | Barton et al. 2009 |
| Omnivorous adipose |  | Sus sp. | Rib | Machang | Xiahaishi (Loess Plateau) | -7.3 | 6.1 | 3.2 | Ma et al. 2014 |
| Omnivorous adipose |  | Sus sp. | Ulna | Machang | Xiahaishi (Loess Plateau) | -6.8 | 5.8 | 3.2 | Ma et al. 2014 |
| Omnivorous adipose |  | Sus sp. | Long bone | Machang | Xiahaishi (Loess Plateau) | -8.3 | 7.2 | 3.2 | Ma et al. 2014 |
| Omnivorous adipose |  | Sus sp. | Tibia | Machang | Xiahaishi (Loess Plateau) | -7.5 | 7.3 | 3.2 | Ma et al. 2014 |
| Omnivorous adipose |  | Sus sp. | Long bone | Machang | Xiahaishi (Loess Plateau) | -6.8 | 4.7 | 3.2 | Ma et al. 2014 |
| Omnivorous adipose |  | Sus sp. | Rib | Machang | Xiahaishi (Loess Plateau) | -15.0 | 4.9 | 3.2 | Ma et al. 2014 |
| Omnivorous adipose |  | Sus sp. | Mandible | Machang | Xiahaishi (Loess Plateau) | -7.7 | 7.4 | 3.2 | Ma et al. 2014 |
| Omnivorous adipose |  | Sus sp. | Ulna | Machang | Xiahaishi (Loess Plateau) | -7.1 | 7.9 | 3.2 | Ma et al. 2014 |
| Omnivorous adipose |  | Sus sp. | Maxilla | Machang | Xiahaishi (Loess Plateau) | -9.7 | 8.9 | 3.2 | Ma et al. 2014 |
| Omnivorous adipose |  | Sus domesticus | Mandible | Machang | Xiahaishi (Loess Plateau) | -7.8 | 6.7 | 3.2 | Ma et al. 2014 |
| Omnivorous adipose |  | Suidae | Mandible | Machang | Xiahaishi (Loess Plateau) | -22.7 | 2.4 | 3.2 | Ma et al. 2014 |
| Omnivorous adipose | Pig, subadult | Sus sp. | Scapula | Qijia | Qijiaping (Gansu) | -8.0 | 8.1 | 3.2 | Ma et al. 2015 |
| Omnivorous adipose | Pig, subadult | Sus sp. | Skull | Qijia | Qijiaping (Gansu) | -8.3 | 7.6 | 3.2 | Ma et al. 2015 |
| Omnivorous adipose | Pig, subadult | Sus sp. | Mandible | Qijia | Qijiaping (Gansu) | -9.2 | 7.1 | 3.2 | Ma et al. 2015 |
| Omnivorous adipose | Pig, subadult | Sus sp. | Mandible | Qijia | Qijiaping (Gansu) | -7.1 | 8.7 | 3.2 | Ma et al. 2015 |
| Omnivorous adipose | Pig, adult | Sus sp. | Tibia | Qijia | Qijiaping (Gansu) | -17.5 | 6.0 | 3.2 | Ma et al. 2015 |
| Omnivorous adipose | Pig, adult | Sus sp. | Pelvis | Qijia | Qijiaping (Gansu) | -7.9 | 7.3 | 3.2 | Ma et al. 2015 |
| Omnivorous adipose | Pig, adult | Sus sp. | Mandible | Qijia | Qijiaping (Gansu) | -10.0 | 7.4 | 3.2 | Ma et al. 2015 |
| Omnivorous adipose | Pig, adult | Sus sp. | Skull | Qijia | Qijiaping (Gansu) | -9.8 | 7.2 | 3.2 | Ma et al. 2015 |
| Omnivorous adipose | Pig, adult | Sus sp. | Pelvis | Qijia | Qijiaping (Gansu) | -13.0 | 7.2 | 3.2 | Ma et al. 2015 |
| Omnivorous adipose |  | Sus scrofa domesticus | Bone | Machang | Shuikou | -7.5 | 6.4 | 3.1 | Yang et al. 2019 |
| Omnivorous adipose |  | Sus scrofa domesticus | Bone | Machang | Qipanshan | -8.6 | 6.9 | 3.2 | Yang et al. 2019 |
| Omnivorous adipose |  | Sus scrofa domesticus | Bone | Qijia | Lijiageleng | -7.2 | 8.4 | 3.1 | Yang et al. 2019 |
| Omnivorous adipose |  | Sus scrofa domesticus | Bone | Qijia | Lijiageleng | -9.0 | 6.6 | 3.1 | Yang et al. 2019 |
| Omnivorous adipose |  | Sus scrofa domesticus | Bone | Siba | Xihuishan | -7.8 | 8.1 | 3.1 | Yang et al. 2019 |
| Omnivorous adipose |  | Sus scrofa domesticus | Bone | Siba | Ganguya | -12.2 | 9.0 | 3.1 | Yang et al. 2019 |
| Omnivorous adipose |  | Sus | Bone | 2135-1692 BC | Huoshiliang (NW Gansu) | -19.3 | 7.1 | 3.2 | Atahan et al. 2011 |
| Omnivorous adipose |  | Sus | Bone | 2135-1692 BC | Huoshiliang (NW Gansu) | -7.7 | 8.0 | 3.2 | Atahan et al. 2011 |
| Omnivorous adipose |  | Sus | Bone | 2298-1758 BC | Gangangwa (NW Gansu) | -16.0 | 12.2 | 3.2 | Atahan et al. 2011 |
| Omnivorous adipose |  | Sus | Bone | 2298-1758 BC | Gangangwa (NW Gansu) | -7.9 | 9.2 | 3.2 | Atahan et al. 2011 |
| Omnivorous adipose |  | Canidae | Bone | Bronze Age | Huizuiwa (Gansu) | -7.7 | 7.7 | 2.9 | this study |
| Omnivorous adipose |  | Canidae | Bone | Bronze Age | Huizuiwa (Gansu) | -7.7 | 7.7 | 2.9 | this study |
| Omnivorous adipose |  | Canis familiaris | Bone | Bronze Age | Huizuiwa (Gansu) | -18.8 | 7.7 | 2.9 | this study |
| Omnivorous adipose |  | Canidae | Bone | Bronze Age | Huizuiwa (Gansu) | -12.1 | 8.2 | 3.0 | this study |
| Omnivorous adipose |  | Canis familiaris | Bone | Bronze Age | Qijiaping (China) | -8.8 | 7.9 | 3.2 | this study |
| Omnivorous adipose |  | Canis familiaris | Bone | Bronze Age | Qijiaping (China) | -8.8 | 7.6 | 3.3 | this study |
| Omnivorous adipose |  | Canis | Bone | LBP | Dadiwan (NW China) | -7.9 | 7.9 | 2.8 | Barton et al. 2009 |
| Omnivorous adipose |  | Canis | Bone | LBP | Dadiwan (NW China) | -8.2 | 8.6 | 2.8 | Barton et al. 2009 |
| Omnivorous adipose |  | Canis | Bone | LBP | Dadiwan (NW China) | -10.7 | 8.7 | 2.7 | Barton et al. 2009 |
| Omnivorous adipose |  | Canis | Bone | DDW | Dadiwan (NW China) | -19.9 | 6.2 | 2.8 | Barton et al. 2009 |
| Omnivorous adipose |  | Canis | Bone | DDW | Dadiwan (NW China) | -19.8 | 5.9 | 2.7 | Barton et al. 2009 |
| Omnivorous adipose |  | Canis | Bone | LBP | Dadiwan (NW China) | -9.3 | 9.0 | 2.8 | Barton et al. 2009 |
| Omnivorous adipose |  | Canis | Bone | LBP | Dadiwan (NW China) | -10.2 | 7.3 | 2.7 | Barton et al. 2009 |
| Omnivorous adipose |  | Canis | Bone | LBP | Dadiwan (NW China) | -13.0 | 8.6 | 2.8 | Barton et al. 2009 |
| Omnivorous adipose |  | Canis | Bone | DDW | Dadiwan (NW China) | -13.1 | 8.7 | 2.8 | Barton et al. 2009 |
| Omnivorous adipose |  | Canis | Bone | DDW | Dadiwan (NW China) | -10.2 | 7.5 | 2.8 | Barton et al. 2009 |
| Omnivorous adipose |  | Canis | Bone | DDW | Dadiwan (NW China) | -11.1 | 7.7 | 2.7 | Barton et al. 2009 |
| Omnivorous adipose |  | Sus sp. | Humerus | 3600-3000 BP | Gongshijia (Gansu,) | -20.4 | 3.7 | 3.3 | Ma et al. 2016 |
| Omnivorous adipose |  | Canis familiaris | Tibia | Machang | Xiahaishi (Loess Plateau) | -7.8 | 4.3 | 3.2 | Ma et al. 2014 |
| Omnivorous adipose | Dog |  | Mandible | Qijia | Qijiaping (Gansu) | -10.7 | 7.6 | 3.2 | Ma et al. 2015 |
| Omnivorous adipose | Dog |  | Mandible | Qijia | Qijiaping (Gansu) | -10.2 | 7.6 | 3.2 | Ma et al. 2015 |
| Omnivorous adipose |  | Canis lupus familiaris | Bone | Machang | Shuikou | -8.3 | 6.6 | 3.1 | Yang et al. 2019 |
| Omnivorous adipose |  | Canis lupus familiaris | Bone | Machang | Shuikou | -6.8 | 6.8 | 3.2 | Yang et al. 2019 |
| Omnivorous adipose |  | Canis lupus familiaris | Bone | Shajing | Minqinsanjiaozhou | -13.7 | 12.5 | 3.1 | Yang et al. 2019 |
| Omnivorous adipose |  | Canis lupus familiaris | Bone | Eastern Zhou | Shichengshan | -17.3 | 8.9 | 3.2 | Yang et al. 2019 |
| Omnivorous adipose |  | Canis | Bone | 2135-1692 BC | Huoshiliang (NW Gansu) | -11.7 | 8.4 | 3.2 | Atahan et al. 2011 |
| Omnivorous adipose |  | Canis | Bone | 2135-1692 BC | Huoshiliang (NW Gansu) | -11.6 | 8.3 | 3.2 | Atahan et al. 2011 |
| Omnivorous adipose |  | Canis | Bone | 2135-1692 BC | Huoshiliang (NW Gansu) | -15.8 | 6.8 | 3.2 | Atahan et al. 2011 |
| Omnivorous adipose |  | Canis | Tibia | 3600-3000 BP | Mogou (Gansu, China) | -16.8 | 7.9 | 2.4 | Ma et al. 2016 |
| Omnivorous adipose |  | Canis | Long bone | 3600-3000 BP | Mogou (Gansu) | -15.7 | 8.8 | 1.5 | Ma et al. 2016 |
| Omnivorous adipose |  | Ursus | Bone | DDW | Dadiwan (NW China) | -17.3 | 7.0 | 2.8 | Barton et al. 2009 |

**References**

An, Cheng-Bang, Weimiao Dong, Yufeng Chen, Hu Li, Chao Shi, Wei Wang, Pingyu Zhang, and Xueye Zhao

2015 Stable isotopic investigations of modern and charred foxtail millet and the implications for environmental archaeological reconstruction in the western Chinese Loess Plateau. *Quaternary Research* 84(1):144–149. DOI:[10.1016/j.yqres.2015.04.004](https://doi.org/10.1016/j.yqres.2015.04.004).

Atahan, Pia, John Dodson, Xiaoqiang Li, Xinying Zhou, Songmei Hu, Fiona Bertuch, and Nan Sun

2011 Subsistence and the isotopic signature of herding in the Bronze Age Hexi Corridor, NW Gansu, China. *Journal of Archaeological Science* 38(7):1747–1753. DOI:[10.1016/j.jas.2011.03.006](https://doi.org/10.1016/j.jas.2011.03.006).

Barton, L., S. D. Newsome, F.-H. Chen, H. Wang, T. P. Guilderson, and R. L. Bettinger

2009 Agricultural origins and the isotopic identity of domestication in northern China. *Proceedings of the National Academy of Sciences* 106(14):5523–5528. DOI:[10.1073/pnas.0809960106](https://doi.org/10.1073/pnas.0809960106).

Correa-Ascencio, Marisol, and Richard P. Evershed

2014 High throughput screening of organic residues in archaeological potsherds using direct acidified methanol extraction. *Analytical Methods* 6(5):1330. DOI:[10.1039/c3ay41678j](https://doi.org/10.1039/c3ay41678j).

Craig, O. E., M. Forster, S. H. Andersen, E. Koch, P. Crombé, N. J. Milner, B. Stern, G. N. Bailey, and C. P. Heron

2007 Molecular and isotopic demonstration of the processing of aquatic products in northern european prehistoric pottery. *Archaeometry* 49(1):135–152. DOI:[10.1111/j.1475-4754.2007.00292.x](https://doi.org/10.1111/j.1475-4754.2007.00292.x).

Ma, M. M., G. H. Dong, E. Lightfoot, H. Wang, X. Y. Liu, X. Jia, K. R. Zhang, and F. H. Chen

2014 Stable Isotope Analysis of Human and Faunal Remains in the Western Loess Plateau, Approximately 2000 cal bc. *Archaeometry* 56:237–255. DOI:[10.1111/arcm.12071](https://doi.org/10.1111/arcm.12071).

Ma, M., G. Dong, X. Liu, E. Lightfoot, F. Chen, H. Wang, H. Li, and M. K. Jones

2015 Stable Isotope Analysis of Human and Animal Remains at the Qijiaping Site in Middle Gansu, China: Stable Isotope Analysis of remains at Qijiaping Site, China. *International Journal of Osteoarchaeology* 25(6):923–934. DOI:[10.1002/oa.2379](https://doi.org/10.1002/oa.2379).

Ma, Minmin, Guanghui Dong, Xin Jia, Hui Wang, Yifu Cui, and Fahu Chen

2016 Dietary shift after 3600 cal yr BP and its influencing factors in northwestern China: Evidence from stable isotopes. *Quaternary Science Reviews* 145:57–70. DOI:[10.1016/j.quascirev.2016.05.041](https://doi.org/10.1016/j.quascirev.2016.05.041).

Morton, June D., and Henry P. Schwarcz

2004 Palaeodietary implications from stable isotopic analysis of residues on prehistoric Ontario ceramics. *Journal of Archaeological Science* 31(5):503–517. DOI:[10.1016/j.jas.2003.10.001](https://doi.org/10.1016/j.jas.2003.10.001).

Yang, Y., L. Ren, G. Dong, Y. Cui, R. Liu, G. Chen, H. Wang, S. Wilkin, and F. Chen

2019 Economic Change in the Prehistoric Hexi Corridor (4800-2200 bp ), North-West China: Economic change in the prehistoric Hexi Corridor. *Archaeometry* 61(4):957–976. DOI:[10.1111/arcm.12464](https://doi.org/10.1111/arcm.12464).

*Supplementary Table 2: Compound specific carbon values of reference materials used in this study*

| **Category** | **Diet** | **Common name** | **Taxa** | **Sample type** | **Period** | **Provenience** | **C_16:0_ δ^13^C** | **C_18:0_ δ^13^C** | **Reference** |
| --- | --- | --- | --- | --- | --- | --- | --- | --- | --- |
| C4 plants | NA | Common millet | *Panicum miliaceum* | Raw seeds | Modern | Poland | -19.8 | -19.7 | This study |
| C4 plants | NA | Common millet | *Panicum miliaceum* | Raw seeds | Modern | Poland | -19.8 | -19.4 | This study |
| C4 plants | NA | Common millet | *Panicum miliaceum* | Charred seeds | Modern | Poland | -19.4 | -19.5 | This study |
| C4 plants | NA | Common millet | *Panicum miliaceum* | Charred seeds | Modern | Poland | -19.5 | -20.0 | This study |
| C4 plants | NA | Common millet | *Panicum miliaceum* | Raw seed | Modern | Poland | -17.8 | -17.4 | This study |
| C4 plants | NA | Common millet | *Panicum miliaceum* | Raw seed | Modern | Poland | -19.3 | -20.0 | This study |
| C4 plants | NA | Common millet | *Panicum miliaceum* | Raw seed | Modern | Poland | -17.8 | -18.5 | This study |
| C4 plants | NA | Common millet | *Panicum miliaceum* | Raw seed | Modern | Poland | -17.3 | -18.2 | This study |
| C4 plants | NA | Common millet | *Panicum miliaceum* | Raw seed | Modern | Taiwan | -19.4 | -18.4 | March 2013 |
| C4 plants | NA | Common millet | *Panicum miliaceum* | Raw seed | Modern | Taiwan | -19.8 | -18.8 | March 2013 |
| Non-ruminant | C3 | Horse | *Equus caballus* | Adipose | Modern | UK | -29,7 | -29,4 | Dudd 1999 |
| Non-ruminant | C3 | Horse | *Equus caballus* | Adipose | Modern | UK | -30,6 | -30,1 | Dudd 1999 |
| Non-ruminant | C3 | Horse | *Equus caballus* | Adipose | Modern | UK | -30,5 | -29,6 | Dudd 1999 |
| Non-ruminant | C3 | Horse | *Equus caballus* | Adipose | Modern | UK | -30 | -29,2 | Dudd 1999 |
| Non-ruminant | C3 | Horse | *Equus caballus* | Adipose | Modern | UK | -30,3 | -29,9 | Dudd 1999 |
| Non-ruminant | C3 | Horse | *Equus caballus* | Adipose | Modern | UK | -29,5 | -29,6 | Dudd 1999 |
| Non-ruminant | C3 | Horse | *Equus caballus* | Adipose | Modern | UK | -29,6 | -27,5 | Dudd 1999 |
| Non-ruminant | C3 | Horse | *Equus caballus* | Adipose | Modern | UK | -29,9 | -29,7 | Dudd 1999 |
| Non-ruminant | C3 | Beaver | *Castor fiber* | Tissue | Modern | Estonia | -31,4 | -32,1 | Courel et al. 2020 |
| Non-ruminant | C3 | Beaver | *Castor fiber* | Tissue | Modern | Estonia | -31,3 | -32,1 | Courel et al. 2020 |
| Non-ruminant | C3 | Beaver | *Castor fiber* | Tissue | Modern | Estonia | -31,2 | -31,7 | Courel et al. 2020 |
| Non-ruminant | C3 | Beaver | *Castor fiber* | Tissue | Modern | Estonia | -31 | -31,6 | Courel et al. 2020 |
| Non-ruminant | C3 | Beaver | *Castor fiber* | Tissue | Modern | Estonia | -31 | -30,8 | Courel et al. 2020 |
| Non-ruminant | C3 | Beaver | *Castor fiber* | Tissue | Modern | Estonia | -30 | -30,2 | Courel et al. 2020 |
| Non-ruminant | C3 | Eurasian beaver | *Castor fiber* |  | Modern | Mehikoorma | -29,9 | -30 | Courel et al. 2020 |
| Non-ruminant | C3 | Beaver | *Castor fiber* | Tissue | Modern | Estonia | -29,3 | -29,7 | Courel et al. 2020 |
| Non-ruminant | C3 | Hare | *Lepus americanus* | Tissue | Modern | Canada | -31,2 | -31,6 | Taché and Craig 2015 |
| Non-ruminant | C3 | Racoon | *Procyon lotor* | Tissue | Modern | Canada | -28,2 | -27,7 | Taché and Craig 2015 |
| Non-ruminant | C3 | Muskrat | *Ondatra zibethicus* | Tissue | Modern | Canada | -32,7 | -32,1 | Taché and Craig 2015 |
| Non-ruminant | C3 | Otter | *Lontra Canadensis* | Tissue | Modern | Canada | -30,8 | -32,4 | Taché and Craig 2015 |
| Non-ruminant | C3 | Beaver | *Castor canadensis* | Tissue | Modern | Canada | -30,1 | -30,3 | Taché and Craig 2015 |
| Non-ruminant | C3 | Snowshoe hare | *Lepus americanus* | Tissue | Modern | Alaska | -29,4 | -29 | Choy et al. 2016 |
| Non-ruminant | C3 | Red squirrel | *Sciurus vulgaris* | Tissue | Modern | Alaska | -26,2 | -27 | Choy et al. 2016 |
| Non-ruminant | C3 | Mountain hare | *Lepus timidus* | Tissue | Modern | Finland | -32 | -32,3 | Pääkkönen et al. 2020 |
| Non-ruminant | C3 | Mountain hare | *Lepus timidus* | Tissue | Modern | Finland | -31,7 | -32,2 | Pääkkönen et al. 2020 |
| Non-ruminant | C3 | Mountain hare | *Lepus timidus* | Tissue | Modern | Finland | -30,7 | -31,2 | Pääkkönen et al. 2020 |
| Non-ruminant | C3 | Eurasian beaver | *Castor fiber* | Tissue | Modern | Finland | -29,9 | -31 | Pääkkönen et al. 2020 |
| Non-ruminant | C3 | Eurasian beaver | *Castor fiber* | Tissue | Modern | Finland | -27,7 | -28,9 | Pääkkönen et al. 2020 |
| Non-ruminant | C3 | Eurasian beaver | *Castor fiber* | Tissue | Modern | Finland | -28,3 | -28,6 | Pääkkönen et al. 2020 |
| Non-ruminant | C3 | Horse | *Equus caballus* | Tissue | Modern | Kazakhstan | -28,2 | -27,9 | Outram et al. 2009 |
| Non-ruminant | C3 | Horse | *Equus caballus* | Tissue | Modern | Kazakhstan | -28,2 | -28,5 | Outram et al. 2009 |
| Non-ruminant | C3 | Horse | *Equus caballus* | Tissue | Modern | Kazakhstan | -27,7 | -28,6 | Outram et al. 2009 |
| Non-ruminant | C3 | Horse | *Equus caballus* | Tissue | Modern | Kazakhstan | -25,9 | -27 | Outram et al. 2009 |
| Non-ruminant | C3 | Horse | *Equus caballus* | Tissue | Modern | Kazakhstan | -25,6 | -26 | Outram et al. 2009 |
| Non-ruminant | C3 | Raccoon dog | *Nyctereutes procyonoides* | Foodcrust | Modern | Japan | -28,3 | -28,1 | Craig et al. 2013 |
| Non-ruminant | C3 | Raccoon dog | *Nyctereutes procyonoides* | Foodcrust | Modern | Japan | -25,9 | -26,7 | Craig et al. 2013 |
| Non-ruminant | C3 | Horse | *Equus caballus* | Milk | Modern | Kazakhstan | -27,9 | -29 | Outram et al. 2009 |
| Non-ruminant | C3 | Horse | *Equus caballus* | Milk | Modern | Kazakhstan | -27,9 | -28,5 | Outram et al. 2009 |
| Non-ruminant | C3 | Horse | *Equus caballus* | Milk | Modern | Kazakhstan | -27,6 | -27,8 | Outram et al. 2009 |
| Non-ruminant | C3 | Horse | *Equus caballus* | Milk | Modern | Kazakhstan | -27,3 | -28 | Outram et al. 2009 |
| Non-ruminant | C3 | Cattle | *Bos taurus* | Milk | Modern | Kazakhstan | -27,3 | -28,5 | Outram et al. 2009 |
| Non-ruminant | C3 | Pig | *Sus scrofa domesticus* | Soft tissue | Modern | Switzerland | -28,7 | -27,4 | Spangenberg et al. 2006 |
| Non-ruminant | C3 | Pig | *Sus scrofa domesticus* | Soft tissue | Modern | Switzerland | -25,6 | -26,5 | Spangenberg et al. 2006 |
| Non-ruminant | C3 | Wild boar | *Sus scrofa cristatus* | Skin | Modern | Switzerland | -24,4 | -24,4 | Spangenberg et al. 2010 |
| Non-ruminant | C3 | Wild boar | *Sus scrofa ferus* | Tissue | Modern | Finland | -26,4 | -25,5 | Pääkkönen et al. 2020 |
| Non-ruminant | C3 | Wild boar | *Sus scrofa ferus* | Tissue | Modern | Finland | -26,8 | -26,8 | Pääkkönen et al. 2020 |
| Non-ruminant | C3 | Pig | *Sus scrofa domesticus* | Tissue | Modern | Finland | -27,1 | -25,5 | Pääkkönen et al. 2020 |
| Non-ruminant | C3 | Pig | *Sus scrofa domesticus* | Tissue | Modern | Finland | -27,9 | -26,4 | Pääkkönen et al. 2020 |
| Non-ruminant | C3 | Pig | *Sus scrofa domesticus* | Tissue | Modern | Finland | -27,2 | -26,6 | Pääkkönen et al. 2020 |
| Non-ruminant | C3 | Pig | *Sus scrofa domesticus* | Tissue | Modern | Finland | -26,9 | -25,4 | Pääkkönen et al. 2020 |
| Non-ruminant | C3 | Pig | *Sus scrofa domesticus* | Adipose | Modern | UK | -24,8 | -24 | Dudd 1999 |
| Non-ruminant | C3 | Pig | *Sus scrofa domesticus* | Adipose | Modern | UK | -26,4 | -24,8 | Dudd 1999 |
| Non-ruminant | C3 | Pig | Pääkkönen et al. 2020 | Adipose | Modern | UK | -26,5 | -24,8 | Dudd 1999 |
| Non-ruminant | C3 | Pig | *Sus scrofa domesticus* | Adipose | Modern | UK | -25,5 | -24,4 | Dudd 1999 |
| Non-ruminant | C3 | Pig | *Sus scrofa domesticus* | Adipose | Modern | UK | -26 | -24,9 | Dudd 1999 |
| Non-ruminant | C3 | Pig | *Sus scrofa domesticus* | Adipose | Modern | UK | -25,1 | -23,8 | Dudd 1999 |
| Non-ruminant | C3 | Pig | *Sus scrofa domesticus* | Adipose | Modern | UK | -25,7 | -24,4 | Dudd 1999 |
| Non-ruminant | C3 | Pig | *Sus scrofa domesticus* | Adipose | Modern | UK | -25,4 | -24,1 | Dudd 1999 |
| Non-ruminant | C3 | Pig | *Sus scrofa domesticus* | Adipose | Modern | UK | -24,4 | -25,3 | Dudd 1999 |
| Non-ruminant | C3 | Wild boar | *Sus scrofa* | Tissue | Modern | Israel | -28,4 | -26,7 | Gregg et al. 2009 |
| Non-ruminant | C3 | Wild boar | *Sus scrofa* | Tissue | Modern | Israel | -31,4 | -29,7 | Gregg et al. 2009 |
| Non-ruminant | C3 | Wild boar | *Sus scrofa* | Tissue | Modern | Israel | -28,3 | -26,2 | Gregg et al. 2009 |
| Non-ruminant | C3 | Wild boar | *Sus scrofa* | Tissue | Modern | Israel | -29,8 | -25,7 | Gregg et al. 2009 |
| Non-ruminant | C3 | Pig | *Sus scrofa domesticus* | Tissue | Modern | Kazakhstan | -24,7 | -23,8 | Outram et al. 2009 |
| Non-ruminant | C3 | Pig | *Sus scrofa domesticus* | Tissue | Modern | Kazakhstan | -24,7 | -23,6 | Outram et al. 2009 |
| Non-ruminant | C3 | Pig | *Sus scrofa domesticus* | Tissue | Modern | Kazakhstan | -24,4 | -23,8 | Outram et al. 2009 |
| Non-ruminant | C3 | Pig | *Sus scrofa domesticus* | Tissue | Modern | Kazakhstan | -24,3 | -23,5 | Outram et al. 2009 |
| Non-ruminant | C3 | Pig | *Sus scrofa domesticus* | Tissue | Modern | Kazakhstan | -24,1 | -22,9 | Outram et al. 2009 |
| Non-ruminant | C3 | Pig | *Sus scrofa domesticus* | Tissue | Modern | Kazakhstan | -23,9 | -22,8 | Outram et al. 2009 |
| Non-ruminant | C3 | Pig | *Sus scrofa domesticus* | Blood | Modern | Malta | -23,9 | -23,7 | Spiteri 2012 |
| Non-ruminant | C3 | Pig | *Sus scrofa domesticus* | Blood | Modern | Malta | -24,2 | -24,2 | Spiteri 2012 |
| Non-ruminant | C3 | Wild boar | *Sus scrofa* | Adipose | Modern | Germany | -25,1 | -24,8 | Spiteri 2012 |
| Non-ruminant | C3 | Wild boar | *Sus scrofa* | Adipose | Modern | Germany | -28,1 | -28 | Spiteri 2012 |
| Non-ruminant | C3 | Wild boar | *Sus scrofa* | Foodcrust | Modern | Japan | -26,5 | -26,9 | Craig et al. 2013 |
| Non-ruminant | C3 | Wild boar | *Sus scrofa* | Foodcrust | Modern | Japan | -28,1 | -27,7 | Craig et al. 2013 |
| Non-ruminant | C3 | Wild boar | *Sus scrofa* | Adipose | Modern | Japan | -27,4 | -26,3 | Lucquin et al. 2016 |
| Non-ruminant | C3 | Wild boar | *Sus scrofa* | Exp. sherd | Modern | Japan | -27,4 | -26,5 | Lucquin et al. 2016 |
| Non-ruminant | C3 | Wild boar | *Sus scrofa* | Tissue | Modern | Japan | -27,3 | -26,4 | Lucquin et al. 2016 |
| Non-ruminant | C3 | Wild boar | *Sus scrofa* | Tissue | Modern | Japan | -27,7 | -26,7 | Lucquin et al. 2016 |
| Non-ruminant | C3 | Wild boar | *Sus scrofa* | Tissue | Modern | Japan | -27,6 | -26,9 | Lucquin et al. 2016 |
| Non-ruminant | C3 | Wild boar | *Sus scrofa* | Fresh | Modern | Japan | -27,4 | -26,3 | Horiuchi et al. 2015 |
| Non-ruminant | C3 | Wild boar | *Sus scrofa* | Fresh | Modern | Japan | -27,5 | -26,2 | Horiuchi et al. 2015 |
| Non-ruminant | C3 | Wild boar | *Sus scrofa* | Fresh | Modern | Japan | -25,5 | -26 | Horiuchi et al. 2015 |
| Non-ruminant | C3 | Wild boar | *Sus scrofa* | Fresh | Modern | Japan | -27,2 | -26,5 | Horiuchi et al. 2015 |
| Non-ruminant | C3 | Wild boar | *Sus scrofa ferus* |  | Modern | Estonia | -26,7 | -25,6 | Courel et al. 2020 |
| Ruminant adipose | C3 | Sheep | *Ovis aries* |  | Modern | UK | -29,4 | -31,2 | Dudd 1999 |
| Ruminant adipose | C3 | Sheep | *Ovis aries* |  | Modern | UK | -28,4 | -30,1 | Dudd 1999 |
| Ruminant adipose | C3 | Sheep | *Ovis aries* |  | Modern | UK | -28,7 | -30,4 | Dudd 1999 |
| Ruminant adipose | C3 | Sheep | *Ovis aries* |  | Modern | UK | -29,2 | -31,1 | Dudd 1999 |
| Ruminant adipose | C3 | Sheep | *Ovis aries* |  | Modern | UK | -29,3 | -31,1 | Dudd 1999 |
| Ruminant adipose | C3 | Sheep | *Ovis aries* |  | Modern | UK | -28,4 | -31,3 | Dudd 1999 |
| Ruminant adipose | C3 | Sheep | *Ovis aries* |  | Modern | UK | -28,8 | -30,1 | Dudd 1999 |
| Ruminant adipose | C3 | Sheep | *Ovis aries* |  | Modern | UK | -28,2 | -30 | Dudd 1999 |
| Ruminant adipose | C3 | Sheep | *Ovis aries* |  | Modern | UK | -30,4 | -32,2 | Dudd 1999 |
| Ruminant adipose | C3 | Sheep | *Ovis aries* |  | Modern | UK | -30,2 | -32,3 | Dudd 1999 |
| Ruminant adipose | C3 | Sheep | *Ovis aries* |  | Modern | UK | -30,5 | -32,5 | Dudd 1999 |
| Ruminant adipose | C3 | Sheep | *Ovis aries* |  | Modern | UK | -29,4 | -31,8 | Dudd 1999 |
| Ruminant adipose | C3 | Sheep | *Ovis aries* |  | Modern | UK | -29 | -30,4 | Dudd 1999 |
| Ruminant adipose | C3 | Cow | *Bos taurus* |  | Modern | UK | -28,9 | -31,8 | Dudd 1999 |
| Ruminant adipose | C3 | Cow | *Bos taurus* |  | Modern | UK | -29,8 | -32,3 | Dudd 1999 |
| Ruminant adipose | C3 | Cow | *Bos taurus* |  | Modern | UK | -28,7 | -31,5 | Dudd 1999 |
| Ruminant adipose | C3 | Cow | *Bos taurus* |  | Modern | UK | -29,9 | -31,6 | Dudd 1999 |
| Ruminant adipose | C3 | Cattle | *Bos taurus* | Tissue | Modern | Switzerland | -27,1 | -28,6 | Spangenberg et al. 2006 |
| Ruminant adipose | C3 | Cattle | *Bos taurus* | Tissue | Modern | Switzerland | -27,8 | -28,3 | Spangenberg et al. 2006 |
| Ruminant adipose | C3 | Cattle | *Bos taurus* | Tissue | Modern | Switzerland | -31,4 | -31,3 | Spangenberg et al. 2006 |
| Ruminant adipose | C3 | Cattle | *Bos taurus* | Tissue | Modern | Switzerland | -30 | -30,2 | Spangenberg et al. 2006 |
| Ruminant adipose | C3 | Sheep | *Ovis aries* | Tissue | Modern | Switzerland | -27,7 | -26,4 | Spangenberg et al. 2006 |
| Ruminant adipose | C3 | Cattle | *Bos taurus* | Skin | Modern | Switzerland | -25,3 | -26,3 | Spangenberg et al. 2010 |
| Ruminant adipose | C3 | Cow | *Bos taurus* | Skin | Modern | Switzerland | -26,1 | -25,3 | Spangenberg et al. 2010 |
| Ruminant adipose | C3 | Sheep | *Ovis aries* | Skin | Modern | Switzerland | -27,2 | -28,3 | Spangenberg et al. 2010 |
| Ruminant adipose | C3 | Cow | *Bos taurus* | Skin | Modern | Switzerland | -25,4 | -26,1 | Spangenberg et al. 2010 |
| Ruminant adipose | C3 | Goat | *Capra hircus* | Skin | Modern | Switzerland | -28,4 | -30 | Spangenberg et al. 2010 |
| Ruminant adipose | C3 | Goat | *Capra hircus* | Skin | Modern | Switzerland | -28,3 | -30 | Spangenberg et al. 2010 |
| Ruminant adipose | C3 | Chamois | *Rupicapra rupicapra* | Skin | Modern | Switzerland | -26,3 | -25,7 | Spangenberg et al. 2010 |
| Ruminant adipose | C3 | Cattle | *Bos taurus* | Tissue | Modern | Finland | -27,4 | -28,8 | Pääkkönen et al. 2020 |
| Ruminant adipose | C3 | Cattle | *Bos taurus* | Tissue | Modern | Finland | -26,7 | -29,2 | Pääkkönen et al. 2020 |
| Ruminant adipose | C3 | Cattle | *Bos taurus* | Tissue | Modern | Finland | -27,5 | -29,5 | Pääkkönen et al. 2020 |
| Ruminant adipose | C3 | Cattle | *Bos taurus* | Tissue | Modern | Finland | -27 | -28,5 | Pääkkönen et al. 2020 |
| Ruminant adipose | C3 | Cattle | *Bos taurus* |  | Modern | Estonia | -27,7 | -29,9 | Courel et al. 2020 |
| Ruminant adipose | C3 | Sheep | *Ovis aries* |  | Modern | Estonia | -30 | -31,6 | Courel et al. 2020 |
| Ruminant adipose | Mixed C3/C4 | Sheep | *Ovis aries* | Tissue | Modern | Israel | -23,5 | -23,4 | Gregg et al. 2009 |
| Ruminant adipose | Mixed C3/C4 | Goat | *Capra hircus* | Tissue | Modern | Israel | -29,3 | -29,8 | Gregg et al. 2009 |
| Ruminant adipose | Mixed C3/C4 | Goat | *Capra hircus* | Tissue | Modern | Israel | -29,5 | -29,7 | Gregg et al. 2009 |
| Ruminant adipose | Mixed C3/C4 | Cow | *Bos taurus* | *Adipose* | Modern | Malta | -22 | -22,3 | Spiteri 2012 |
| Ruminant adipose | Mixed C3/C4 | Sheep | *Ovis aries* | *Adipose* | Modern | Malta | -21,2 | -22 | Spiteri 2012 |
| Ruminant adipose | Mixed C3/C4 | Goat | *Capra hircus* | *Adipose* | Modern | Malta | -17,9 | -18,5 | Spiteri 2012 |
| Ruminant adipose | Mixed C3/C4 | Cattle/sheep/goat |  | Adipose | Modern | Kenya | -28,9 | -30,4 | Dunne et al 2012 |
| Ruminant adipose | Mixed C3/C4 | Cattle/sheep/goat |  | Adipose | Modern | Kenya | -28,3 | -29,8 | Dunne et al 2012 |
| Ruminant adipose | Mixed C3/C4 | Cattle/sheep/goat |  | Adipose | Modern | Kenya | -28,4 | -29,1 | Dunne et al 2012 |
| Ruminant adipose | Mixed C3/C4 | Cattle/sheep/goat |  | Adipose | Modern | Kenya | -28,4 | -28,6 | Dunne et al 2012 |
| Ruminant adipose | Mixed C3/C4 | Cattle/sheep/goat |  | Adipose | Modern | Kenya | -26,8 | -26,7 | Dunne et al 2012 |
| Ruminant adipose | Mixed C3/C4 | Cattle/sheep/goat |  | Adipose | Modern | Kenya | -26,5 | -27,1 | Dunne et al 2012 |
| Ruminant adipose | Mixed C3/C4 | Cattle/sheep/goat |  | Adipose | Modern | Kenya | -25,9 | -27,1 | Dunne et al 2012 |
| Ruminant adipose | Mixed C3/C4 | Cattle/sheep/goat |  | Adipose | Modern | Kenya | -25,7 | -27,1 | Dunne et al 2012 |
| Ruminant adipose | Mixed C3/C4 | Cattle/sheep/goat |  | Adipose | Modern | Kenya | -23,7 | -24,6 | Dunne et al 2012 |
| Ruminant adipose | Mixed C3/C4 | Cattle/sheep/goat |  | Adipose | Modern | Kenya | -23,4 | -24,7 | Dunne et al 2012 |
| Ruminant adipose | Mixed C3/C4 | Cattle/sheep/goat |  | Adipose | Modern | Kenya | -22,8 | -24,4 | Dunne et al 2012 |
| Ruminant adipose | Mixed C3/C4 | Cattle/sheep/goat |  | Adipose | Modern | Kenya | -20,3 | -21,6 | Dunne et al 2012 |
| Ruminant adipose | Mixed C3/C4 | Cattle/sheep/goat |  | Adipose | Modern | Kenya | -19,5 | -21,7 | Dunne et al 2012 |
| Ruminant adipose | Mixed C3/C4 | Cattle/sheep/goat |  | Adipose | Modern | Kenya | -20 | -21,2 | Dunne et al 2012 |
| Ruminant adipose | Mixed C3/C4 | Cattle/sheep/goat |  | Adipose | Modern | Kenya | -19,9 | -21 | Dunne et al 2012 |
| Ruminant adipose | Mixed C3/C4 | Cattle/sheep/goat |  | Adipose | Modern | Kenya | -18 | -19,9 | Dunne et al 2012 |
| Ruminant adipose | Mixed C3/C4 | Cattle/sheep/goat |  | Adipose | Modern | Kenya | -17,4 | -20 | Dunne et al 2012 |
| Ruminant adipose | Mixed C3/C4 | Cattle/sheep/goat |  | Adipose | Modern | Kenya | -17 | -20,3 | Dunne et al 2012 |
| Ruminant adipose | Mixed C3/C4 | Cattle/sheep/goat |  | Adipose | Modern | Kenya | -17,1 | -18,6 | Dunne et al 2012 |
| Ruminant adipose | Mixed C3/C4 | Cattle/sheep/goat |  | Adipose | Modern | Kenya | -16,4 | -18,2 | Dunne et al 2012 |
| Ruminant adipose | Mixed C3/C4 | Cattle/sheep/goat |  | Adipose | Modern | Kenya | -15,7 | -18,4 | Dunne et al 2012 |
| Ruminant adipose | Mixed C3/C4 | Cattle/sheep/goat |  | Adipose | Modern | Kenya | -16 | -18,9 | Dunne et al 2012 |
| Ruminant adipose | Mixed C3/C4 | Cattle/sheep/goat |  | Adipose | Modern | Kenya | -15,5 | -19 | Dunne et al 2012 |
| Ruminant adipose | Mixed C3/C4 | Cattle/sheep/goat |  | Adipose | Modern | Kenya | -15,6 | -18,3 | Dunne et al 2012 |
| Ruminant adipose | Mixed C3/C4 | Cattle/sheep/goat |  | Adipose | Modern | Kenya | -15,8 | -18,5 | Dunne et al 2012 |
| Ruminant adipose | Mixed C3/C4 | Cattle/sheep/goat |  | Adipose | Modern | Kenya | -15,8 | -17,8 | Dunne et al 2012 |
| Ruminant adipose | Mixed C3/C4 | Cattle/sheep/goat |  | Adipose | Modern | Kenya | -15,7 | -18,2 | Dunne et al 2012 |
| Ruminant adipose | Mixed C3/C4 | Cattle/sheep/goat |  | Adipose | Modern | Kenya | -14,9 | -18,4 | Dunne et al 2012 |
| Ruminant adipose | Mixed C3/C4 | Cattle/sheep/goat |  | Adipose | Modern | Kenya | -15,7 | -17,2 | Dunne et al 2012 |
| Ruminant adipose | Mixed C3/C4 | Cattle/sheep/goat |  | Adipose | Modern | Kenya | -15 | -17,4 | Dunne et al 2012 |
| Ruminant adipose | Mixed C3/C4 | Cattle/sheep/goat |  | Adipose | Modern | Kenya | -14,4 | -17,3 | Dunne et al 2012 |
| Ruminant dairy | C3 | Cow | *Bos taurus* | Milk | Modern | UK | -30,8 | -34,4 | Dudd 1999 |
| Ruminant dairy | C3 | Cow | *Bos taurus* | Milk | Modern | UK | -27,8 | -32,1 | Dudd 1999 |
| Ruminant dairy | C3 | Sheep | *Ovis aries* | Milk | Modern | UK | -29,4 | -33,8 | Dudd 1999 |
| Ruminant dairy | C3 | Sheep | *Ovis aries* | Milk | Modern | UK | -29 | -33,4 | Dudd 1999 |
| Ruminant dairy | C3 | Cow | *Bos taurus* | Milk | Modern | UK | -27,4 | -32,2 | Dudd 1999 |
| Ruminant dairy | C3 | Cow | *Bos taurus* | Milk | Modern | UK | -28,9 | -33,7 | Dudd 1999 |
| Ruminant dairy | C3 | Cow | *Bos taurus* | Milk | Modern | UK | -29,6 | -34,9 | Dudd 1999 |
| Ruminant dairy | C3 | Cow | *Bos taurus* | Milk | Modern | UK | -27,9 | -33,1 | Dudd 1999 |
| Ruminant dairy | C3 | Cow | *Bos taurus* | Milk | Modern | UK | -28,6 | -34,1 | Dudd 1999 |
| Ruminant dairy | C3 | Cow | *Bos taurus* | Milk | Modern | UK | -28,1 | -34 | Dudd 1999 |
| Ruminant dairy | C3 | Cattle | *Bos taurus* | Cheese | Modern | Switzerland | -27,3 | -31,9 | Spangenberg et al. 2006 |
| Ruminant dairy | C3 | Cattle | *Bos taurus* | Milk | Modern | Switzerland | -28,5 | -33,5 | Spangenberg et al. 2006 |
| Ruminant dairy | C3 | Cattle | *Bos taurus* | Milk | Modern | Switzerland | -28,1 | -32,9 | Spangenberg et al. 2006 |
| Ruminant dairy | C3 | Cattle | *Bos taurus* | Milk | Modern | Switzerland | -27,5 | -32,9 | Spangenberg et al. 2006 |
| Ruminant dairy | C3 | Cattle | *Bos taurus* | Milk | Modern | Switzerland | -27,8 | -31,7 | Spangenberg et al. 2006 |
| Ruminant dairy | C3 | Cattle | *Bos taurus* | Milk | Modern | Switzerland | -26,9 | -31 | Spangenberg et al. 2006 |
| Ruminant dairy | C3 | Cattle | *Bos taurus* | Milk | Modern | Switzerland | -27,5 | -30,7 | Spangenberg et al. 2006 |
| Ruminant dairy | C3 | Cattle | *Bos taurus* | Milk | Modern | Switzerland | -27,1 | -29,9 | Spangenberg et al. 2006 |
| Ruminant dairy | C3 | Cattle | *Bos taurus* | Milk | Modern | Switzerland | -28,5 | -31 | Spangenberg et al. 2006 |
| Ruminant dairy | C3 | Cattle | *Bos taurus* | Milk | Modern | Switzerland | -25,6 | -32,2 | Spangenberg et al. 2006 |
| Ruminant dairy | C3 | Goat | *Capra hircus* | Cheese | Modern | Switzerland | -24,8 | -28,7 | Spangenberg et al. 2006 |
| Ruminant dairy | C3 | Goat | *Capra hircus* | Cheese | Modern | Switzerland | -24,9 | -27,6 | Spangenberg et al. 2006 |
| Ruminant dairy | C3 | Goat | *Capra hircus* | Milk | Modern | Switzerland | -26,2 | -33,3 | Spangenberg et al. 2006 |
| Ruminant dairy | C3 | Goat | *Capra hircus* | Milk | Modern | Switzerland | -25,5 | -32,4 | Spangenberg et al. 2006 |
| Ruminant dairy | C3 | Goat | *Capra hircus* | Milk | Modern | Switzerland | -28,2 | -31,1 | Spangenberg et al. 2006 |
| Ruminant dairy | C3 | Sheep | *Ovis aries* | Cheese | Modern | Switzerland | -29 | -33,9 | Spangenberg et al. 2006 |
| Ruminant dairy | C3 | Sheep | *Ovis aries* | Cheese | Modern | Switzerland | -28 | -33,4 | Spangenberg et al. 2006 |
| Ruminant dairy | C3 | Sheep | *Ovis aries* | Cheese | Modern | Switzerland | -30,8 | -33,2 | Spangenberg et al. 2006 |
| Ruminant dairy | C3 | Sheep | *Ovis aries* | Milk | Modern | Switzerland | -31,4 | -37 | Spangenberg et al. 2006 |
| Ruminant dairy | C3 | Sheep | *Ovis aries* | Milk | Modern | Switzerland | -31,3 | -38 | Spangenberg et al. 2006 |
| Ruminant dairy | C3 | Sheep | *Ovis aries* | Milk | Modern | Switzerland | -30,9 | -38,3 | Spangenberg et al. 2006 |
| Ruminant dairy | C3 | Sheep | *Ovis aries* | Milk | Modern | Switzerland | -32 | -39,2 | Spangenberg et al. 2006 |
| Ruminant dairy | C3 | Cattle | *Bos taurus* | Milk | Modern | Finland | -27,1 | -30,7 | Pääkkönen et al. 2020 |
| Ruminant dairy | C3 | Cattle | *Bos taurus* | Milk | Modern | Finland | -26,8 | -30,7 | Pääkkönen et al. 2020 |
| Ruminant dairy | C3 | Cattle | *Bos taurus* | Milk | Modern | Finland | -26,8 | -31,5 | Pääkkönen et al. 2020 |
| Ruminant dairy | C3 | Cattle | *Bos taurus* | Milk | Modern | Finland | -26,6 | -31,2 | Pääkkönen et al. 2020 |
| Ruminant dairy | C3 | Cattle | *Bos taurus* | Milk | Modern | Finland | -27,5 | -31,6 | Pääkkönen et al. 2020 |
| Ruminant dairy | C3 | Goat | *Capra hircus* | Milk | Modern | Finland | -25,4 | -29,6 | Pääkkönen et al. 2020 |
| Ruminant dairy | C3 | Goat | *Capra hircus* | Milk | Modern | Finland | -27,2 | -30,9 | Pääkkönen et al. 2020 |
| Ruminant dairy | C3 | Goat | *Capra hircus* | Milk | Modern | Finland | -27,2 | -32,3 | Pääkkönen et al. 2020 |
| Ruminant dairy | C3 | Goat | *Capra hircus* | Milk | Modern | Finland | -27,6 | -31,5 | Pääkkönen et al. 2020 |
| Ruminant dairy | C3 | Cattle | *Bos taurus* | Milk | Modern | Estonia | -25,6 | -34,3 | Courel et al. 2020 |
| Ruminant dairy | C3 | Cow | *Bos taurus* | Milk | Modern | Malta | -23,9 | -26,3 | Spiteri 2012 |
| Ruminant dairy | C3 | Cow | *Bos taurus* | Milk | Modern | Malta | -23,3 | -26,5 | Spiteri 2012 |
| Ruminant dairy | C3 | Sheep | *Ovis aries* | Milk | Modern | Malta | -24,4 | -29 | Spiteri 2012 |
| Ruminant dairy | C3 | Sheep | *Ovis aries* | Milk | Modern | Malta | -24,2 | -28,5 | Spiteri 2012 |
| Ruminant dairy | C3 | Goat | *Capra hircus* | Milk | Modern | Malta | -24,9 | -28,1 | Spiteri 2012 |
| Ruminant dairy | C3 | Goat | *Capra hircus* | Milk | Modern | Malta | -23,4 | -26,4 | Spiteri 2012 |
| Ruminant dairy | C3 | Cow | *Bos taurus* | Milk | Modern | Germany | -27 | -30,8 | Spiteri 2012 |
| Ruminant dairy | Mixed C3/C4 | Sheep | *Ovis aries* | Butter | Modern | Jordan | -28 | -32,3 | Gregg et al. 2009 |
| Ruminant dairy | Mixed C3/C4 | Sheep | *Ovis aries* | Milk | Modern | Malta | -21,6 | -25,9 | Spiteri 2012 |
| Ruminant dairy | Mixed C3/C4 | Goat | *Capra hircus* | Milk | Modern | Malta | -19,3 | -26,4 | Spiteri 2012 |
| Ruminant dairy | Mixed C3/C4 | Sheep | *Ovis aries* | Cheese | Modern | Italy | -22,5 | -27,6 | Spiteri 2012 |
| Ruminant dairy | Mixed C3/C4 | Sheep | *Ovis aries* | Cheese | Modern | Italy | -21,3 | -26,7 | Spiteri 2012 |
| Ruminant dairy | Mixed C3/C4 | Goat | *Capra hircus* | Dairy | Modern | Libya, | -27,7 | -32,1 | Dunne et al 2012 |
| Ruminant dairy | Mixed C3/C4 | Goat | *Capra hircus* | Dairy | Modern | Libya | -26,7 | -31,1 | Dunne et al 2012 |
| Ruminant dairy | Mixed C3/C4 | Goat | *Capra hircus* | Dairy | Modern | Libya | -25,6 | -31,2 | Dunne et al 2012 |
| Ruminant dairy | Mixed C3/C4 | Goat | *Capra hircus* | Dairy | Modern | Libya | -25,7 | -30,8 | Dunne et al 2012 |
| Ruminant dairy | Mixed C3/C4 | Goat | *Capra hircus* | Dairy | Modern | Libya | -24,8 | -30,8 | Dunne et al 2012 |
| Ruminant dairy | Mixed C3/C4 | Goat | *Capra hircus* | Dairy | Modern | Libya | -26,5 | -29,7 | Dunne et al 2012 |
| Ruminant dairy | Mixed C3/C4 | Goat | *Capra hircus* | Dairy | Modern | Libya | -25,9 | -29,6 | Dunne et al 2012 |
| Ruminant dairy | Mixed C3/C4 | Goat | *Capra hircus* | Dairy | Modern | Libya | -23,5 | -29,7 | Dunne et al 2012 |
| Ruminant dairy | Mixed C3/C4 | Goat | *Capra hircus* | Dairy | Modern | Libya | -23,6 | -29,5 | Dunne et al 2012 |
| Ruminant dairy | Mixed C3/C4 | Cattle | *Bos taurus* | Dairy | Modern | Kenya | -24,5 | -29,3 | Dunne et al 2012 |
| Ruminant dairy | Mixed C3/C4 | Cattle | *Bos taurus* | Dairy | Modern | Kenya | -24 | -28,6 | Dunne et al 2012 |
| Ruminant dairy | Mixed C3/C4 | Cattle | *Bos taurus* | Dairy | Modern | Kenya | -25,7 | -28,7 | Dunne et al 2012 |
| Ruminant dairy | Mixed C3/C4 | Cattle | *Bos taurus* | Dairy | Modern | Kenya | -25,4 | -28,4 | Dunne et al 2012 |
| Ruminant dairy | Mixed C3/C4 | Cattle | *Bos taurus* | Dairy | Modern | Kenya | -17,3 | -23,1 | Dunne et al 2012 |
| Ruminant dairy | Mixed C3/C4 | Cattle | *Bos taurus* | Dairy | Modern | Kenya | -17,4 | -22,7 | Dunne et al 2012 |
| Ruminant dairy | Mixed C3/C4 | Cattle | *Bos taurus* | Dairy | Modern | Kenya | -14,7 | -20,9 | Dunne et al 2012 |
| Ruminant dairy | Mixed C3/C4 | Cattle | *Bos taurus* | Dairy | Modern | Kenya | -14,8 | -21,1 | Dunne et al 2012 |
| Ruminant dairy | Mixed C3/C4 | Cattle | *Bos taurus* | Dairy | Modern | Kenya | -14,6 | -21,1 | Dunne et al 2012 |
| Ruminant dairy | Mixed C3/C4 | Cattle | *Bos taurus* | Milk | Modern | Estonia | -25,6 | -34,3 | Courel et al. 2020 |
| Ruminant dairy | Mixed C3/C4 | Cattle | *Bos taurus* | Milk | Modern | Kazakhstan | -27,7 | -30,4 | Outram et al. 2009 |
| Ruminant dairy | Mixed C3/C4 | Cattle | *Bos taurus* | Milk | Modern | Kazakhstan | -26,9 | -30 | Outram et al. 2009 |
| Ruminant dairy | Mixed C3/C4 | Cattle | *Bos taurus* | Milk | Modern | Kazakhstan | -25,7 | -29,5 | Outram et al. 2009 |
| Ruminant dairy | Mixed C3/C4 | Cattle | *Bos taurus* | Milk | Modern | Kazakhstan | -25,1 | -29,6 | Outram et al. 2009 |

**References**

Choy, Kyungcheol, Ben A. Potter, Holly J. McKinney, Joshua D. Reuther, Shiway W. Wang, and Matthew J. Wooller

2016 Chemical profiling of ancient hearths reveals recurrent salmon use in Ice Age Beringia. *Proceedings of the National Academy of Sciences* 113(35):9757–9762. DOI:[10.1073/pnas.1606219113](https://doi.org/10.1073/pnas.1606219113)

Colonese, André C., Thomas Farrell, Alexandre Lucquin, Daniel Firth, Sophy Charlton, Harry K. Robson, Michelle Alexander, and Oliver E. Craig

2015 Archaeological bone lipids as palaeodietary markers: Lipids as dietary markers. *Rapid Communications in Mass Spectrometry* 29(7):611–618. DOI:[10.1002/rcm.7144](https://doi.org/10.1002/rcm.7144).

Courel, Blandine, Harry K. Robson, Alexandre Lucquin, Ekaterina Dolbunova, Ester Oras, Kamil Adamczak, Søren H. Andersen, Peter Moe Astrup, Maxim Charniauski, Agnieszka Czekaj-Zastawny, Igor Ezepenko, Sönke Hartz, Jacek Kabaciński, Andreas Kotula, Stanisław Kukawka, Ilze Loze, Andrey Mazurkevich, Henny Piezonka, Gytis Piličiauskas, Søren A. Sørensen, Helen M. Talbot, Aleh Tkachou, Maryia Tkachova, Adam Wawrusiewicz, John Meadows, Carl P. Heron, and Oliver E. Craig

2020 Organic residue analysis shows sub-regional patterns in the use of pottery by Northern European hunter–gatherers. *Royal Society Open Science* 7(4):192016. DOI:[10.1098/rsos.192016](https://doi.org/10.1098/rsos.192016).

Craig, Oliver E., Richard B. Allen, Anu Thompson, Rhiannon E. Stevens, Valerie J. Steele, and Carl Heron

2012 Distinguishing wild ruminant lipids by gas chromatography/combustion/isotope ratio mass spectrometry: Distinguishing ruminant lipids. *Rapid Communications in Mass Spectrometry* 26(19):2359–2364. DOI:[10.1002/rcm.6349](https://doi.org/10.1002/rcm.6349).

Craig, O. E., H. Saul, A. Lucquin, Y. Nishida, K. Taché, L. Clarke, A. Thompson, D. T. Altoft, J. Uchiyama, M. Ajimoto, K. Gibbs, S. Isaksson, C. P. Heron, and P. Jordan

2013 Earliest evidence for the use of pottery. *Nature* 496(7445):351–354. DOI:[10.1038/nature12109](https://doi.org/10.1038/nature12109).

Dudd, Stephanie N.

1999 *Molecular and Isotopic Characterisation of Animal Fats in Archaeological Pottery*. Unpublished PhD thesis, University of Bristol.

Dunne, Julie, Richard P. Evershed, Mélanie Salque, Lucy Cramp, Silvia Bruni, Kathleen Ryan, Stefano Biagetti, and Savino di Lernia

2012 First dairying in green Saharan Africa in the fifth millennium bc. *Nature* 486(7403):390–394. DOI:[10.1038/nature11186](https://doi.org/10.1038/nature11186).

Gregg, M.W., E.B. Banning, K. Gibbs, and G.F. Slater

2009 Subsistence practices and pottery use in Neolithic Jordan: molecular and isotopic evidence. *Journal of Archaeological Science* 36(4):937–946. DOI:[10.1016/j.jas.2008.09.009](https://doi.org/10.1016/j.jas.2008.09.009).

Horiuchi, Akiko, Yoshiki Miyata, Nobuhiko Kamijo, Lucy Cramp, and Richard P Evershed

2015 A Dietary Study of the Kamegaoka Culture Population during the Final Jomon Period, Japan, Using Stable Isotope and Lipid Analyses of Ceramic Residues. *Radiocarbon* 57(4):721–736. DOI:[10.2458/azu_rc.57.18455](https://doi.org/10.2458/azu_rc.57.18455).

Lucquin, Alexandre, Kevin Gibbs, Junzo Uchiyama, Hayley Saul, Mayumi Ajimoto, Yvette Eley, Anita Radini, Carl P. Heron, Shinya Shoda, Yastami Nishida, Jasmine Lundy, Peter Jordan, Sven Isaksson, and Oliver E. Craig

2016 Ancient lipids document continuity in the use of early hunter–gatherer pottery through 9,000 years of Japanese prehistory. *Proceedings of the National Academy of Sciences* 113(15):3991–3996. DOI:[10.1073/pnas.1522908113](https://doi.org/10.1073/pnas.1522908113).

March, Ramiro J.

2013 Searching for the Functions of Fire Structures in Eynan (Mallaha) and their Formation Processes: A Geochemical Approach, in O. Bar-Yosef and F.R. Valla (eds.), *Natufian Foragers in the Levant: Terminal Pleistocene Social Changes in Western Asia*, pp. 227-223. Archaeological Series. International Monographs in Prehistory, Ann Arbor.

Outram, Alan K., Natalie A. Stear, Robin Bendrey, Sandra Olsen, Alexei Kasparov, Victor Zaibert, Nick Thorpe, and Richard P. Evershed

2009 The Earliest Horse Harnessing and Milking. *Science, New Series* 323(5919):1332–1335.

Pääkkönen, Mirva, Richard P. Evershed, and Henrik Asplund

2020

Compound-specific stable carbon isotope values of fatty acids in modern aquatic and terrestrial animals from the Baltic Sea and Finland as an aid to interpretations of the origins of organic residues preserved in archaeological pottery. *Journal of Nordic Archaeological Science* 19.

Spangenberg, Jorge E., Stefanie Jacomet, and Jörg Schibler

2006 Chemical analyses of organic residues in archaeological pottery from Arbon Bleiche 3, Switzerland – evidence for dairying in the late Neolithic. *Journal of Archaeological Science* 33(1):1–13. DOI:[10.1016/j.jas.2005.05.013](https://doi.org/10.1016/j.jas.2005.05.013).

Spangenberg, Jorge E., Montserrat Ferrer, Pascal Tschudin, Marquita Volken, and Albert Hafner

2010 Microstructural, chemical and isotopic evidence for the origin of late neolithic leather recovered from an ice field in the Swiss Alps. *Journal of Archaeological Science* 37(8):1851–1865. DOI:[10.1016/j.jas.2010.02.003](https://doi.org/10.1016/j.jas.2010.02.003).

Spiteri, Cynthianne Debono

2012 Pottery Use at the Transition to Agriculture in the Western Mediterranean. Evidence from Biomolecular and Isotopic Characterisation of Organic Residues in Impressed/Cardial Ware Vessels. Unpublished Unpublished PhD thesis, University of York.

Taché, Karine, and Oliver E. Craig

2015 Cooperative harvesting of aquatic resources and the beginning of pottery production in north-eastern North America. *Antiquity* 89(343):177–190. DOI:[10.15184/aqy.2014.36](https://doi.org/10.15184/aqy.2014.36).
