# Supplementary material for: What do “barbarians” eat? Integrating ceramic use-wear and residue analysis in the study of food and society at the margins of Bronze Age China
Source: PLoS One. 2021 Apr 29;16(4):e0250819. doi: 10.1371/journal.pone.0250819 (PMC8084173; doi:10.1371/journal.pone.0250819)
Supplement: S2 File — (DOCX) [file pone.0250819.s002.docx]

S2 – Cooking experimentation

A total of six cooking experiments were performed at the NYU test kitchen on March 3^rd^ 2018. Chef Raymond Childs performed the cooking experiment with Dr. Yitzchak Jaffe. Two medium sized La Chamba black unglazed pots ( 4 qt =3.7 L) were used. The pots were chosen as they are still made in traditional fashion (see [here](https://ancientcookware.com/la-chamba-collection/black-clay-la-chamba-rounded-soup-pot-detail)) and have a shape similar to the Siwa *ma’an* double handled jar (Miller et al. 2020 are using this same pot in a recent study). Below we describe the results of each of the 6 experiments aimed at recreating the usewear pattern observed on the *ma’an* jars. Pots were scrubbed and washed between each experiment so as to avoid mistakenly documenting unrelated use wear accumulations (carbonization/water lines etc.). We were unable to ‘clean’ external oxidation marks, which accumulated almost evenly when exposed to flames from the vessel side, thus using two separate pots helped with this.

1. Millet flour porridge (made with ground millet and water)

Millet flour and water were boiled together by exposing the pot, base down, to flames on one side (but not on bottom (figure1a). This was reduced for almost an hour until it reached a soupy gruel with lumps of millet (figure 1b).

| 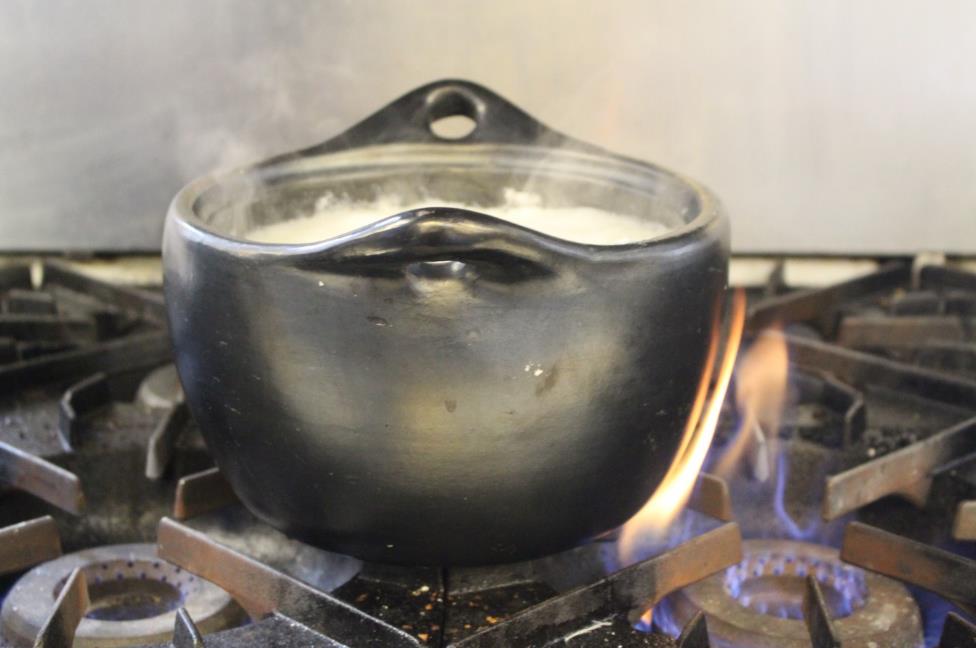 | 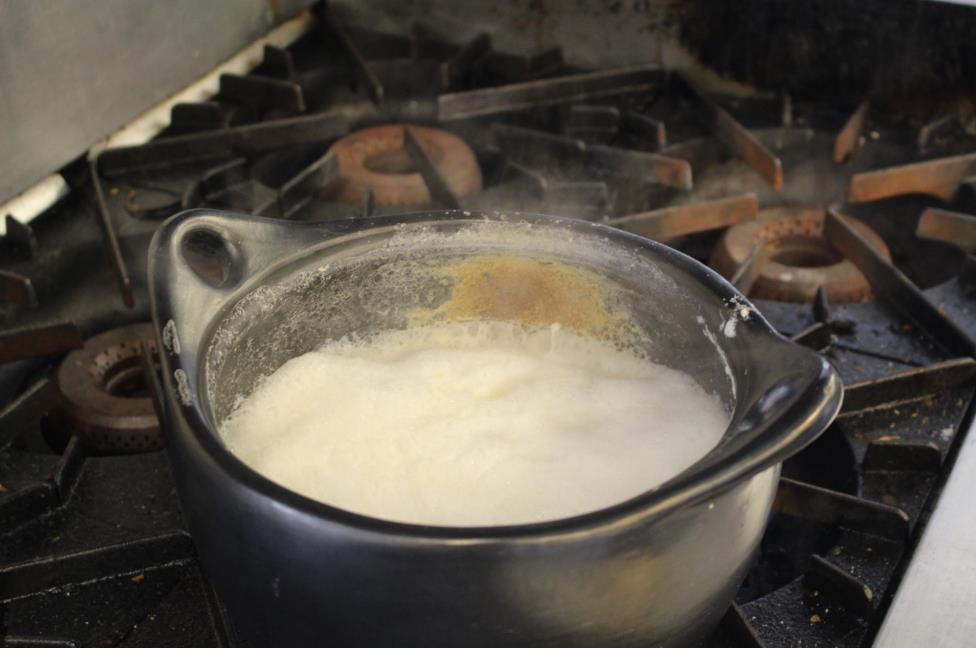 |
| --- | --- |

figures 1a figure 1b

A water line formed as the gruel reduced (figure 1c). Additional internal carbonization was clearly visible up from the base (and including the base) to just under the water line (figures 1d&1e). Externally, a red oxidation patch, reminiscent of the horse-shaped pattern observed on *ma’an* vessels, was created (figure 1f).

| 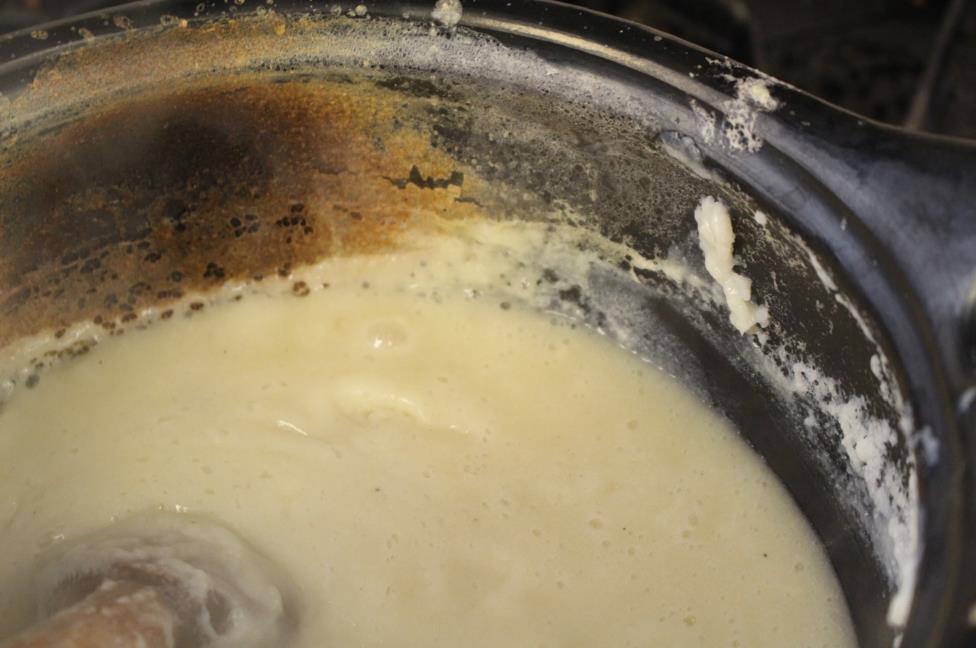 | 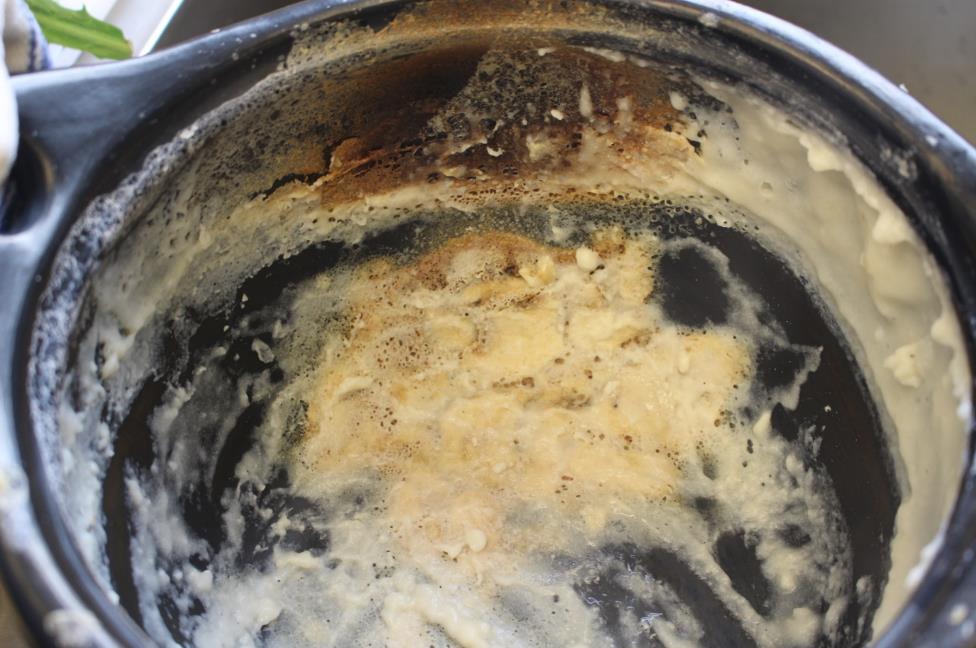 |
| --- | --- |

Figure 1c figure 1d

| 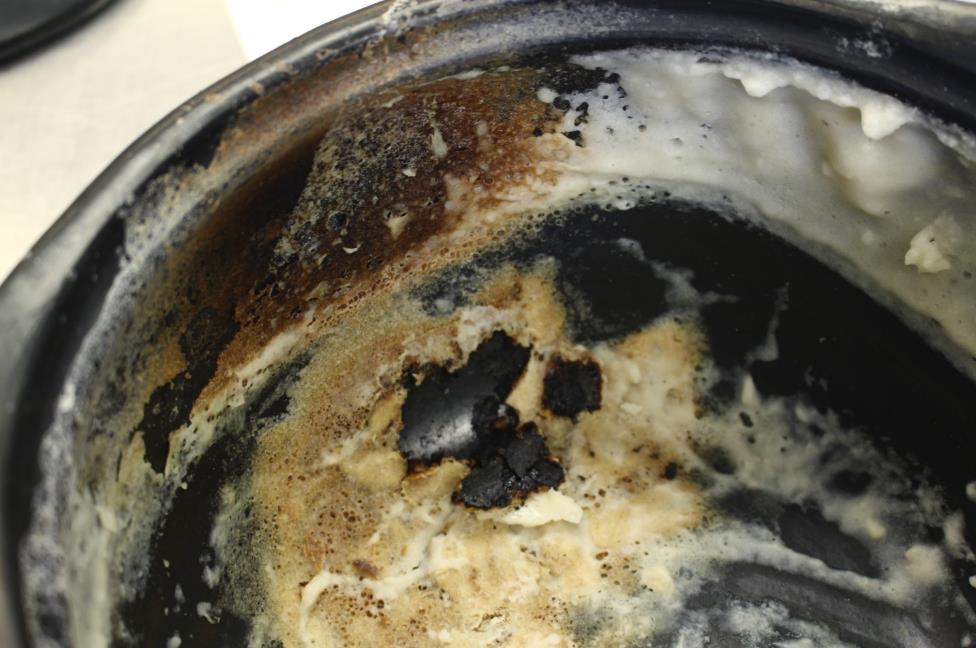 | 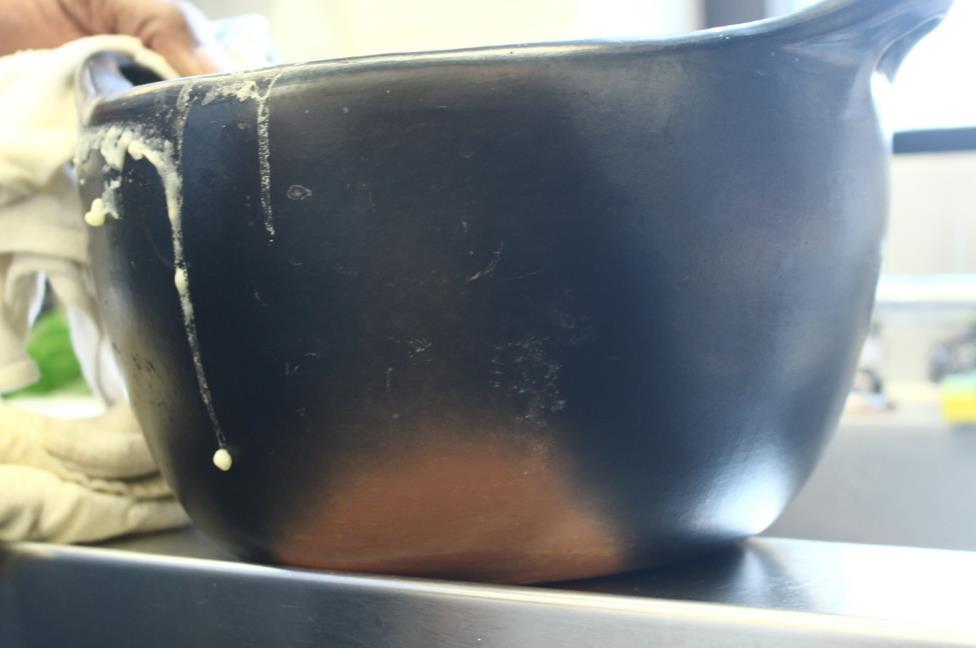 |
| --- | --- |

Figure 1e figure 1f

1. Millet flour noodles boiled in water

Millet flour was combined with water to create dough formed and shaped into noodles (figures 2a&b). Lu et al. (2005) reported ~4000 BP noodles from the relatively nearby site of Lajia, but have been contested by some (see in Hu et al 2014). Noodles were placed in lukewarm water to prevent falling apart. Noodles were ready in a few minutes after boiling (~25 minutes; figure 2c). A water line was formed by this cooking experiment, and while some residue is found above it, it flaked and washed off (figure 2d-f).

| 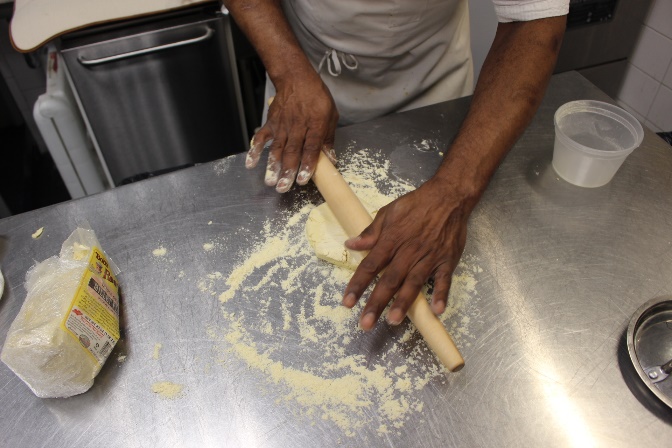 | 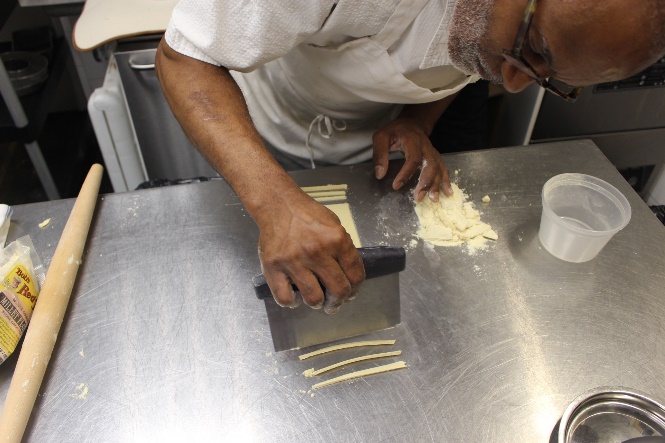 |
| --- | --- |

Figure 2a figure 2b

| 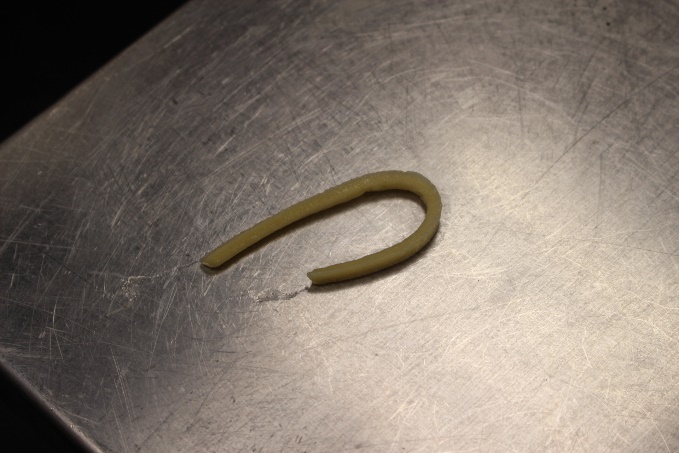 | 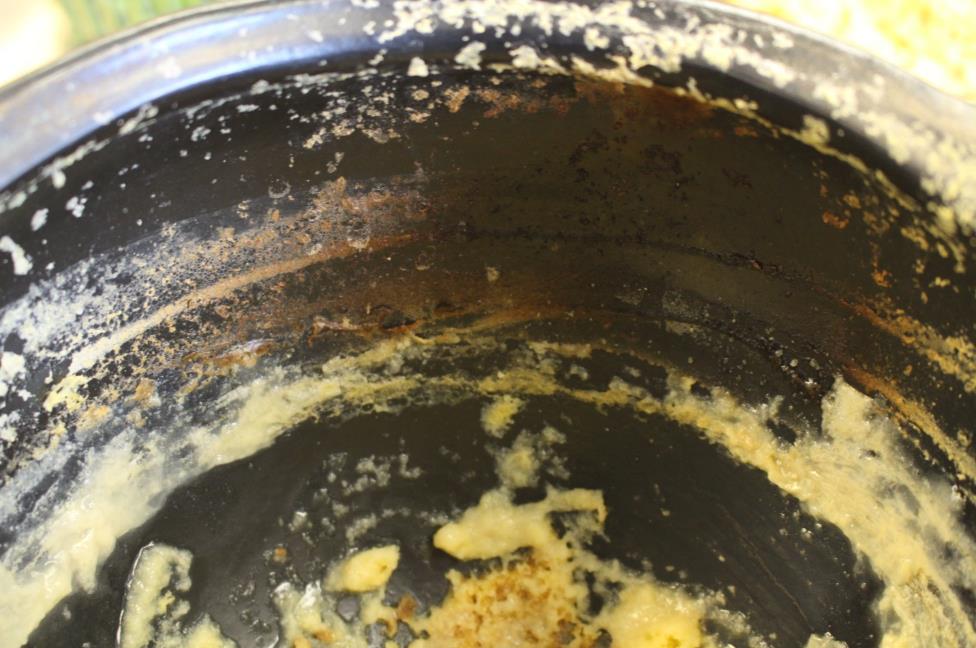 |
| --- | --- |

Figure 2c figure 2d

| 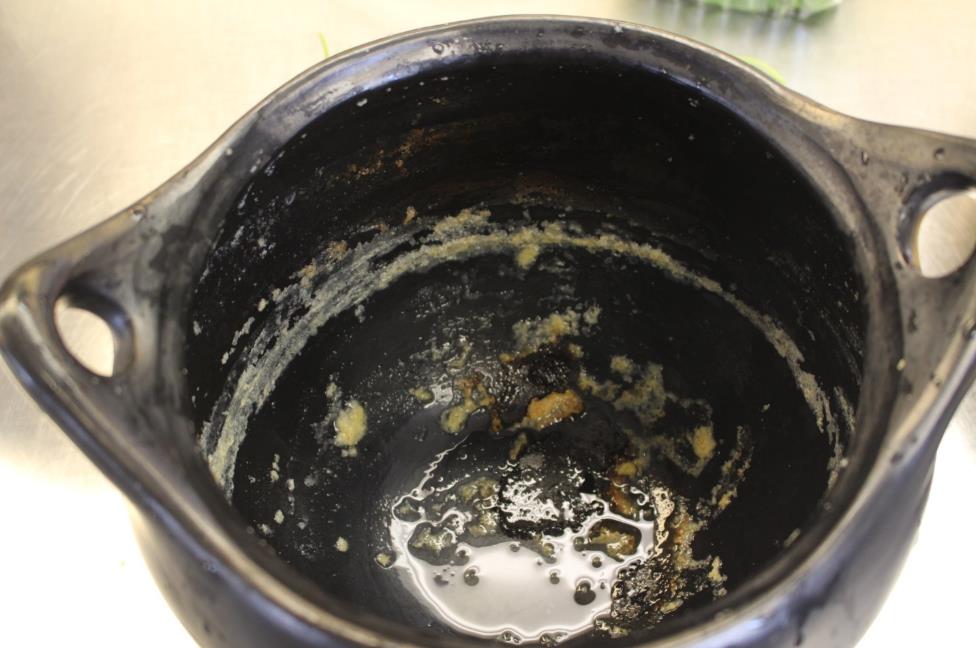 | 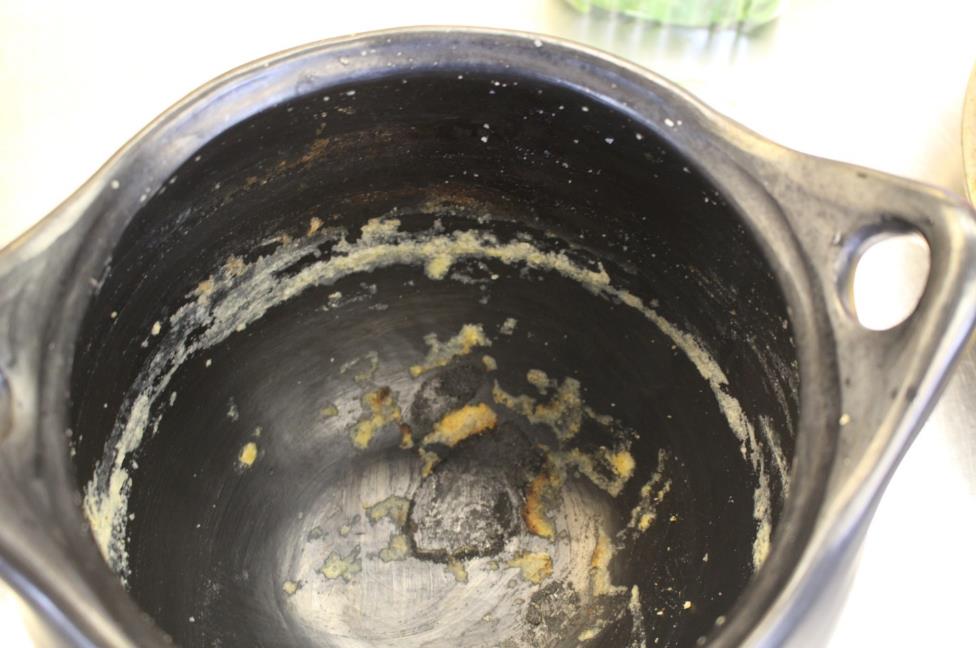 |
| --- | --- |

Figure 2e figure 2f

1. Whole grain millet eggplant and pork stew

Diced eggplant (1 part), pork mince (1part) and millet grains (80% of the mixture) where lightly fried (figure 3a), covered in water and exposed to a side flame. It was then boiled and reduced for 45 minutes (figure 3b), making a thick moist grain dish (figure 3c). Internal carbonization could be seen on both sides, under water line and down to base (though water line was barely visible, figures 3d&e). External oxidization somewhat similar to the observed pattern in other experiments formed, though less pronounced (figure 3f) – possible due to liquid content preventing the vessel from reaching higher temperature along the vessel walls (and see Skibo 1991).

| 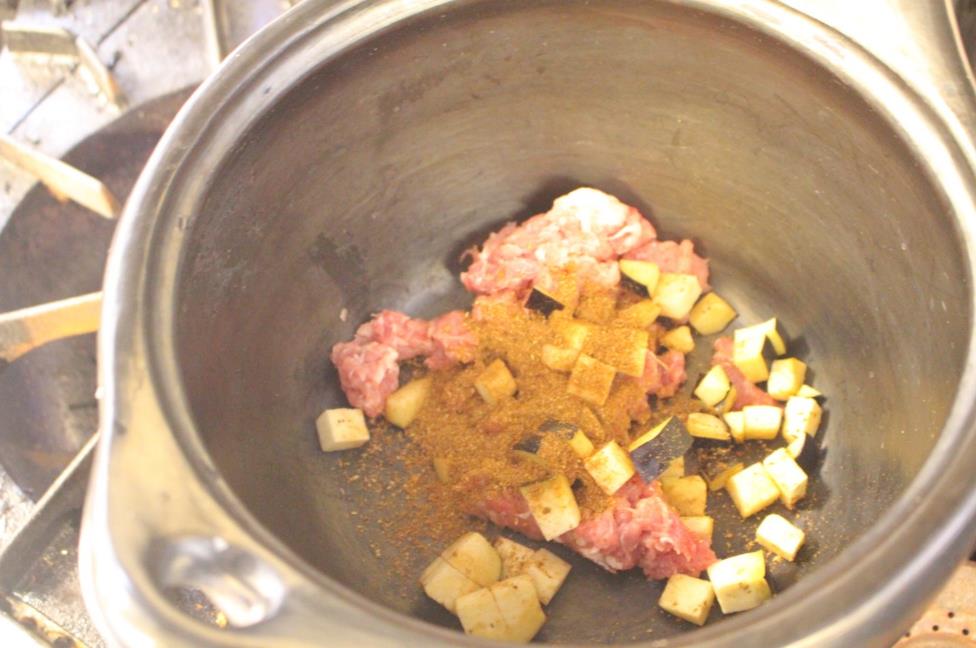 | 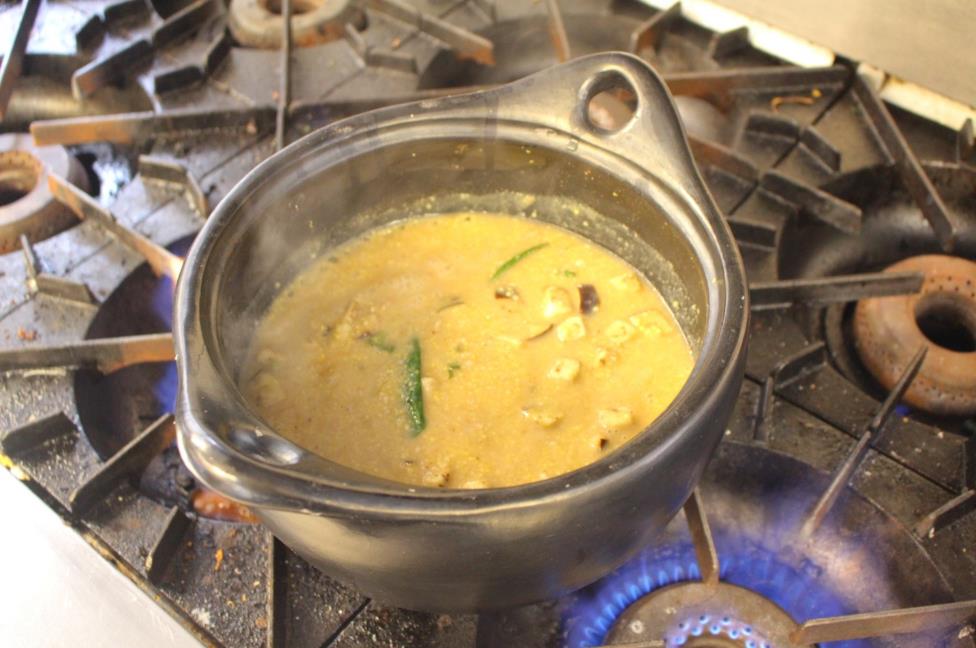 |
| --- | --- |

Figure 3a figure 3b

| 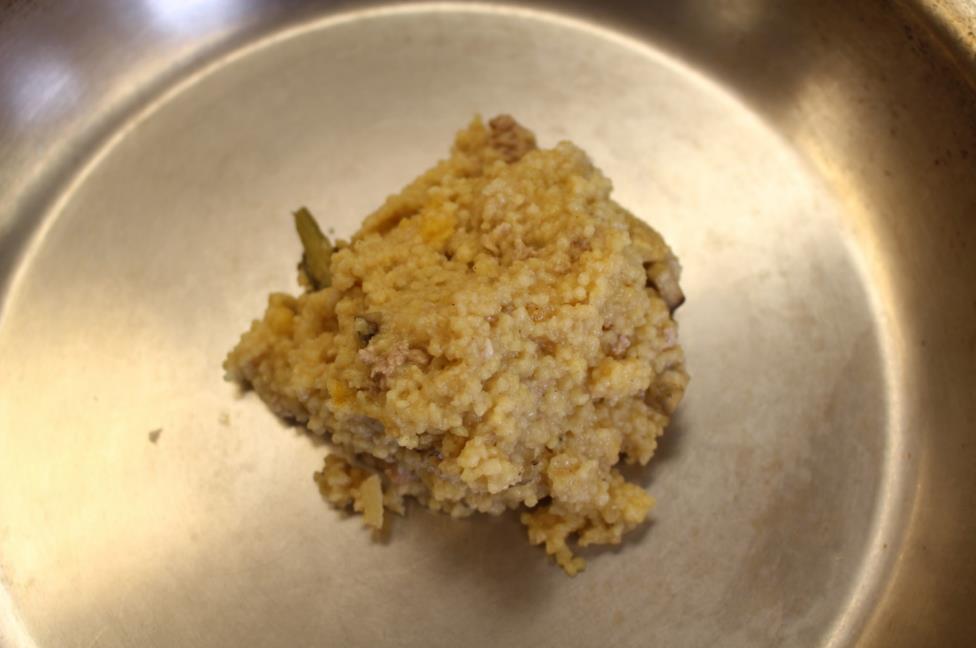 | 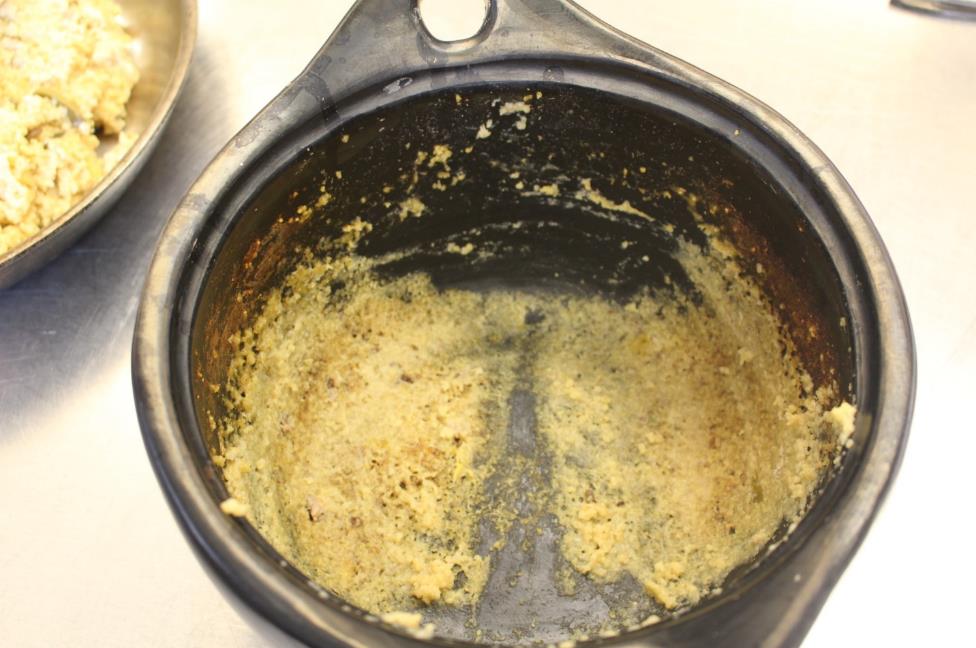 |
| --- | --- |

Figure 3c figure 3d

| 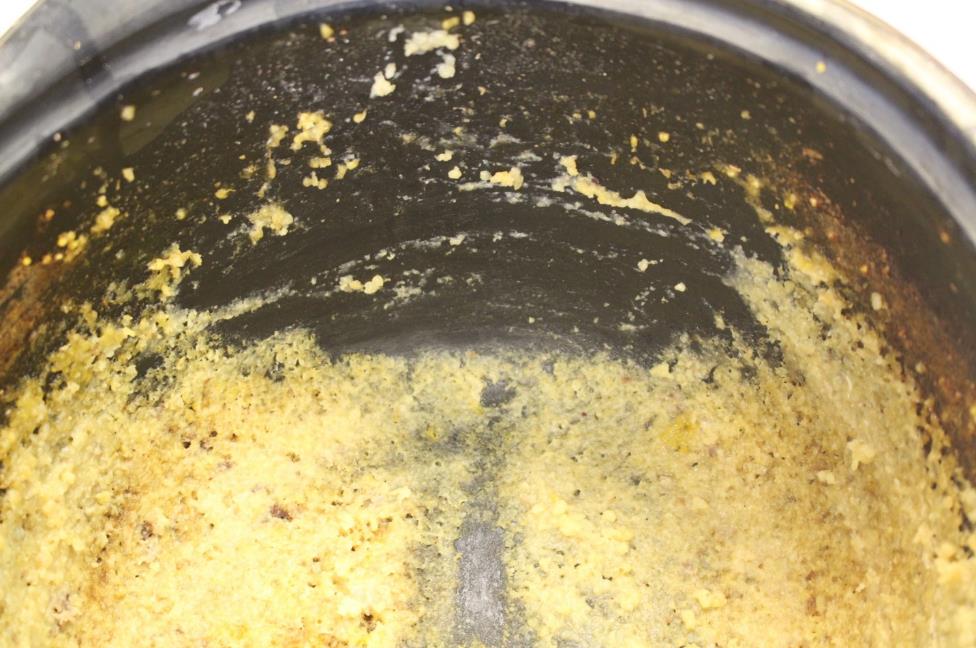 | 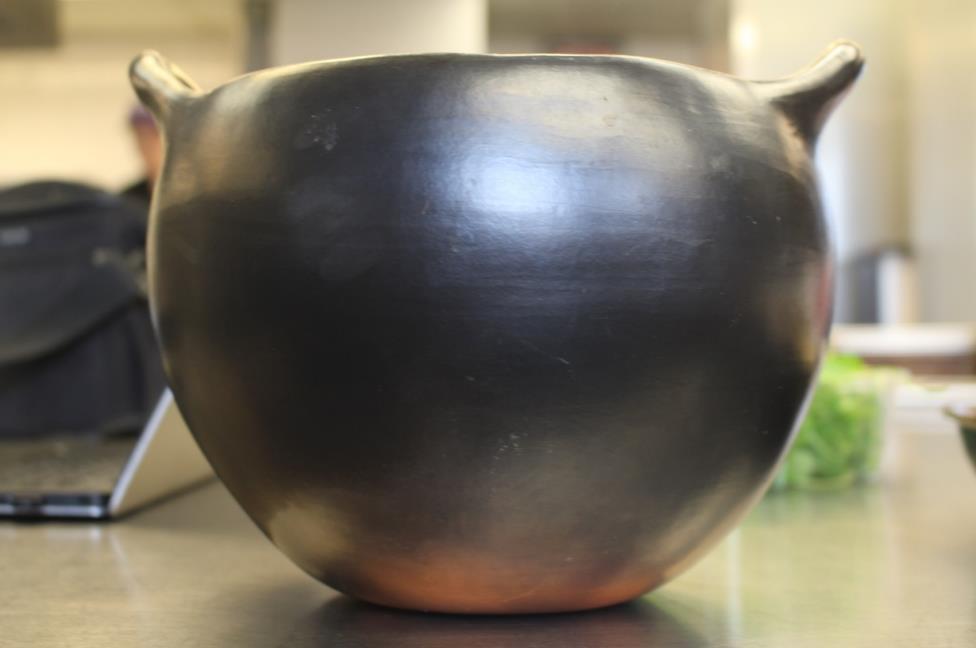 |
| --- | --- |

Figure 3e figure 3f

1. Frying millet cakes in oil with pot placed on side

Millet flour and water were combined to make dough and shape cakes. Pot was placed on its side with oil added and cakes fried in it (figures 4a&b). After initial frying, we added water and reduced (figure 4c) for at least 40 minutes to make millet cake (figure 4d). This reduction creates internal water line (figure 4e) and external spherical oxidation mark mirroring the shape of the flame (figure 4f).

| 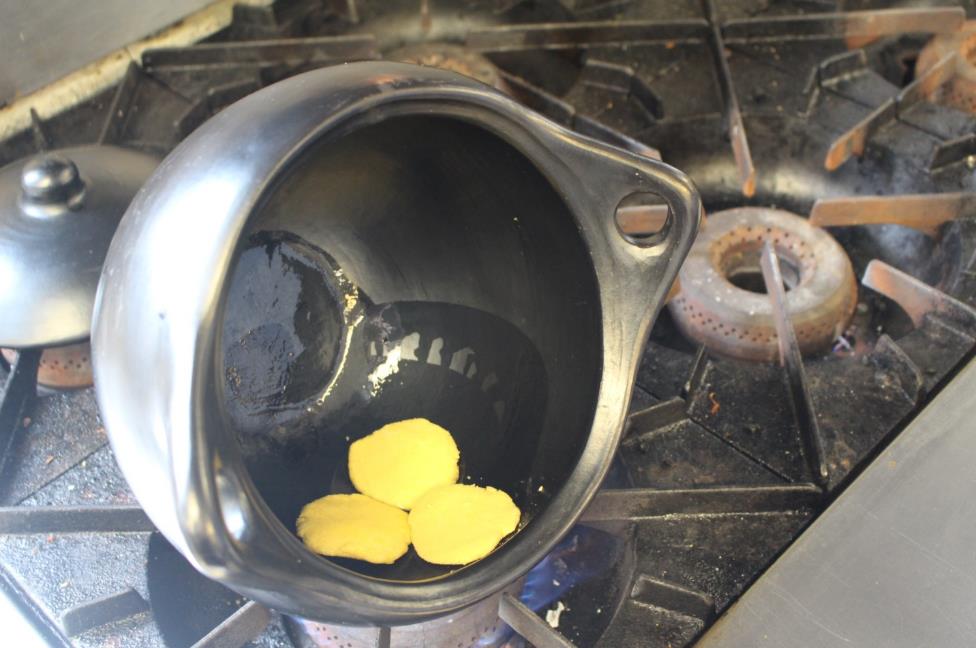 | 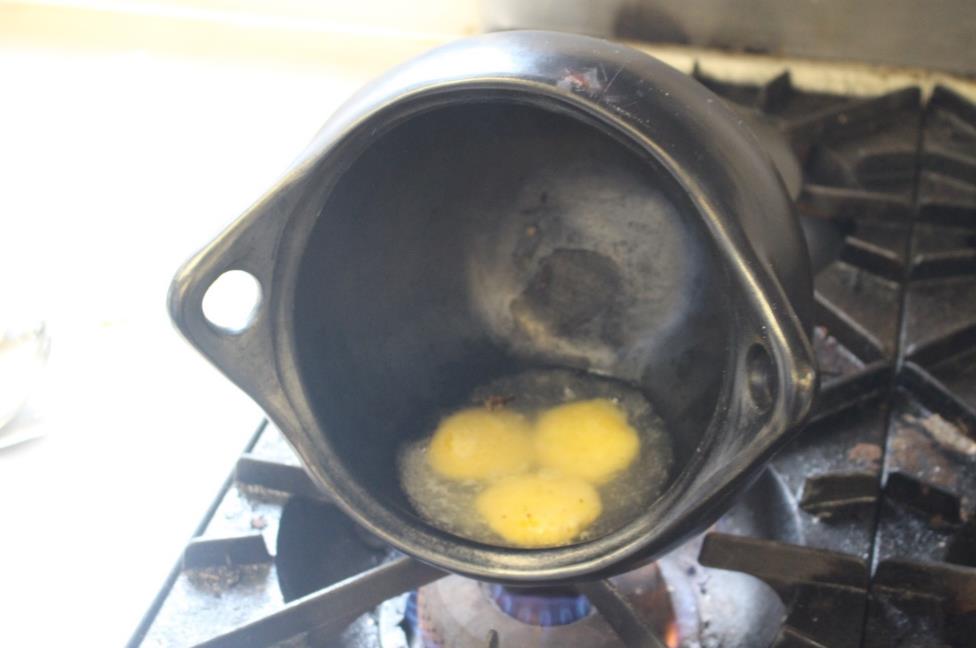 |
| --- | --- |

Figure 4a figure 4b

| 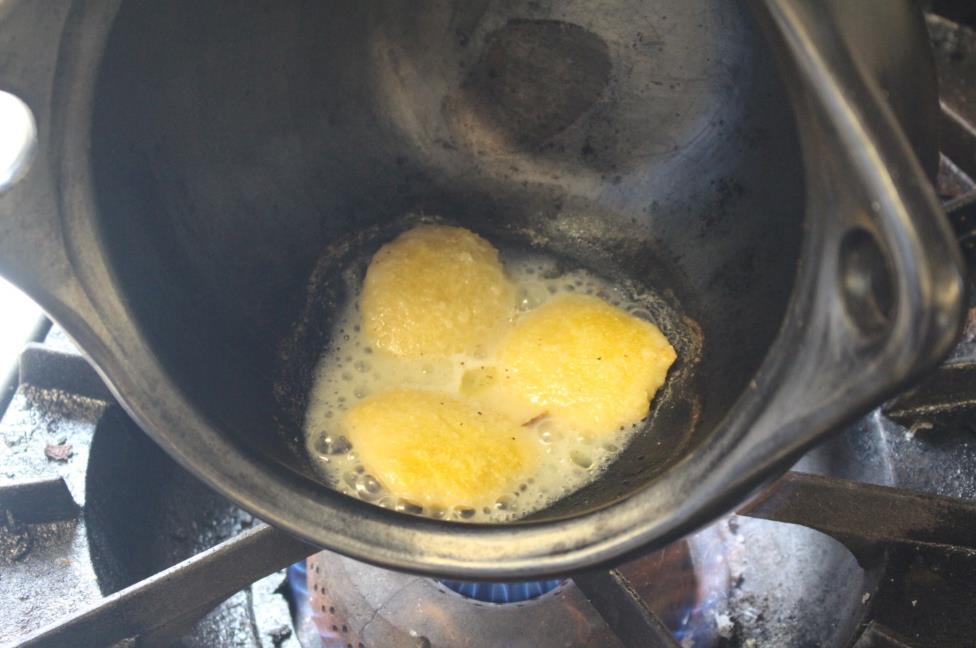 | 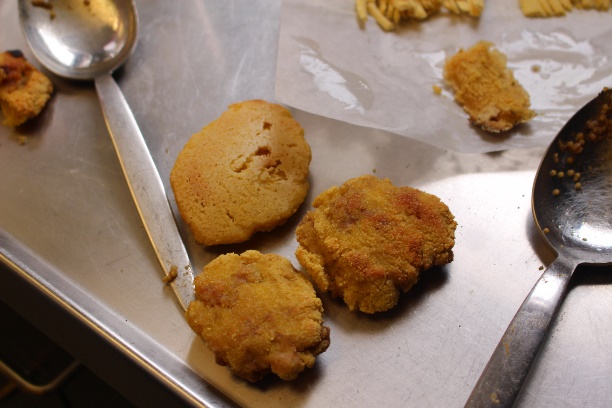 |
| --- | --- |

Figure 4c figure 4d

| 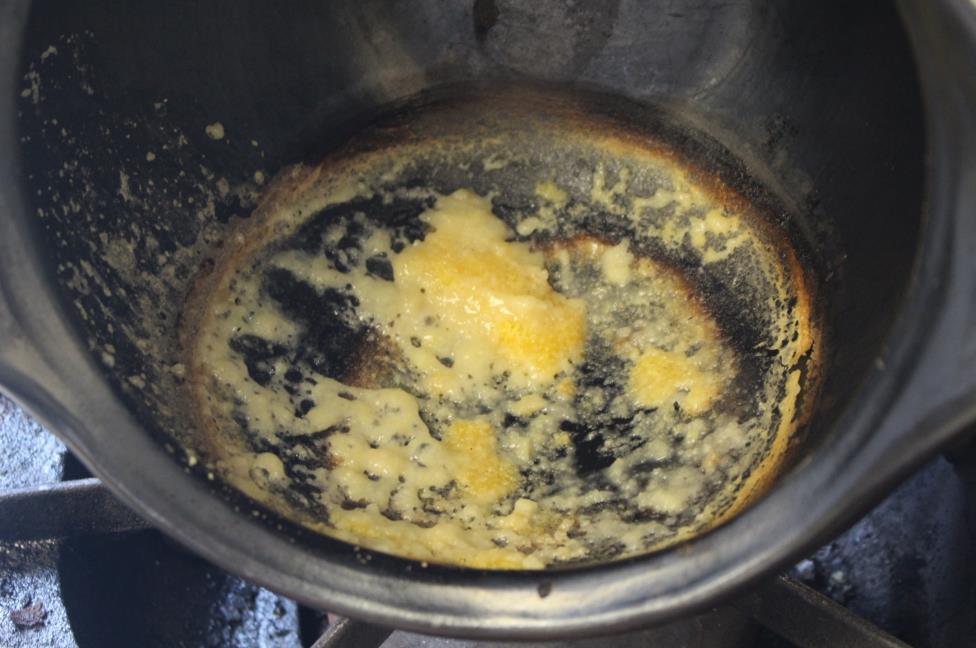 | 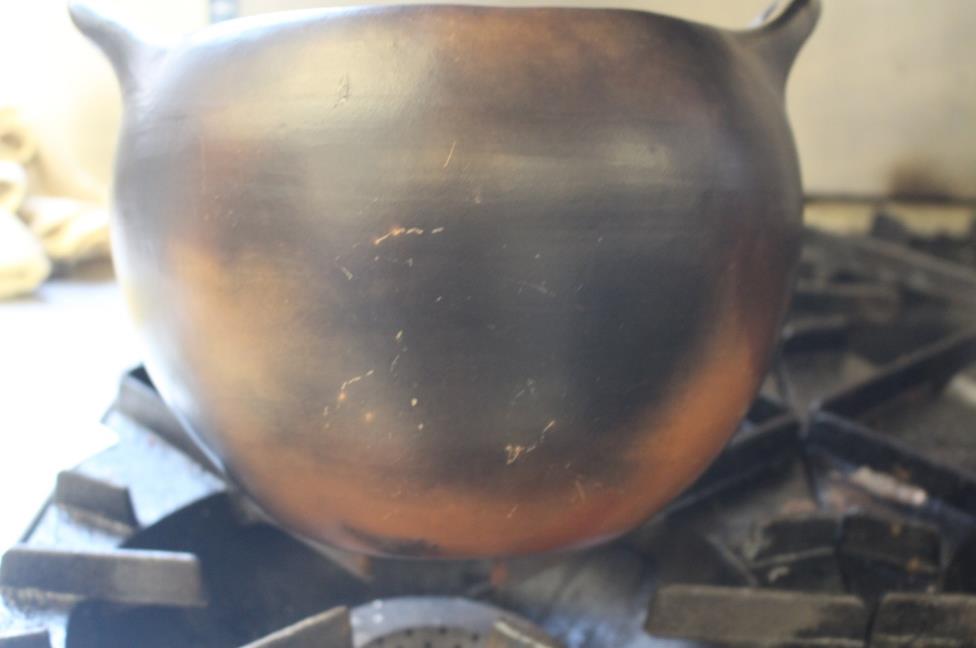 |
| --- | --- |

Figure 4e figure 4f

1. Whole grin millet eggplant and pork stew –starting with pot placed on side

Diced eggplant (1 part), pork mince (1part) and millet grains (80% of the mixture) were lightly fried in oil with vessel placed on its side (figure 5a). When browned nicely and meat juices reduced (figure 5b), we placed back the pot bottom side down, added water and exposed to flame on side only (5c). After 45 minutes of reduction, internal carbonization could be seen only on vessel wall from neck down to base but the base was clear (5d). No water line was observed.

| 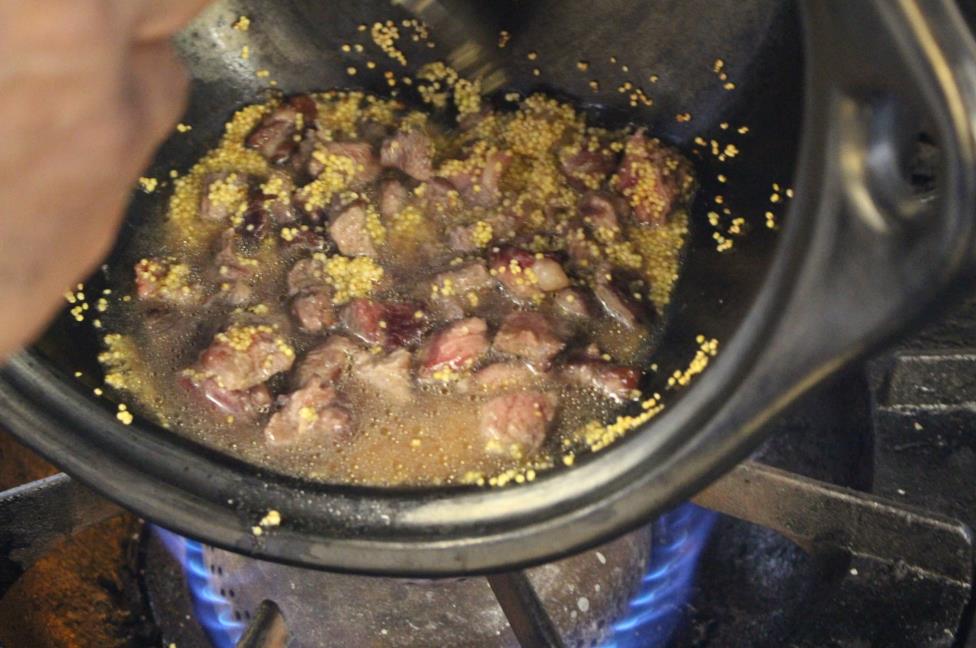 | 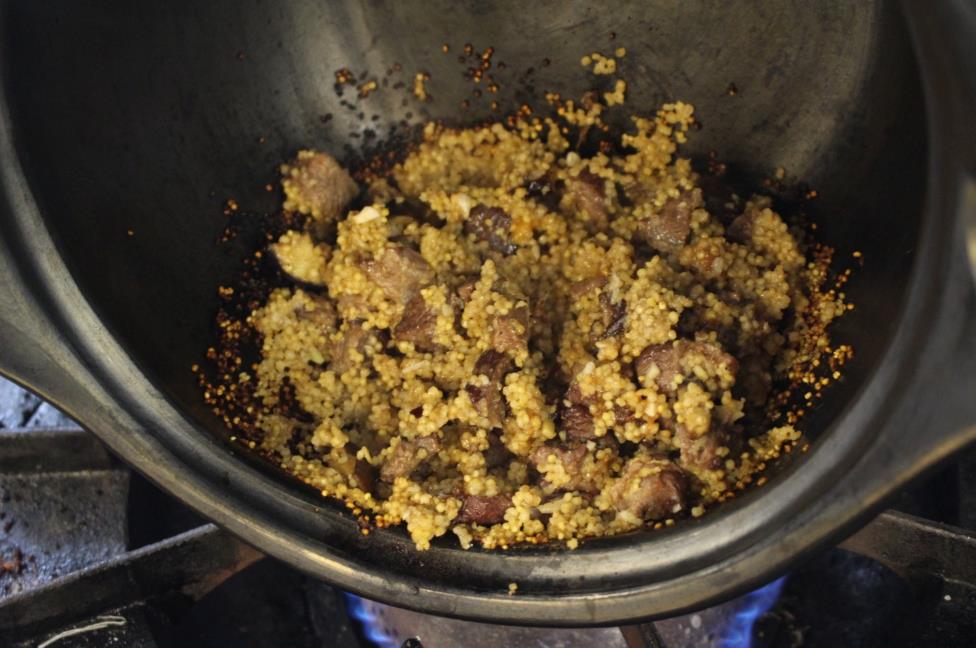 |
| --- | --- |

Figure 5a figure 5b

| 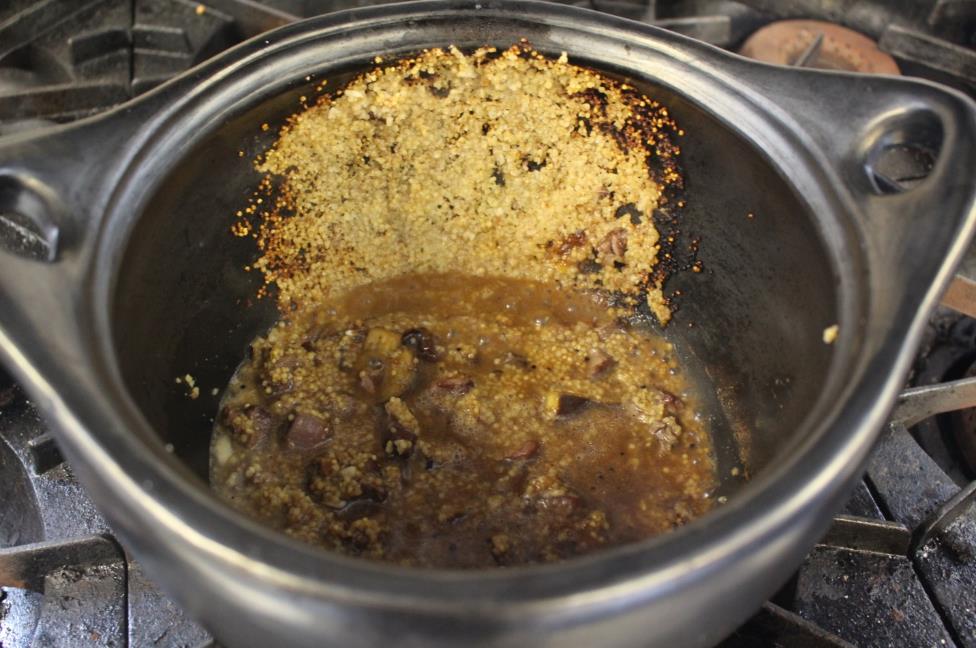 | 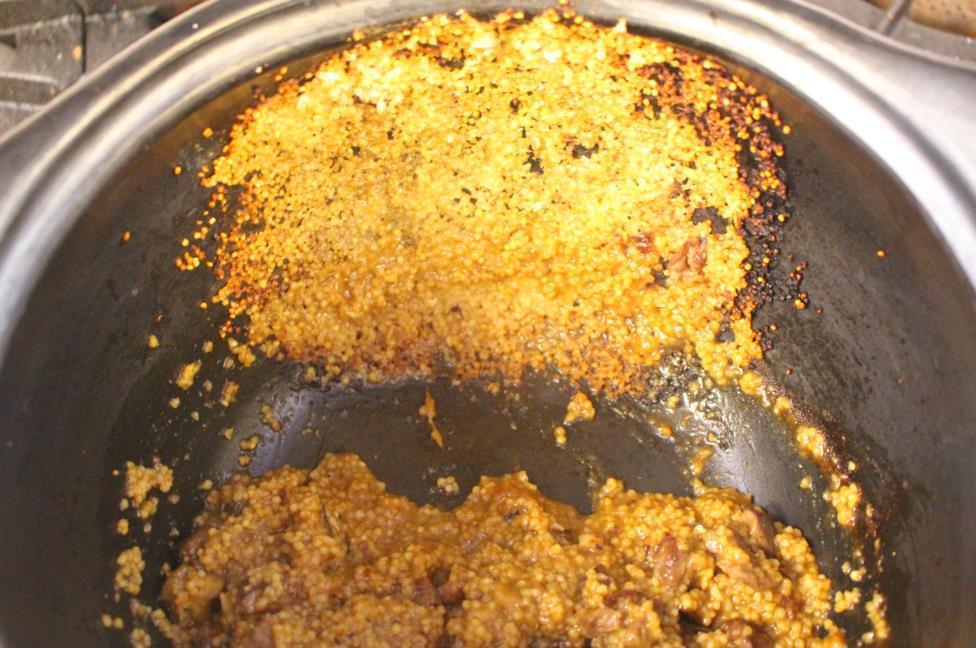 |
| --- | --- |

Figure 5c figure 5d

1. Millet ‘flat bread’ –vessel placed on side.

Millet flour and water were combined to make dough. We heated the pot and stuck the cake on one side (figure 6a). The cake reached hardness after several minutes and can be flipped to complete cooking on the other side (figure 6b). The result is a cracker-like cake (figure 6c&d). Internally, a distinct carbonization pattern can be seen from rim to base (but not including the base –figure 6e). Externally, no distinct pattern could be deduced (figure 6f)

| 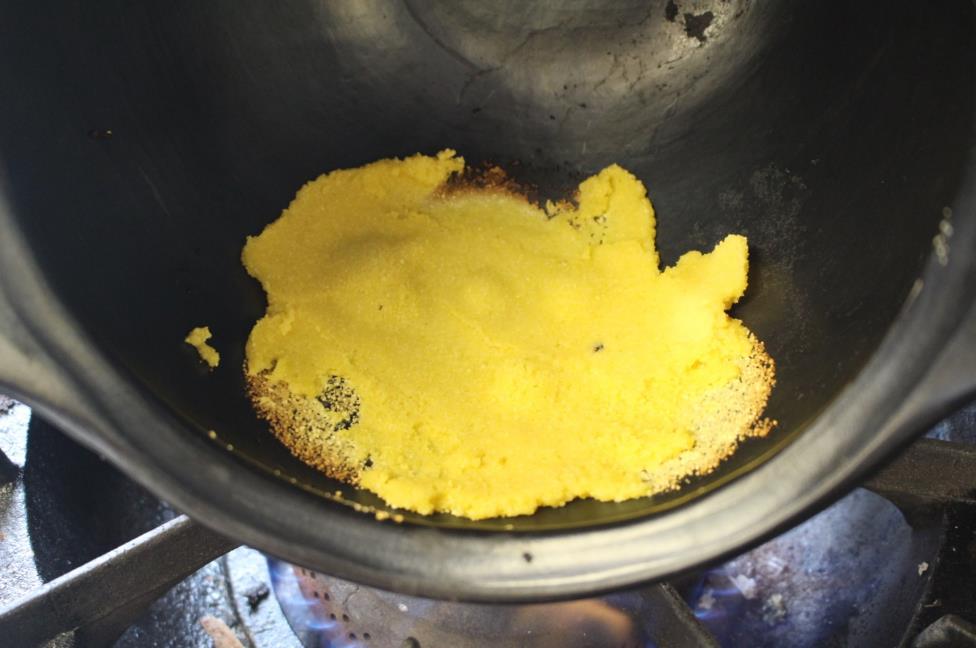 | 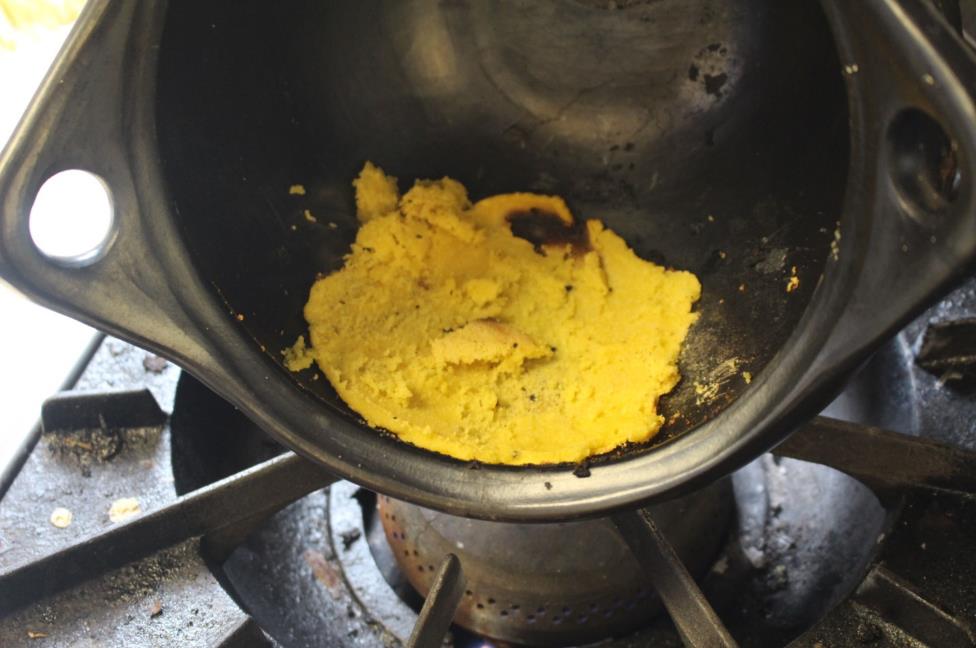 |
| --- | --- |

Figure 6a figure 6b

| 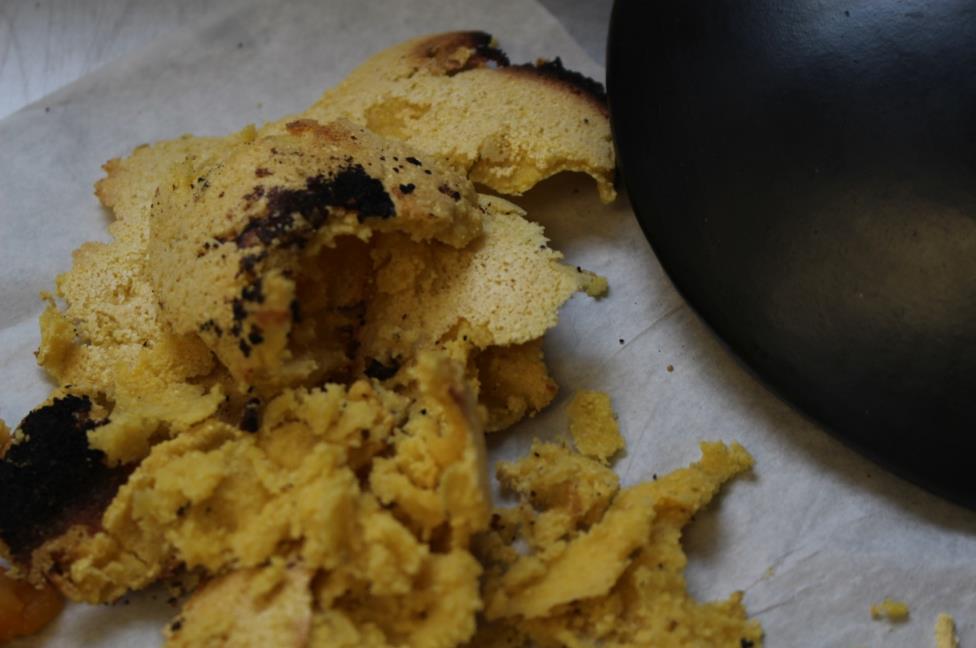 | 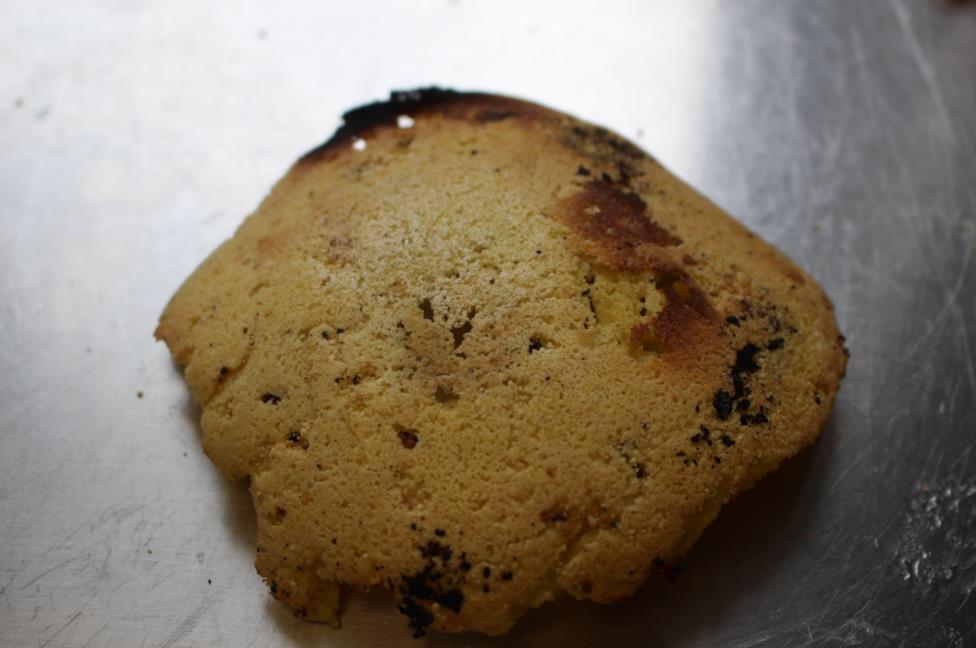 |
| --- | --- |

Figure 6c figure 6d

| 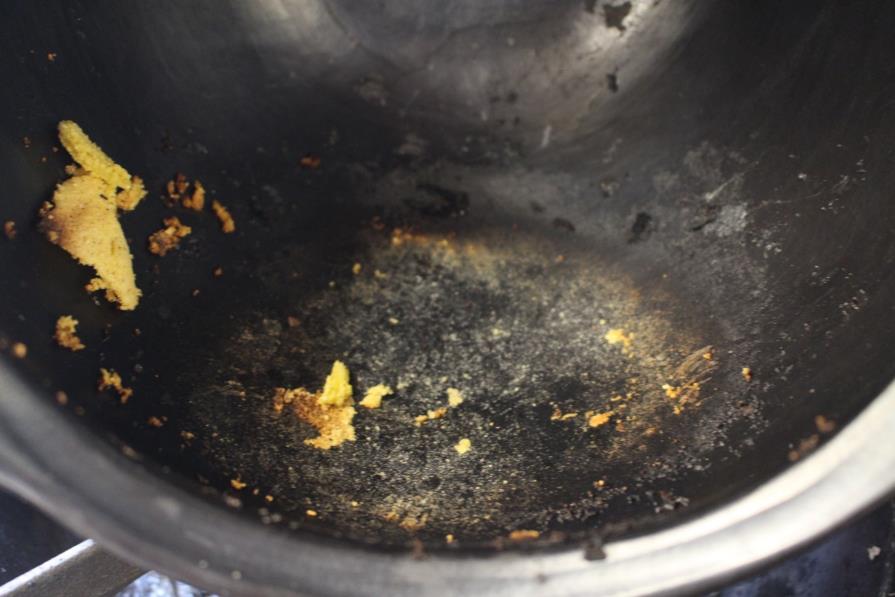 | 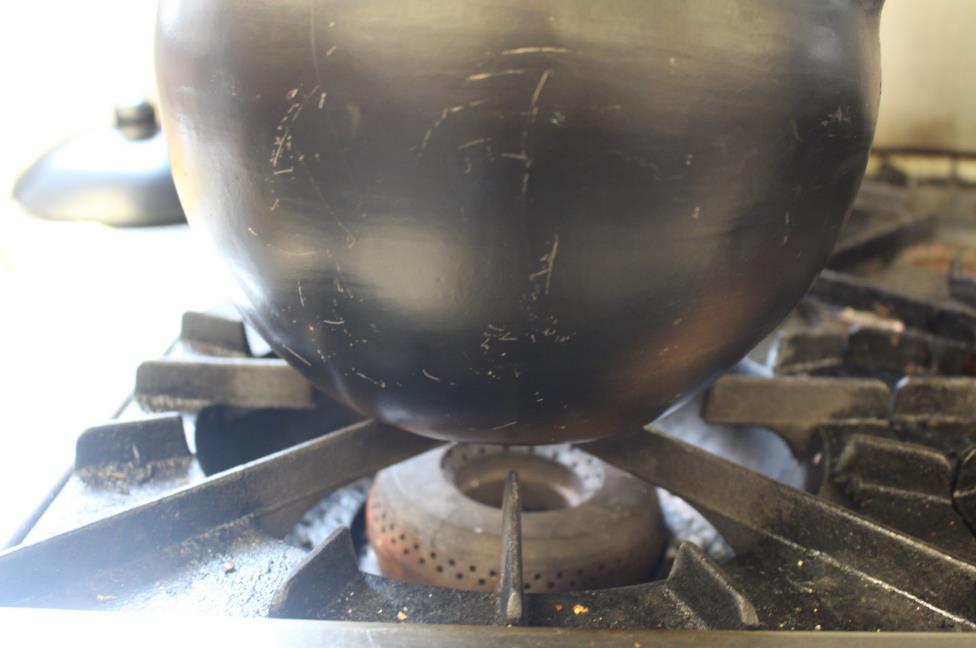 |
| --- | --- |

Figure 6e figure 6f

Summary - Our experiments were geared towards understanding how the unique pattern observed on Siwa ceramic vessels accumulated on the vessel both internally and externally. We experimented with whole-grain millet and millet flour dishes cooked in water and varying degrees of heat sources and intensities. Our experimentation showed that if placed bottom side down (experiments #1-3) the external horseshoe oxidation pattern observed on the *ma’an* jars could be replicated, though this formed on the base as well. Internally, this cooking method resulted in carbonization along the vessel walls exposed to the heat source as well as a water line when liquid was used (as in experiments 1&2 where the liquid prevented much else than a water line). Carbonization occurred on the base as well when no liquid was used (as in experiment 3 where a stew of meat millet and veg was made). When the pot was placed on its side in the flame (experiments 4-6), it resulted in an internal water line and an external rounded oxidation pattern around the wall where the flames were when water was used to cook food only on the side (experiment 4). But when liquid was added to the dish with the vessel placed bottom side down next to the flame (experiment 5 millet, meat and veg stew), the Siwa *ma’an* vessel usewear pattern was fully replicated: externally a horseshoe oxidation pattern was seen from the base up to the shoulder and internally a clear oxidation pattern formed along the vessel walls from rim to base – not including the base. When millet cakes where baked/fried on the side (experiment 6), internally the same carbonization pattern was seen but externally an indistinct oxidation patter was observed.

**Bibliography**

Miller, M.J., Whelton, H.L., Swift, J.A. *et al.* Interpreting ancient food practices: stable isotope and molecular analyses of visible and absorbed residues from a year-long cooking experiment. *Sci Rep* **10,** 13704 (2020). https://doi.org/10.1038/s41598-020-70109-8
